# Zizaane-Type Sesquiterpenoids and Their Rearranged Derivatives from Agarwood of an *Aquilaria* Plant

**DOI:** 10.3390/molecules27010198

**Published:** 2021-12-29

**Authors:** Jing-Zhe Yuan, Yi-Ling Yang, Wei Li, Li Yang, Hao-Fu Dai, Attila Mándi, Cai-Hong Cai, Hui-Qin Chen, Wen-Hua Dong, Tibor Kurtán, Wen-Li Mei, Hao Wang

**Affiliations:** 1 School of Life Sciences, Hainan University, Haikou 570228, China; yuanjingzhenpc@126.com; 2 Hainan Engineering Research Center of Agarwood, Institute of Tropical Bioscience and Biotechnology, Chinese Academy of Tropical Agricultural Sciences, Haikou 571101, China; yangyil@staff.uni-marburg.de (Y.-L.Y.); liwei@itbb.org.cn (W.L.); yangli@itbb.org.cn (L.Y.); daihaofu@itbb.org.cn (H.-F.D.); caicaihong@itbb.org.cn (C.-H.C.); chenhuiqin@itbb.org.cn (H.-Q.C.); dongwenhua@itbb.org.cn (W.-H.D.); 3 Department of Organic Chemistry, University of Debrecen, P.O. Box 400, H-4002 Debrecen, Hungary; mandi.attila@science.unideb.hu (A.M.); kurtan.tibor@science.unideb.hu (T.K.)

**Keywords:** agarwood, *Aquilaria* sp., sesquiterpenoid, zizaane, TDDFT-ECD, anti-inflammatory effect

## Abstract

Nine new sesquiterpenoids (**1**–**9**) were isolated from ethyl ether extract of agarwood originated from *Aquilaria* sp., including three novel sesquiterpenoids (**1**–**3**) derived from zizaane, together with six zizaane-type sesquiterpenoids (**4**–**9**). All structures were unambiguously elucidated based on 1D and 2D NMR spectra as well as by HRESIMS data. The absolute configuration of sesquiterpenoids was determined by comparison of the experimental and computed ECD spectra. In vitro anti-inflammatory assessment showed that compound **9** exhibited inhibition of NO production in LPS-stimulated RAW264.7 cells with an IC_50_ value of 62.22 ± 1.27 μM.

## 1. Introduction

**Figure 1:**
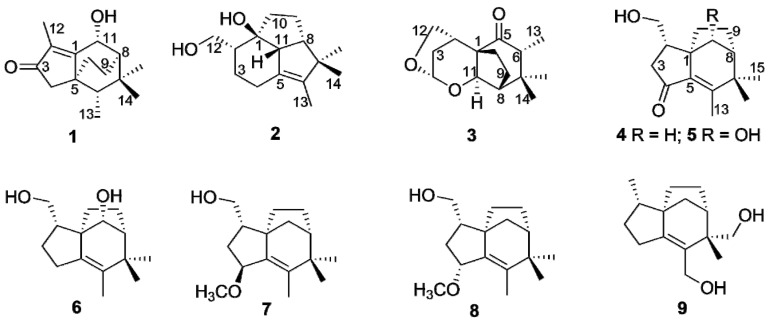
Structures of sesquiterpenoinds **1**–**9** from agarwood.

Agarwood is produced inside *Aquilaria* and *Gyrinops* trees as a self-treatment mechanism to suppress various forms of injury, such as chopping, holing, nailing, microbial infection, etc. [1]. Chemical studies revealed that sesquiterpenes and 2-(2-phenethyl)chromones are two main types of components [2]. As major volatile constituents, the sesquiterpenes from agarwood exhibit various types, including agarofurans, agarospiranes, guaianes, eudesmanes, eremophilanes, cadinanes, prezizaanes, zizaanes, acoranes, etc., which contribute to the smell and pharmacological properties of agarwood [1]. In our previous studies, plenty of tricyclic prezizaanes, three zizaanes and several sesquiterpenes bearing 11-methyl ester groups were identified from agarwood originated from *Aquilaria* sp. [3,4,5]. Some of them exhibit potent *α*-glucosidase and acetylcholinesterase inhibition activities. In continuation of identifying bioactive constituents from the agarwood originating from *Aquilaria* sp., nine previous unreported sesquiterpenoids (**1**–**9**) were identified, including three novel sesquiterpenoids (**1**–**3**) derived from zizaanes (Figure 1). All structures were elucidated on the basis of HRESIMS, 1D and 2D NMR spectroscopic analyses. Their absolute configuration was determined by comparison of the experimental and computed ECD spectra. Herein, this paper describes the isolation, structural elucidation as well as bioactivities of nine sesquiterpenoids. 

## 2. Results and Discussion

**Figure 2:**
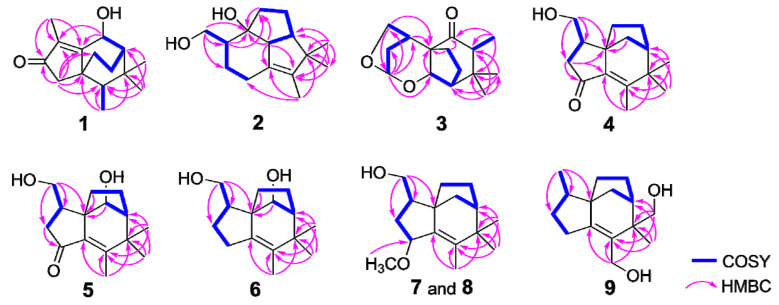
Key COSY and HMBC correlations for compounds **1**–**9**.

**Figure 3:**
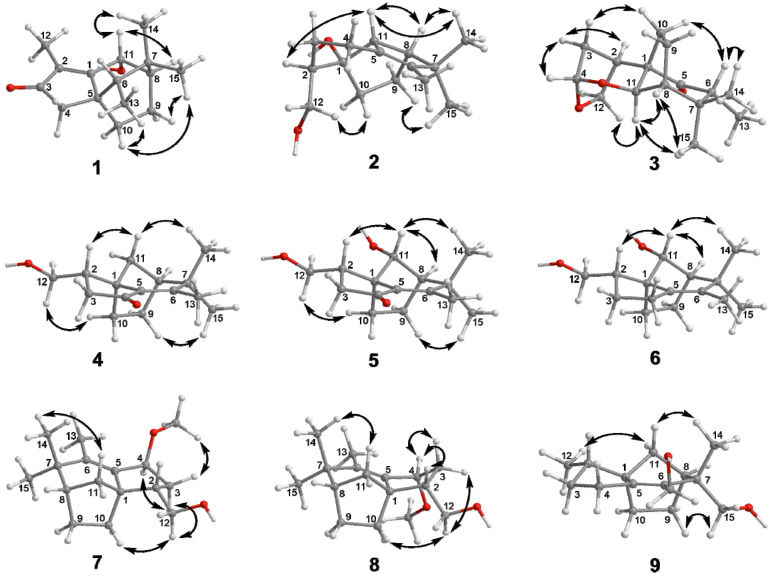
Key NOE correlations of compounds **1**–**9**.

Compound **1** was obtained as a colorless bulk crystal. The molecular formula of **1** was deduced to be C_15_H_22_O_2_ by HRESIMS analysis, requiring five degrees of unsaturation. The ^1^H NMR spectrum (Table 1) exhibited four methyl groups at *δ*_H_ 1.84 (Me-12), 1.01 (Me-15), 0.99 (Me-14) and 0.92 (Me-13) and an oxygenated methine at *δ*_H_ 4.99 (H-11). Its ^13^C NMR (Table 2) showed 15 carbon resonances including five quaternary carbons (one carbonyl carbon at *δ*_C_ 209.4 (C-3) and two olefinic carbons at *δ*_C_ 179.3 (C-1) and 134.4 (C-2)), three methines (including an oxygenated methine at *δ*_C_ 66.5 (C-11)), three methylene and four methyl groups as deduced by the DEPT and HSQC experiments. The presence of tricyclic structure in **1** was deduced by the remaining three degrees of unsaturation. The ^1^H-^1^H COSY correlations (Figure 2) between H-11/H-8 (*δ*_H_ 1.51)/H_2_-9 (*δ*_H_ 1.80 and 1.75)/H_2_-10 (*δ*_H_ 1.98 and 1.13), Me-13/H-6 (*δ*_H_ 1.22) suggested the existence of two sequences to be C-11/C-8 (*δ*_C_ 46.4)/C-9 (*δ*_C_ 39.0)/C-10 (*δ*_C_ 25.0) and C-13 (*δ*_C_ 11.5)/C-6 (*δ*_C_ 43.9). The connection of the above two sequences was determined by HMBC correlations from both Me-14 and Me-15 to C-6, C-7 (*δ*_C_ 34.1) and C-8. Furthermore, HMBC correlations (Figure 2) from Me-12 to C-1 (*δ*_C_ 179.3), C-2 (*δ*_C_ 134.4) and C-3 (*δ*_C_ 209.4), and from H_2_-4 (*δ*_H_ 2.14 and 2.02) to C-1, C-2, C-3, C-5 (*δ*_C_ 44.7), C-6 and C-10 indicated the occurrence of a 5-membered ring from C-1 to C-5 with a methyl at C-2, and the connection sequence of C-10/C-5/C-6. The planar structure of **1** was finally elucidated by HMBC correlations from H-11 to C-1 and C-2 as shown. The relative configuration of **1** was determined by NOE correlations between Me-15/H-9a (*δ*_H_ 1.80), Me-15/H-10a (*δ*_H_ 1.98), H-10a/Me-13, Me-14/H-11 and H-11/H-8 in the ROESY spectrum, which suggested that H-6, H-11, H-8 and Me-14 occurred on the same side of the ring, while Me-13, Me-15, H_2_-9 and H_2_-10 were oriented to the opposite face (Figure 3). The absolute structure of **1** was determined by comparison of the experimental and simulated electronic circular dichroism (ECD) spectra (*vide infra*).

Compound **2** was gained as a colorless bulk crystal. It possessed a molecular formula of C_15_H_24_O_2_ with four degrees of unsaturation as established by HRESIMS. The ^1^H and ^13^C NMR data (Table 1 and Table 2), together with DEPT and HSQC spectra of **2** displayed the presence of four quaternary carbons including one double bond, three methines, five methylenes (one oxygenated) and three methyl groups, indicated three-ringed structure in **2** for the remaining three degrees of unsaturation. ^1^H-^1^H COSY cross-peaks between H_2_-12 (*δ*_H_ 3.82 and 3.40)/H-2 (*δ*_H_ 1.98)/H_2_-3 (*δ*_H_ 1.88 and 0.90)/H_2_-4 (*δ*_H_ 2.43 and 1.74), H-11 (*δ*_H_ 2.70)/H-8 (*δ*_H_ 2.37)/H_2_-9 (*δ*_H_ 1.86 and 1.75)/H_2_-10 (*δ*_H_ 1.46 and 1.27) revealed that **2** possessed two spin coupling systems as C-12 (*δ*_C_ 65.2)/C-2 (*δ*_C_ 48.3)/C-3 (*δ*_C_ 27.9)/C-4 (*δ*_C_ 26.1) and C-11 (*δ*_C_ 62.5)/C-8 (*δ*_C_ 51.4)/C-9 (*δ*_C_ 26.0)/C-10 (*δ*_C_ 31.4) (Figure 2). The HMBC correlations from H_2_-12 to C-1 (*δ*_C_ 85.4), C-2 and C-3, and from both H-2 and H-11 to C-1 and C-10 revealed that the linkage of the two sequences was formed from C-10, C-11 and C-2 to C-1. The planar structure of **2** was established by HMBC correlations from H-11 to C-5 (*δ*_C_ 133.7) and C-6 (*δ*_C_ 138.5), from Me-13 (*δ*_H_ 1.47) to C-5, C-6 and C-7 (*δ*_C_ 49.5), from both Me-14 (*δ*_H_ 0.96) and Me-15 (*δ*_H_ 1.01) to C-6, C-7 and C-8, and from H_2_-4 to C-5 and C-6. The NOE relationships between Me-14/H-11, Me-14/H-8, H-8/H-11 and H-11/H-2 of **2** indicated that those protons were oriented on the same face, whereas the NOEs between Me-15/H-9a (*δ*_H_ 1.86) and H_2_-10/H_2_-12 suggested that these latter protons were oriented towards the opposite side (Figure 3).

Compound **3** was gained as a colorless oil. It possessed the molecular C_15_H_22_O_3_ with five degrees of unsaturation as established by HRESIMS. The ^1^H NMR spectrum of **3** (Table 1) exhibited three methyls at *δ*_H_ 0.85 (Me-15), 0.92 (Me-13) and 1.13 (Me-14), an oxygenated methylene (*δ*_H_ 4.02 and 3.90, H_2_-12), and five methines at *δ*_H_ 5.30 (H-4), 4.08 (H-11), 2.68 (H-2), 2.51 (H-6) and 1.94 (H-8). The ^13^C NMR spectra of **3** (Table 2) displayed the presence of three quaternary carbons including one carbonyl carbon, five methines, four methylenes (one oxygenated) and three methyl groups as edited by the DEPT and HSQC experiments. The sequences of C-12 (*δ*_C_ 73.8)/C-2 (*δ*_C_ 35.9)/C-3 (*δ*_C_ 34.7)/C-4 (*δ*_C_ 101.0), C-11 (*δ*_C_ 78.7)/C-8 (*δ*_C_ 54.5)/C-9 (*δ*_C_ 25.9)/C-10 (*δ*_C_ 30.6) and C-6 (*δ*_C_ 48.9)/C-13 (*δ*_C_ 8.7) were assigned by COSY correlations between H_2_-12/H-2/H_2_-3 (*δ*_H_ 2.25 and 1.59)/H-4, H-11/H-8/H_2_-9 (*δ*_H_ 2.21 and 2.16)/H_2_-10 (*δ*_H_ 2.14 and 1.63) and H-6/Me-13 respectively (Figure 2). HMBC correlations from Me-13 to C-5 (*δ*_C_ 214.6), C-6 and C-7 (*δ*_C_ 39.0), from both Me-15 and Me-14 to C-6, C-7 and C-8, from H-2, H-11 and H_2_-10 to C-1 (*δ*_C_ 60.2), from H-4 to C-11, C-2, and C-12, and from H-10b (*δ*_H_ 1.63) to C-5 established the planar structure of **3** with bicyclo [3.2.1]octane core and acetal group at C-4. The relative configuration of **3** was deduced by NOEs between H-11/Me-15, H-11/H-12a (*δ*_H_ 4.02), H-11/H-8, H-6/H-10b (*δ*_H_ 1.63), H-6/Me-14, H-2/H-3b (*δ*_H_ 1.59), H-4/H-3b, and H-3a (*δ*_H_ 2.25)/H-10a (*δ*_H_ 2.14) as shown (Figure 3).

Compound **4** was isolated as a colorless bulk crystal. The molecular formula of compound **4** was determined as C_15_H_26_O_3_ by HRESIMS. The NMR data of **4** (Table 3 and Table 4) were similar to those of albaflavenone, except for the presence of oxygenated methylene (*δ*_H_ 3.75, 3.63 and *δ*_C_ 63.9, CH_2_-12).^6^ The existence of 12-OH was confirmed by COSY correlations between H_2_-12/H-2 (*δ*_H_ 2.32)/H_2_-3 (*δ*_H_ 2.40 and 2.10) together with HMBC correlations from H_2_-12 to C-1 (*δ*_C_ 52.0), C-2 (*δ*_C_ 42.1) and C-3 (*δ*_C_ 43.8) (Figure 2). The remaining substructures and the relative configuration of **4** were shown to be identical to those of albaflavenone by detailed analysis of the 2D NMR spectra (Figure 2 and Figure 3).

Compound **5** had the molecular formula C_15_H_22_O_3_ according to HRESIMS, indicating that the addition of one further oxygen compared to **4**. The NMR data of **5** (Table 3 and Table 4) were identical to those of **4**, except for the presence of oxygenated methine (*δ*_H_ 3.89 and *δ*_C_ 79.1, CH-11). The attachment of hydroxy group at C-11 was elucidated by key HMBC correlations from H-11 to C-9 (*δ*_C_ 23.1) and C-10 (*δ*_C_ 27.7). The NOE cross-peaks from H-11 to H-2 (*δ*_H_ 2.44), H-8 (*δ*_H_ 1.83) and Me-14 (*δ*_H_ 1.23) indicated a *cis* relationship between H-8 and H-11. The remaining structure of **5** was shown to be identical to that of **4** by 2D NMR analysis.

Compound **6** was isolated as a white amorphous powder. The molecular formula of C_15_H_24_O_2_ was deduced from its HRESIMS, differing from **5** by the loss of one oxygen and the addition of two extra protons. Comparison of its ^1^H and ^13^C NMR data (Table 3 and Table 4) with those of compound **5** showed an additional methylene group (*δ*_H_ 2.31, 2.16 and *δ*_C_ 28.2, CH_2_-4) and the disappearance of keto group in **5**, which was confirmed by COSY correlations between H_2_-12 (*δ*_H_ 3.63 and 3.45)/H-2 (*δ*_H_ 2.12)/H_2_-3 (*δ*_H_ 1.75 and 1.30)/H_2_-4. The remaining structure of **6** was shown to be identical to that of **5** by detailed analysis of the 2D NMR spectra of **6**.

Compound **7** was obtained as a colorless oil. It possessed the molecular formula C_16_H_26_O_2_ as determined by HRESIMS. Its ^1^H and ^13^C NMR spectra were similar to those of **4**. The appearance of a methoxy group (*δ*_H_ 3.30 and *δ*_C_ 56.3, 4-OCH_3_) in **7** and the absence of keto group at C-4 in **4** suggested the methoxy group located at C-4, which was confirmed by COSY correlations between H_2_-12 (*δ*_H_ 3.76 and 3.62)/H-2 (*δ*_H_ 2.25)/H_2_-3 (*δ*_H_ 2.09 and 1.34)/H-4 (*δ*_H_ 4.12) together with the HMBC correlations from the methoxy singlet to C-4 (*δ*_C_ 79.7). Detailed analysis of the 2D NMR spectra of **7** determined the compound to be identical to **4** except for the methoxy group at C-4. The coupling constant of H-4 (*δ*_H_ 4.12, d, *J* = 5.7 Hz) was comparable to that of (4*S*)-albaflavenol (*δ*_H_ 4.56, d, *J* = 5.4 Hz), but differed from that in (4*R*)-albaflavenol (*δ*_H_ 4.61, t, *J* = 7.8 Hz), suggesting the *trans* configuration between H-2 and H-4 [6]. In the ROESY spectrum of **7**, the observed NOE correlations between H_2_-12/H-3α (*δ*_H_ 1.34), H-4/H-3α and 4-OCH_3_/H-3β (*δ*_H_ 2.09) confirmed this assumption.

Compound **8** shared the same planar structure as that of **7** by detailed analysis of its HRESIMS and 2D NMR spectrum. The coupling constant of H-4 (*δ*_H_ 4.24, t, *J* = 6.4 Hz) was comparable to that of (4*R*)-albaflavenol (*δ*_H_ 4.61, t, *J* = 7.8 Hz), suggesting the *cis* configuration between H-2 (*δ*_H_ 2.02) and H-4 [6]. Meanwhile, the key NOE correlation between H-2 and H-4 in the ROESY spectrum suggested that **7** and **8** are 4-epimers.

Compound **9** was gained as a colorless oil. It possessed the molecular formula C_15_H_24_O_2_ with four degrees of unsaturation as indicated by HRESIMS. The NMR data of compound **9** resembled those of **4** showed the presence of two oxygenated methylene groups (*δ*_H_ 3.66, 3.62 and *δ*_C_ 69.0, CH_2_-15; *δ*_H_ 4.01, 3.95 and *δ*_C_ 59.4, CH_2_-13) and a doublet methyl signal (*δ*_H_ 0.97 and *δ*_C_ 14.2, Me-12). HMBC correlations from both H_2_-15 and Me-14 (*δ*_H_ 1.08) to C-6 (*δ*_C_ 130.2), C-7 (*δ*_C_ 46.4) and C-8 (*δ*_C_ 44.6), and from H_2_-13 to C-5 (*δ*_C_ 153.9), C-6 and C-7, together with NOE correlation between H_2_-15/H-9a (*δ*_H_ 1.99) indicated that two hydroxy groups located at C-13 and C-15, respectively.

**Figure 4:**
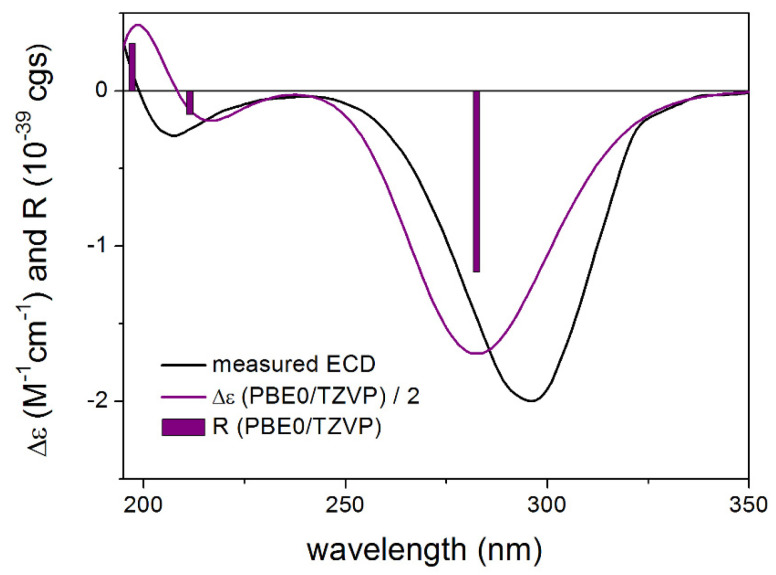
Experimental ECD spectrum of **3** in MeOH compared with the PBE0/TZVP PCM/MeOH ECD spectrum of the single low-energy CAM-B3LYP/TZVP PCM/MeOH conformer of proposed (1*S*,2*S*,4*S*,6*R*,8*R*,11*R*)-**3**. Bars represent the rotational strength values.

To elucidate the absolute configuration of **1**, **3** and **4**, the solution TDDFT-ECD method was applied [7,8]. The initial Merck Molecular Force Field (MMFF) conformers were reoptimized at the B3LYP/6-31+G(d,p) in vacuo and the CAM-B3LYP/TZVP PCM/MeOH levels, separately, and ECD spectra were computed with four different functionals for the low-energy conformers. In the case of proposed (1*S*,2*S*,4*S*,6*R*,8*R*,11*R*)-**3**, a single conformer (Supplementary Materials) was found for which all combinations of levels gave moderate to good agreement with the experimental spectrum based on which the absolute configuration could be unambiguously elucidated as (1*S*,2*S*,4*S*,6*R*,8*R*,11*R*) (Figure 4). It is interesting to note that the B3LYP and PBE0 functionals performed better for the minor transitions in the low-wavelength region then the CAM-B3LYP and BH&HLYP ones.

**Figure 5:**
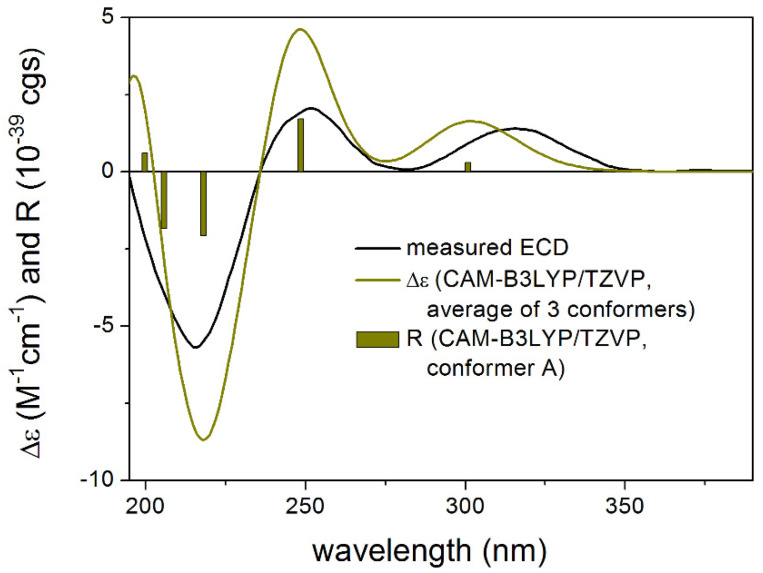
Experimental ECD spectrum of **1** in MeOH compared with the Boltzmann-weighted CAM-B3LYP/TZVP SMD/MeOH ECD spectrum of proposed (5*R*,6*S*,8*R*,11*R*)-**1** computed for the SOGGA11-X/TZVP SMD/MeOH conformers. Bars represent the rotational strength values of conformer A.

In the case of **1**, the B3LYP and PBE0 functionals which performed better for **3** gave a mismatch in all applied combinations for the proposed (5*R*,6*S*,8*R*,11*R*) enantiomer while the BH&HLYP and CAM-B3LYP functionals reproduced all experimental transitions well (Figure 5). Analysis of the distinct conformers indicated that a different Boltzmann-weight can reproduce the experimental spectrum also in the case of the B3LYP and PBE0 functionals which can derive from a moderate error in estimating the relative energies by the applied levels of theories [9,10]. Therefore the initial 3 MMFF conformers were also reoptimized at the SOGGA11-X/TZVP [11]. SMD/MeOH level. The SOGGA11-X functional was found one of the best in a recent DFT benchmark study [12]. Indeed, by computing ECD for the SOGGA11-X conformers (Supplementary Materials), all four applied TDDFT functionals gave good agreement with the experimental spectrum in line with the biosynthetic considerations allowing elucidation of the absolute configuration as (5*R*,6*S*,8*R*,11*R*)-**1**. The relative intensities of the transitions were reproduced better by the BH&HLYP and CAM-B3LYP functionals than by the other two.

**Figure 6:**
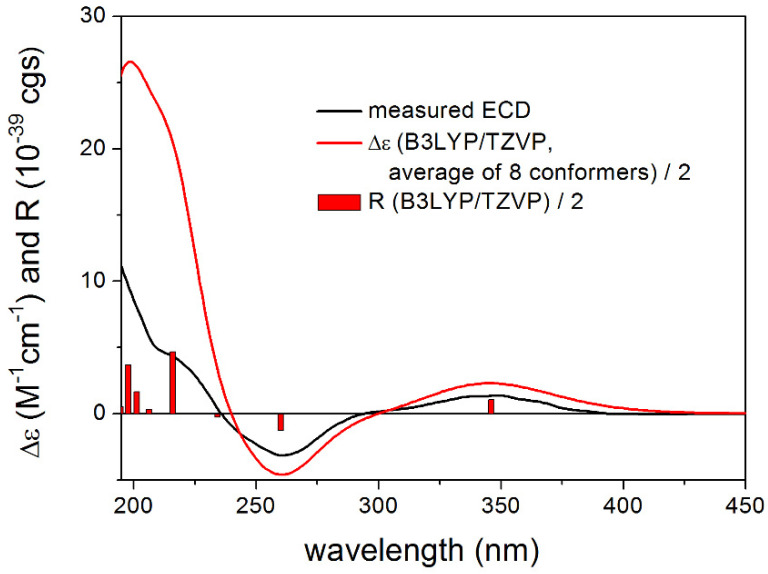
Experimental ECD spectrum of **4** in MeOH compared with the Boltzmann-weighted B3LYP/TZVP PCM/MeOH ECD spectrum of proposed (1*R*,2*S*,8*S*)-**4** computed for the CAM-B3LYP/TZVP PCM/MeOH conformers. Bars represent the rotational strength values of conformer A.

For proposed (1*R*,2*S*,8*S*)-**4**, both the gas-phase and the solvent model calculations gave acceptable to good agreements with the experimental ECD spectrum (Figure 6). Similarly to **1**, ECD spectra computed with the B3LYP and PBE0 functional reproduced all transitions well, while the CAM-B3LYP and especially the BH&HLYP functionals had problems with the reproduction of the negative ECD transition at 260 nm. Reproduction of the other two transitions and nice agreement with the B3LYP and PBE0 functionals, however, allowed elucidation of the absolute configuration as (1*R*,2*S*,8*S*), which is also in line with the biosynthetic considerations. To improve the agreement, DFT optimizations were also performed at the SOGGA11-X/TZVP SMD/MeOH level but very similar results were found to those of the gas-phase and PCM calculations. The ECD calculations of these compounds emphasize further that it is always advisable to apply more than one DFT functional both for the DFT optimization and the TDDFT calculation steps [7,8,9]. The similar ECD spectra of **4** and **5** (the latter was noisy in the low-wavelength region) allow elucidation of the AC of **5** as (1*R*,2*S*,8*R*,11*R*). The absolute stereochemistry of the further compounds **6**–**9** was determined based on biosynthetic considerations.

**Figure 7:**
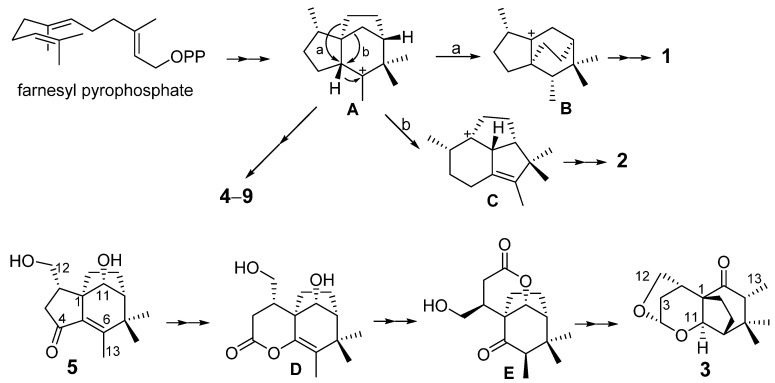
Plausible biosynthesis of sesquiterpenoids from agarwood.

To our knowledge, this is the first time that so many ziaane-type sesquiterpenes were identified from agarwood, which may be the characteristic chemicals of this agarwood. A plausible biosynthetic pathway of sesquiterpenoids from agarwood is proposed to start from a liner precursor farnesyl pyrophosphate (FPP) modified after literature (Figure 7) [13,14]. The FPP undergoes ionization, cyclization, hydride shift, spirocyclization, cyclization and *syn*-1,2-methyl migration to generate the intermediate cation **A**. The formation of zizaanes **4**–**9** has been proposed to involve A, *syn*-deprotonation and oxidations of intermediated cation C. In addition, intermediated cation A undergoes rearrangement reactions a or b leading to cations B or C, which were catalyzed to form novel compounds **1** or **2**, respectively. A biosynthetic pathway for **3** is proposed from **5** through 4,5-Bayer-Villiger oxidation, 4,5-hydrolysis and 5,6-enol-keto-tautomerization, 4,11-esterification and 4,12-condensation.

**Figure 8:**
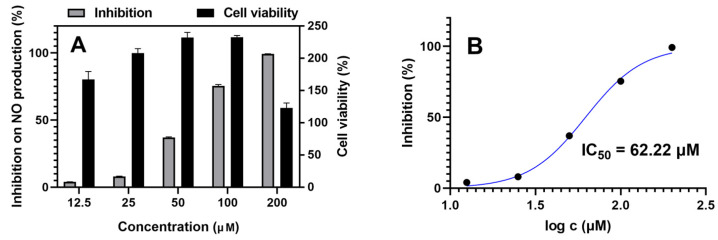
Anti-inflammatory activity assay using LPS-induced RAW264.7 cells. (**A**) Concentration effects of compound **9** on NO formation of LPS-induced RAW264.7 cells and on the viability of RAW264.7 cells, *n* = 3. (**B**) IC50 of compound **9** on inhibition of NO production in LPS-stimulated RAW264.7 cells, concentrations were transferred to Log(c).

All isolated sesquiterpenes (**1**–**9**) were tested for α-glucosidase inhibition and anti-inflammatory activities in vitro. Acarbose was used as a positive control for α-glucosidase inhibition with an IC_50_ value of 743.4 ± 3.3 μM. Quercetin and indomethacin were used as positive controls for anti-inflammatory activity with IC_50_ values of 8.22 ± 0.80 μM and 35.40 ± 1.77 μM, respectively. However, none of them exhibited α-glucosidase inhibition activity. Only compound **9** exhibited weak inhibition of NO production in LPS-stimulated RAW264.7 cells with an IC_50_ value of 62.22 ± 1.27 μM (Figure 8). The two hydroxy groups located at C-13 and C-15 effectively enhanced its anti-inflammatory activity. Although the anti-inflammatory activity of compound **9** is weaker than in the positive controls, this is the first report about the anti-inflammatory activity of ziaane-type sesquiterpenes. Further research is needed to confirm the activity and to uncover its exact mechanisms. In order to investigate whether the inhibitory activities of isolated sesquiterpenes were due to the decrease of cell numbers (cytotoxicity), their effects on cell viability also had been measured using the MTT method. None of them (up to 100 μM) showed cytotoxicity with LPS treatment.

## 3. Materials and Methods

### 3.1. General Procedures

Optical rotations were measured with a Rudolph Autopol I polarimeter (Rudolph, Hackettstown, NJ, USA). UV spectra were recorded on a Shimadzu UV-2550 spectrometer (Beckman, Kyoto, Japan). ECD spectra were measured on a JASCO J-715 spectrophotometer (JASCO, Tokyo, Japan). IR absorptions were obtained on a Nicolet 380 FT-IR instrument (Thermo, Pittsburgh, PA, USA) using KBr pellets. HRESIMS were determined by an API QSTAR Pulsar mass spectrometer (Bruker, Bremen, Germany) or 6200 series TOF/6500 series (Agilent, Palo Alto, CA, USA). The NMR spectra were recorded on Bruker Avance 500 NMR spectrometers (Bruker, Bremen, Germany), using TMS as an internal standard. HPLC purifications were performed on an analytical reversed-phase column (YMC-packed C_18_, 250 mm × 10 mm, 5 µm) (YMC, Tokyo, Japan) using a G1311C 1260 Quat Pump VL and detected with a G1315D 1260 DAD VL detector (190–500 nm) (Agilent Technologies 1260 infinity, Palo Alto, CA, USA). Column chromatography was performed with silica gel (60–80, 200–300 mesh, Qingdao Haiyang Chemical Co., Ltd., Qingdao, China), ODS gel (20–45 µm, Fuji Silysia Chemical Co., Ltd., Durham, NC, USA), and Sephadex LH-20 (Merck, Darmstadt, Germany). TLC was carried out on NUSCRIPT silica gel GF254 (Qingdao Haiyang Chemical Co., Ltd., China), and peaks were detected by spraying with 5% H_2_SO_4_ in EtOH followed by heating.

### 3.2. Plant Material

The plant material was collected in NANA International Agarwood Market of Thailand, in August of 2014, and identified as agarwood originated from *Aquilaria* sp. by Dr. Jun Wang (Institute of Tropical Bioscience and Biotechnology, Chinese Academy of Tropical Agricultural Sciences & Hainan engineering research center of agarwood). A voucher specimen (201408SLLK) has been deposited at the Institute of Tropical Bioscience and Biotechnology, Chinese Academy of Tropical Agricultural Sciences.

### 3.3. Extraction and Isolation

Air-dried of agarwood (384.0 g) was extracted with ethyl ether (1.5 L × 3). The extract was filtered and concentrated to get the ethyl ether extract (27.6 g). The ethyl ether extract (23.4 g) was applied to ODS gel CC eluting with MeOH–H_2_O (*v*/*v*, 2:3, to 1:0, 2 L of each) to provide 16 fractions (Fr.1–Fr.16). Fr.6 (0.6 g) was subjected to Sephadex LH-20 gel CC eluded with petroleum ether–CHCl_3_–MeOH (*v*/*v*/*v*, 2:1:1) to give six fractions (Fr.6-1–Fr.6-7). Fr.6-3 (140.4 mg) was separated on silica gel CC with petroleum ether–acetone (*v*/*v*, 150:1 to 0:1), then purified by silica gel CC using CHCl_3_–MeOH (*v*/*v*, 150:1) to afford compound **5** (2.3 mg). Fr.7 (1.3 g) was separated to Sephadex LH-20 gel CC eluded with CHCl_3_–MeOH (*v*/*v*, 1:1) and then fractionated by silica gel CC eluted with petroleum ether–EtOAc (*v*/*v*, 500:1 to 1:1, 1.0 L of each) to yield 13 fractions (Fr.7-1–Fr.7-13). Fr.7-7 and Fr.7-8 were merged together (547.7 mg) to be subjected on silica gel CC with petroleum ether–EtOAc–MeOH (*v*/*v*/*v*, 20:1:0.1 to 0:0:1) and silica gel CC using CHCl_3_–MeOH (*v*/*v*, 200:1), then purified by semi-preparative HPLC (C_18_ column; MeOH–H_2_O *v*/*v*, 55:45; flow rate 4.0 mL/min; UV detection at 200, 210 nm) to afford compounds **2** (32.2 mg) and **1** (1.2 mg). Fr.8 (799.1 g) was subjected to silica gel CC eluted with petroleum ether–EtOAc (*v*/*v*, 500:1 to 1:1, 1.0 L of each) to give 12 fractions (Fr.8-1–Fr.8-12). Fr.8-6 (36.8 mg) was separated on silica gel CC with petroleum ether–CHCl_3_–MeOH (*v*/*v*/*v*, 2:1:1), then purified by semi-preparative HPLC (C_18_ column; MeOH–H_2_O *v*/*v*, 65:35; flow rate 4.0 mL/min; UV detection at 200, 220 nm) to afford compound **8** (4.4 mg). Fr.8-11 (48.0 mg) was applied to silica gel CC with petroleum ether–EtOAc (*v*/*v*, 30:1), then purified by semi-preparative HPLC (C_18_ column; MeOH–H_2_O *v*/*v*, 65:35; flow rate 4.0 mL/min; UV detection at 200, 220 nm) to afford compound **7** (8.3 mg). Fr.9 (1.8 g) was divided to Sephadex LH-20 gel CC eluting with petroleum ether–CHCl_3_–MeOH (*v*/*v*/*v*, 2:1:1) to obtain 11 fractions (Fr.9-1–Fr.9-11). Fr.9-3 (472.4 mg) was separated on silica gel CC with petroleum ether–EtOAc (*v*/*v*, 500:1 to 1:1, 1.0 L of each) and recrystallization to get compound **4** (30.5 mg). Fr.10 (2.0 g) was chromatographed on Sephadex LH-20 gel CC eluting with petroleum ether–CHCl_3_–MeOH (*v*/*v*/*v*, 2:1:1) to yield Fr.10-1–Fr.10-5. Fr.10-2 (944.8 mg) was subjected to silica gel CC with CHCl_3_–MeOH (*v*/*v*, 300:1 to 0:1, 1.0 L of each) and silica gel CC with petroleum ether–EtOAc (*v*/*v*, 25:1 to 1:1), then further purified by HPLC (C_18_ column, MeOH–H_2_O *v*/*v*, 70:30; flow rate 4.0 mL/min; UV detection at 200, 240 nm) to get compound **6** (15.0 mg), **9** (12.0 mg), **3** (36.4 mg).

(5*R*,6*S*,8*R*,11*R*)-10(1→5)*abeo*-11-hydroxy-ziza-1(2)-en-3-one (**1**): colorless bulk crystals (MeOH); ${\left[\alpha \right]}_{D}^{25}$ +98 (*c* 0.11, MeOH); UV (MeOH) *λ*_max_: 246 nm; ECD (6.65 × 10^−4^ M, MeOH) λ_max_ (Δε): 315 (+1.39), 251 (+2.05), 216 (−5.69) nm; IR (KBr) *ν*_max_: 3441, 2964, 2914, 2875, 1682, 1650, 1442, 1406, 1287, 1093, 1050, 961 cm^−1^; ^1^H and ^13^C NMR data see Table 1 and Table 2; HRESIMS *m*/*z* 257.1513 [M + Na]^+^ (calcd. for C_15_H_22_NaO_2_ 257.1512).

(1S*,2R*,8S*,11R*)-11(1→5)*abeo*-ziza-5(6)-en-1,12-diol (**2**): colorless bulk crystals (MeOH); ${\left[\alpha \right]}_{D}^{25}$ +35 (*c* 0.12, MeOH); UV (MeOH) *λ*_max_: 216 nm. IR (KBr) *ν*_max_: 3425, 2956, 1617, 1450, 1397, 1090, 1024, 803 cm^−1^; ^1^H and ^13^C NMR data see Table 1 and Table 2; HRESIMS *m*/*z*: 259.1672 [M + Na]^+^ (calcd. for C_15_H_24_NaO_2_, 259.1669).

(1*S*,2*S*,4*S*,6*R*,8*R*,11*R*)-agarozizone (**3**): colorless oil; ${\left[\alpha \right]}_{D}^{25}$ +24 (*c* 0.05, CH_3_OH); UV (MeOH) *λ*_max_: 204 nm. ECD (7.99 × 10^−4^ M, MeOH) λ_max_ (Δε): 296 (−2.00), 207 (−0.29) nm; IR (KBr) *ν*_max_: 2954, 2924, 1701, 1635, 1399, 1185, 1104, 1027, 799 cm^−1^; ^1^H and ^13^C NMR data see Table 1 and Table 2; HRESIMS *m*/*z*: 273.1471 [M + Na]^+^ (calcd. for C_15_H_22_NaO_3_, 273.1461).

(1*R*,2*S*,8*S*)-12-hydroxy-ziza-5(6)-en-4-one (**4**): colorless bulk crystals (MeOH); ${\left[\alpha \right]}_{D}^{25}$ +101 (*c* 0.16, CH_3_OH); UV (MeOH) *λ*_max_: 261 nm; ECD (8.53 × 10^−4^ M, MeOH) λ_max_ (Δε): 348 (+1.37), 260 (−3.15), 214sh (+4.51) nm; IR (KBr) *ν*_max_: 3423, 2960, 1701, 1615, 1406, 1202, 1030, 794 cm^−1^; ^1^H and ^13^C NMR data see Table 3 and Table 4; HRESIMS *m*/*z* 257.1512 [M + Na]^+^ (calcd. for C_15_H_22_NaO_2_ 257.1512).

(1*R*,2*S*,8*R*,11*R*)-11,12-dihydroxy-ziza-5(6)-en-4-one (**5**): colorless oil; ${\left[\alpha \right]}_{D}^{25}$ +83 (*c* 0.04, CH_3_OH). UV (MeOH) *λ*_max_: 259 nm; ECD (7.99 × 10^−4^ M, MeOH) λ_max_ (Δε): 349 (+1.67), 234 (−0.66) nm; IR (KBr) *ν*_max_: 3414, 3210, 2957, 1626, 1398, 1099, 1029, 800 cm^−1^; ^1^H and ^13^C NMR data see Table 3 and Table 4; HRESIMS *m*/*z* 273.1459 [M + Na]^+^ (calcd. for C_15_H_22_NaO_3_ 273.1461).

(1*R*,2*S*,8*R*,11*R*)-ziza-5(6)-en-11,12-diol (**6**): white amorphous powder; ${\left[\alpha \right]}_{D}^{25}$ –12 (*c* 0.27, CH_3_OH); UV (MeOH) *λ*_max_: 205 nm; IR (KBr) *ν*_max_: 3203, 2938, 1638, 1400, 1033, 794 cm^−1^; ^1^H and ^13^C NMR data see Table 3 and Table 4; HRESIMS *m*/*z* 259.1674 [M + Na]^+^ (calcd. for C_15_H_24_NaO_2_ 259.1669).

(1*R*,2*S*,4*S*,8*S*)-4-methoxy-ziza-5(6)-en-12-ol (**7**): colorless oil; ${\left[\alpha \right]}_{D}^{25}$ +40 (*c* 0.18, CH_3_OH); UV (MeOH) *λ*_max_: 210, 260 nm; IR (KBr) *ν*_max_: 3421, 2957, 1702, 1617, 1453, 1373, 1200, 1071, 1028, 805 cm^−1^; ^1^H and ^13^C NMR data see Table 3 and Table 4; HRESIMS *m*/*z* 273.1817 [M+Na]^+^ (calcd. for C_16_H_26_NaO_2_ 273.1825).

(1*R*,2*S*,4*R*,8*S*)-4-methoxy-ziza-5(6)-en-12-ol (**8**): colorless oil; ${\left[\alpha \right]}_{D}^{25}$ +18 (*c* 0.11, CH_3_OH); UV (MeOH) *λ*_max_: 209, 260 nm; IR (KBr) *ν*_max_: 3411, 2955, 1615, 1394, 1087, 1028, 800 cm^−1^; ^1^H and ^13^C NMR data see Table 3 and Table 4; HRESIMS *m*/*z* 273.1829 [M + Na]^+^ (calcd. for C_16_H_26_NaO_2_ 273.1825).

(1*R*,2*S*,7*S*,8*S*)-ziza-5(6)-en-13,15-diol (**9**): colorless oil; ${\left[\alpha \right]}_{D}^{25}$ –29 (*c* 0.15, CH_3_OH); UV (MeOH) *λ*_max_: 215 nm; IR (KBr) *ν*_max_: 3375, 2948, 1641, 1458, 1400, 1027, 799 cm^−1^; ^1^H and ^13^C NMR data see Table 3 and Table 4; HRESIMS *m*/*z* 259.1669 [M + Na]^+^ (calcd. for C_15_H_24_NaO_2_ 259.1669).

### 3.4. Computational Methods

Mixed torsional/low-mode conformational searches were carried out by means of the Macromodel 10.8.011 software using the MMFF with an implicit solvent model for CHCl_3_ applying a 21 kJ/mol energy window [15]. Geometry reoptimizations of the resultant conformers [B3LYP/6-31+G(d,p) level in vacuo, CAM-B3LYP/TZVP with PCM solvent model for MeOH and SOGGA11-X/TZVP SMD/MeOH] and TDDFT ECD calculations were performed with Gaussian 09 using various functionals (B3LYP, BH&HLYP, CAM-B3LYP, PBE0) and the TZVP basis set with the same or no solvent model as in the preceding DFT optimization step [16]. ECD spectra were generated as the sum of Gaussians with 2400 and 3000 cm^−1^ half-height widths, using dipole-velocity-computed rotational strengths [17]. Boltzmann distributions were estimated from the B3LYP, CAM-B3LYP and SOGGA11-X energies. The MOLEKEL program was used for visualization of the results [18].

### 3.5. Bioactivity Assays

#### 3.5.1. α-Glucosidase Inhibitory Activity Assay

The α-glucosidase inhibitory activity of all isolated compounds was evaluated by the PNPG method in vitro as described previously [3,4]. Acarbose was used as a positive control with an IC_50_ value of 743.4 ± 3.3 μM.

#### 3.5.2. Anti-Inflammatory Assay

All compounds were evaluated for their inhibitory effects on NO production in LPS-stimulated mouse mononuclear macrophages (RAW264.7) macrophages using the Griess assay as described before [19]. The RAW264.7 cells were purchased from the Stem Cell Bank of the Chinese Academy of Sciences. Each compound was diluted in half by concentration gradients (200 µM, 100 µM, 50 µM, 25 µM, 12.5 µM). Quercetin and indomethacin were used as positive controls with IC_50_ values of 8.22 ± 0.80 μM and 35.40 ± 1.77 μM, respectively. The effects on cell viability of isolated sesquiterpenes also had been measured using the MTT method.

## 4. Conclusions

Three novel zizaane derivatives (**1**–**3**), together with six zizaane-type sesquiterpenoids (**4**–**9**) were identified from ethyl ether extract of agarwood originated from *Aquilaria* sp., which could be the characteristic chemicals of this kind of agarwood. Their structures were unambiguously elucidated on the basis of HRESIMS data, 1D and 2D NMR and comparison of the experimental and computed ECD spectra. In vitro anti-inflammatory assessment showed that only compound **9** exhibited weak anti-inflammatory activity with an IC_50_ value of 62.22 ± 1.27 μM.

## Figures and Tables

**Table 1:** ^1^H NMR spectroscopic data of compounds **1**–**3** (500 MHz, *δ* in ppm).

No.	1 ^a^	2 ^b^	3 ^b^
2		1.98, m	2.68, t (4.3)
3		1.88, m	2.25, d (12.1)
		0.90, dddd (13.1, 13.0, 13.0, 4.2)	1.59, ddd (12.1, 4.6, 3.1)
4	2.14, d (18.6)	2.43, ddd (13.6, 4.1, 2.3)	5.30, d (3.1)
	2.02, d (18.6)	1.74, m	
6	1.22, m		2.51, q (6.7)
8	1.51, ddd (3.0, 3.0, 3.0)	2.37, ddd (10.4, 6.6, 2.6)	1.94, d (6.2)
9	1.80, m	1.86, m	2.21, m
	1.75, m	1.75, m	2.16, m
10	1.98, m	1.46, m	2.14, m
	1.13, m	1.27, dd (12.9, 7.9)	1.63, m
11	4.99, brs	2.70, brd (6.3)	4.08, s
12	1.84, brs	3.82, dd (10.5, 6.1)	4.02, d (8.4)
		3.40, dd (10.5, 7.6)	3.90, dd (8.4, 3.9)
13	0.92, d (7.3)	1.47, brs	0.92, d (6.8)
14	0.99, s	0.96, s	1.13, s
15	1.01, s	1.01, s	0.85, s

^a^ measured in CDCl_3_; ^b^ measured in MeOH-*d*_4_.

**Table 2:** ^13^C NMR spectroscopic data of compounds **1**–**3** (125 MHz, *δ* in ppm).

No.	1 ^a^	2 ^b^	3 ^b^
1	179.3, C	85.4, C	60.2, C
2	134.4, C	48.4, CH	35.9, CH
3	209.4, C	27.9, CH_2_	34.7, CH_2_
4	45.3, CH_2_	26.1, CH_2_	101.0, CH
5	44.7, C	133.6, C	214.6, C
6	43.9, CH	138.4, C	48.9, CH
7	34.2 C	49.5, C	39.0, C
8	46.4, CH	51.4, CH	54.5, CH
9	16.3, CH_2_	26.0, CH_2_	25.9, CH_2_
10	25.0, CH_2_	31.4, CH_2_	30.6, CH_2_
11	66.5, CH	62.5, CH	78.7, CH
12	8.3, CH_3_	65.2, CH_2_	73.8, CH_2_
13	11.5, CH_3_	9.4, CH_3_	8.7, CH_3_
14	30.2, CH_3_	29.5, CH_3_	27.5, CH_3_
15	25.0, CH_3_	23.2, CH_3_	23.7, CH_3_

^a^ measured in CDCl_3_; ^b^ measured in MeOH-*d*_4_.

**Table 3:** ^1^H NMR spectroscopic data of compounds **4**–**9** (500 MHz, *δ* in ppm).

No.	4 ^a^	5 ^a^	6 ^a^	7 ^b^	8 ^b^	9 ^a^
2	2.32, m	2.44, m	2.12, m	2.25, m	2.02, m	1.79, m
3	2.40, dd (17.2, 7.6)	2.32, dd (17.8, 7.8)	1.75, m	2.09, dd (13.4, 5.9)	2.31, dt (12.7, 7.2)	1.83, m
	2.10, dd (17.2, 12.4)	2.04, m	1.30, m	1.34, m	1.48, m	1.30, m
4			2.31, dd (17.1, 9.4)	4.12, d (5.7)	4.24, t (6.4)	2.51, dd (17.4, 8.8)
			2.16, m			2.28, m
8	1.90, m	1.83, d (7.6)	1.77, m	1.84, dd (6.9, 5.6)	1.82, m	2.08, dd (6.5, 5.8)
9	1.82, m	1.93, m	1.81, m	1.38, m	1.82, m	1.99, m
	1.77, m	1.78, m	1.74, m	1.25, m	1.66, m	1.65, m
10	1.65, td (11.3, 6.5)	2.00, m	1.81, m	1.75, m	1.56, m	1.46, td (11.7, 5.3)
	1.46, m	1.46, m	1.29, m	1.62, m	1.49, m	1.28, m
11	1.88, dd (10.3, 5.4)	3.89, s	3.77, s	1.70, d (10.5)	1.60, dd (11.6, 6.4)	1.60, dd (10.6, 5.4)
	1.72, brd (10.3)			1.63, m	1.54, d (11.3)	1.41, d (10.6)
12	3.75, dd (10.8, 6.3)	3.68, dd (10.7, 4.6)	3.63, dd (10.7, 4.8)	3.76, dd (10.6, 6.2)	3.76, dd (10.5, 5.7)	0.97, d (6.5)
	3.63, dd (10.8, 7.2)	3.55, t (10.3)	3.45, t (10.6)	3.62, dd (10.6, 7.6)	3.66, dd (10.5, 6.6)	
13	2.05, s	2.06, s	1.47, t (1.4)	1.59, s	1.58, s	4.01, d (11.6)
						3.95, d (11.6)
14	1.16, s	1.23, s	1.09, s	1.06, s	1.04, s	1.08, s
15	1.12, s	1.13, s	1.01, s	1.00, s	1.02, s	3.66, d (11.3)
						3.62, d (11.3)
4-OCH_3_				3.30, s	3.27, s	

^a^ measured in MeOH-*d*_4_; ^b^ measured in CDCl_3_.

**Table 4:** ^13^C NMR spectroscopic data of compounds **4**–**9** (125 MHz, *δ* in ppm).

No.	4 ^a^	5 ^a^	6 ^a^	7 ^b^	8 ^b^	9 ^a^
1	52.0, C	57.3, C	58.5, C	51.5, C	50.9, C	54.4, C
2	42.1, CH	41.4, CH	48.9, CH	45.0, CH	43.9, CH	40.8, CH
3	43.8, CH_2_	42.4, CH_2_	27.8, CH_2_	33.6, CH_2_	35.1, CH_2_	33.4, CH_2_
4	208.7, C	207.2, C	28.2, CH_2_	79.7, CH	80.0, CH	27.7, CH_2_
5	140.1, C	138.5, C	142.7, C	143.5, C	141.9, C	153.9, C
6	155.8, C	156.4, C	130.5, C	137.1, C	135.9, C	130.2, C
7	43.9, C	45.5, C	43.4, C	41.1, C	41.0, C	46.4, C
8	47.4, CH	54.2, CH	55.1, CH	47.1, CH	46.8, CH	44.6, CH
9	25.5, CH_2_	23.1, CH_2_	22.9, CH_2_	30.6, CH_2_	24.6, CH_2_	25.1, CH_2_
10	31.2, CH_2_	27.7, CH_2_	26.8, CH_2_	24.4, CH_2_	31.2, CH_2_	29.2, CH_2_
11	39.4, CH_2_	79.1, CH	79.6, CH	38.2, CH_2_	39.2, CH_2_	37.9, CH_2_
12	63.9, CH_2_	62.4, CH_2_	62.7, CH_2_	64.4, CH_2_	64.4, CH_2_	14.2, CH_3_
13	13.6, CH_3_	13.4, CH_3_	13.2, CH_3_	13.4, CH_3_	12.8, CH_3_	59.4, CH_2_
14	28.7, CH_3_	29.1, CH_3_	29.2, CH_3_	29.0, CH_3_	28.4, CH_3_	24.3, CH_3_
15	24.8, CH_3_	25.1, CH_3_	25.7, CH_3_	25.0, CH_3_	25.3, CH_3_	69.0, CH_2_
4-OCH_3_				56.3, OCH_3_	55.6, OCH_3_	

^a^ measured in MeOH-*d*_4_; ^b^ measured in CDCl_3_.

## Supplementary Materials

The following supporting information can be downloaded. HRESIMS and NMR spectra for compounds **1**–**9,** and low-energy conformers of compounds **1**, **3** and **4** are available online.

## Sample Availability

Not available.
