# Supplementary material for: Zizaane-Type Sesquiterpenoids and Their Rearranged Derivatives from Agarwood of an Aquilaria Plant
Source: Molecules. 2021 Dec 29;27(1):198. doi: 10.3390/molecules27010198 (PMC8746309; doi:10.3390/molecules27010198)
Supplement: Supplementary file 1 [file molecules-27-00198-s001.zip › molecules-1515956-supplementary.pdf]

Supporting Information

Zizaane-Type Sesquiterpenoids and Their Rearranged Derivatives from Agarwood of an *Aquilaria* Plant

Jing-Zhe Yuan [1,2], Yi-Ling Yang [2], Wei Li [2], Li Yang [2], Hao-Fu Dai [2], Attila Mándi [3], Cai-Hong Cai [2], Hui-Qin Chen [2], Wen-Hua Dong [2], Tibor Kurtán [3], Wen-Li Mei [2,*] and Hao Wang [2,*]

[1] School of Life Sciences, Hainan University, Haikou 570228, China; yuanjingzhenpc@126.com

[2] Hainan Engineering Research Center of Agarwood, Institute of Tropical Bioscience and Biotechnology, Chinese Academy of Tropical Agricultural Sciences, Haikou 571101, China; yangyil@staff.uni-marburg.de (Y.-L.Y.); liwei@itbb.org.cn (W.L.); yangli@itbb.org.cn (L.Y.); daihaofu@itbb.org.cn (H.-F.D.); caicaihong@itbb.org.cn (C.-H.C.); chenhuiqin@itbb.org.cn (H.-Q.C.); dongwenhua@itbb.org.cn (W.-H.D.)

[3] Department of Organic Chemistry, University of Debrecen, P.O. Box 400, H-4002 Debrecen, Hungary; mandi.attila@science.unideb.hu (A.M.); kurtan.tibor@science.unideb.hu (T.K.)

* Correspondence: meiwenli@itbb.org.cn (W.-L.M.); wanghao@itbb.org.cn (H.W.); Tel./Fax: +86-898-66987529 (W.-L.M.); +86-898-66988061 (H.W.)

TABLE OF CONTENTS

Supporting Information .... **I**
TABLE OF CONTENTS II
Figure S1. HRESIMS spectrum of **1** 1
Figure S2. ^{1}H NMR spectrum of compound **1** in $CDCl_3$ (500 MHz) 1
Figure S3. ^{13}C NMR spectrum of compound **1** in $CDCl_3$ (125 MHz) 2
Figure S4. HSQC spectrum of compound **1** 2
Figure S5. HMBC spectrum of compound **1** 3
Figure S6. ^{1}H-^{1}H COSY spectrum of compound **1** 3
Figure S7. ROESY spectrum of compound **1** 4
Figure S8. HRESIMS spectrum of **2** 4
Figure S9. ^{1}H NMR spectrum of compound **2** in MeOH-d_4 (500 MHz) 5
Figure S10. ^{13}C NMR spectrum of compound **2** in MeOH-d_4 (125 MHz) 5
Figure S11. HSQC spectrum of compound **2** 6
Figure S12. HMBC spectrum of compound **2** 6
Figure S13. ^{1}H-^{1}H COSY spectrum of compound **2** 7
Figure S14. ROESY spectrum of compound **2** 7
Figure S15. HRESIMS spectrum of **3** 8
Figure S16. ^{1}H NMR spectrum of compound **3** in MeOH-d_4 (500 MHz) 8
Figure S17. ^{13}C NMR spectrum of compound **3** in MeOH-d_4 (125 MHz) 9
Figure S18. HSQC spectrum of compound **3** 9
Figure S19. HMBC spectrum of compound **3** 10
Figure S20. ^{1}H-^{1}H COSY spectrum of compound **3** 10
Figure S21. ROESY spectrum of compound **3** 11
Figure S22. HRESIMS spectrum of **4** 11
Figure S23. ^{1}H NMR spectrum of compound **4** in MeOH-d_4 (500 MHz) 12
Figure S24. ^{13}C NMR spectrum of compound **4** in MeOH-d_4 (125 MHz) 12
Figure S25. HSQC spectrum of compound **4** 13
Figure S26. HMBC spectrum of compound **4** 13
Figure S27. ^{1}H-^{1}H COSY spectrum of compound **4** 14
Figure S28. ROESY spectrum of compound **4** 14
Figure S29. HRESIMS spectrum of **5** 15
Figure S30. ^{1}H NMR spectrum of compound **5** in MeOH-d_4 (500 MHz) 15
Figure S31. ^{13}C NMR spectrum of compound **5** in MeOH-d_4 (125 MHz) 16
Figure S32. HSQC spectrum of compound **5** 16
Figure S33. HMBC spectrum of compound **5** 17
Figure S34. ^{1}H-^{1}H COSY spectrum of compound **5** 17
Figure S35. ROESY spectrum of compound 5 18
Figure S36. HRESIMS spectrum of **6** 18
Figure S37. ^{1}H NMR spectrum of compound **6** in MeOH-d_4 (500 MHz) 19
Figure S38. ^{13}C NMR spectrum of compound **6** in MeOH-d_4 (125 MHz) 19
Figure S39. HSQC spectrum of compound **6** 20
Figure S40. HMBC spectrum of compound **6** 20

Figure S41. ^{1}H-^{1}H COSY spectrum of compound **6** 21
Figure S42. ROESY spectrum of compound **6**........ 21
Figure S43. HRESIMS spectrum of **7** 22
Figure S44. ^{1}H NMR spectrum of compound **7** in $CDCl_3$ (500 MHz)........ 22
Figure S45. ^{13}C NMR spectrum of compound **7** in $CDCl_3$ (125 MHz)........ 23
Figure S46. HSQC spectrum of compound **7**........ 23
Figure S47. HMBC spectrum of compound **7**........ 24
Figure S48. ^{1}H-^{1}H COSY spectrum of compound **7** 24
Figure S49. ROESY spectrum of compound **7**........ 25
Figure S50. HRESIMS spectrum of **8** 25
Figure S51. ^{1}H NMR spectrum of compound **8** in $CDCl_3$ (500 MHz)........ 26
Figure S52. ^{13}C NMR spectrum of compound **8** in $CDCl_3$ (125 MHz)........ 26
Figure S53. HSQC spectrum of compound **8**........ 27
Figure S54. HMBC spectrum of compound **8**........ 27
Figure S55. ^{1}H-^{1}H COSY spectrum of compound **8** 28
Figure S56. ROESY spectrum of compound **8**........ 28
Figure S57. HRESIMS spectrum of **9** 29
Figure S58. ^{1}H NMR spectrum of compound **9** in MeOH-d_4 (500 MHz)........ 29
Figure S59. ^{13}C NMR spectrum of compound **9** in MeOH-d_4 (125 MHz)........ 30
Figure S60. HSQC spectrum of compound **9**........ 30
Figure S61. HMBC spectrum of compound **9**........ 31
Figure S62. ^{1}H-^{1}H COSY spectrum of compound **9** 31
Figure S63. ROESY spectrum of compound **9**........ 32
Figure S64. Single low-energy conformer of (1*S*,2*S*,4*S*,6*R*,8*R*,11*R*)-**3Error! Bookmark not defined.**
Figure S65. Low-energy conformers and populations of (5*R*,6*S*,8*R*,11*R*)-**1Error! Bookmark not defined.**
Figure S66. Low-energy conformers and populations of (1*R*,2*S*,8*S*)-**4Error! Bookmark not defined.**

User Spectra

Fragmentor Voltage	Collision Energy	Ionization Mode
135	0	ESI

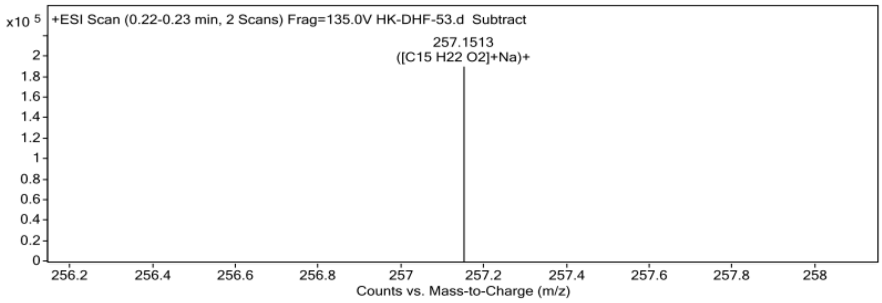

Peak List

m/z	z	Abund	Formula	Ion
235.1692	1	122726.66		
236.1726	1	21385.66		
257.1513	1	190521.78	C15 H22 O2	(M+Na)+
258.1544	1	28122.06	C15 H22 O2	(M+Na)+
273.1251	1	20204.07		
317.1836	1	12844.53		
491.3138	1	105373.56		
492.3171	1	32860.65		

Formula Calculator Element Limits

Element	Min	Max
C	3	60
H	0	120
O	0	30

Formula Calculator Results

Formula	CalculatedMass	CalculatedMz	Mz	Diff. (mDa)	Diff. (ppm)	DBE
C15 H22 O2	234.1620	257.1512	257.1513	-0.10	-0.39	5.0000

--- End Of Report ---

Figure S1. HRESIMS spectrum of **1**

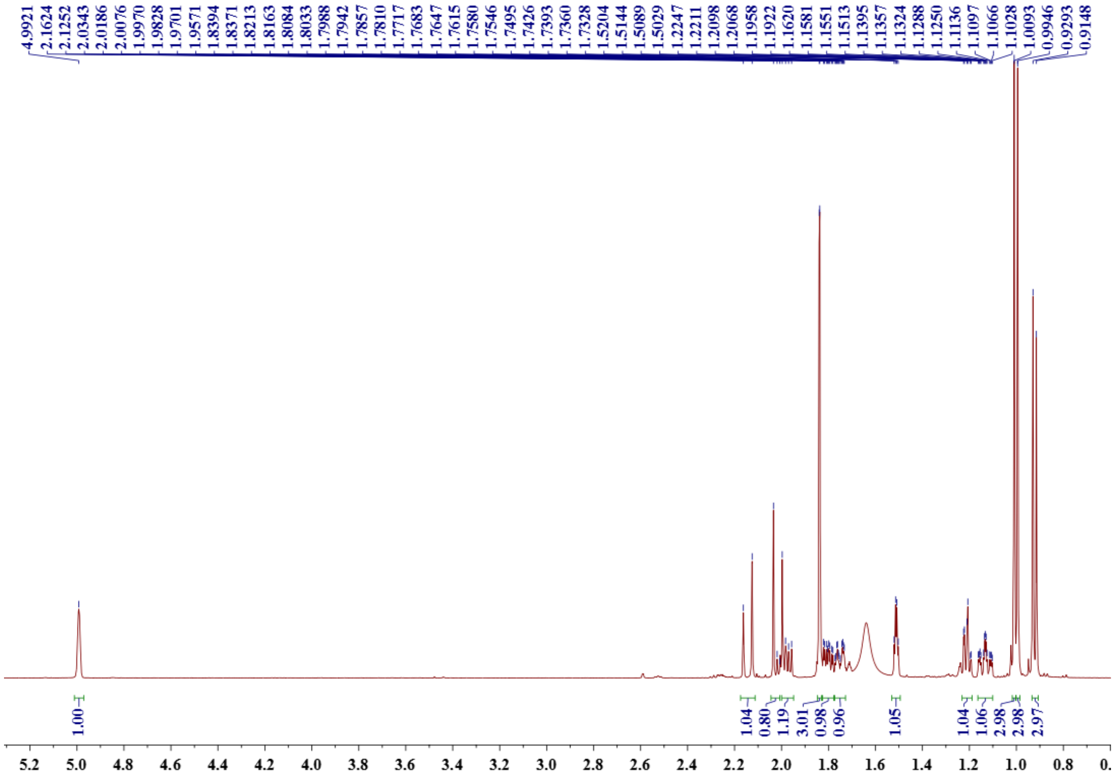

Figure S2. ^{1}H NMR spectrum of compound **1** in $CDCl_3$ (500 MHz)

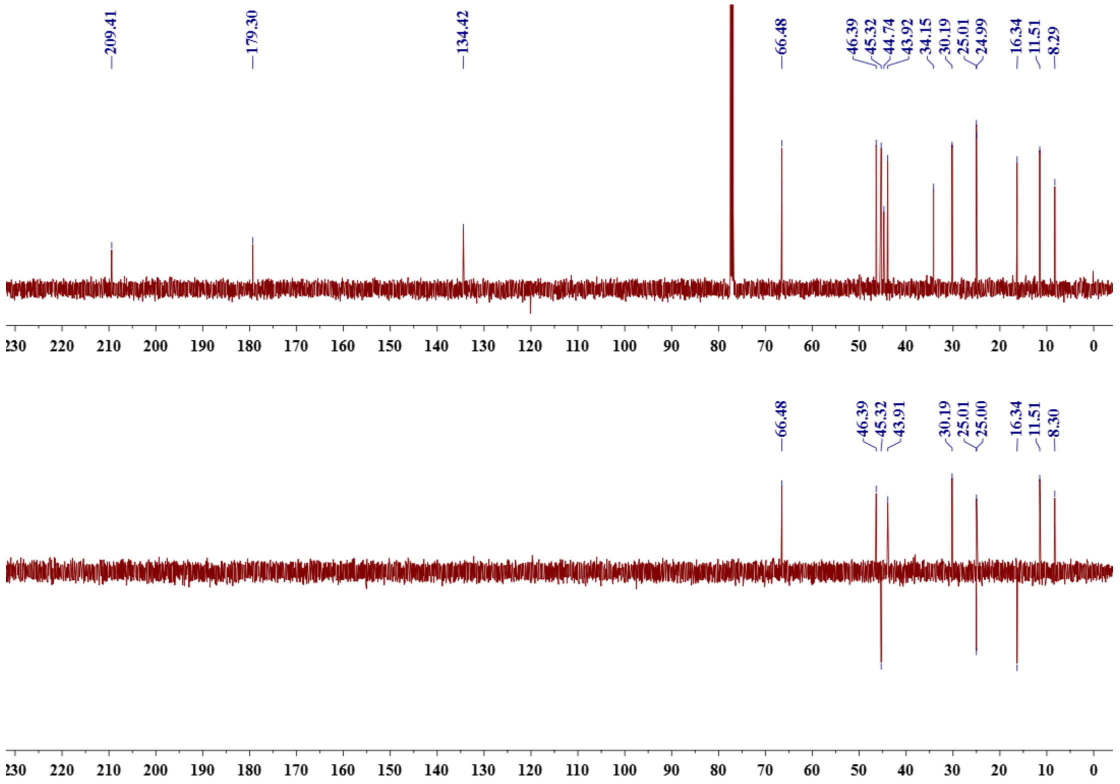

Figure S3. ^{13}C NMR spectrum of compound **1** in $CDCl_3$ (125 MHz)

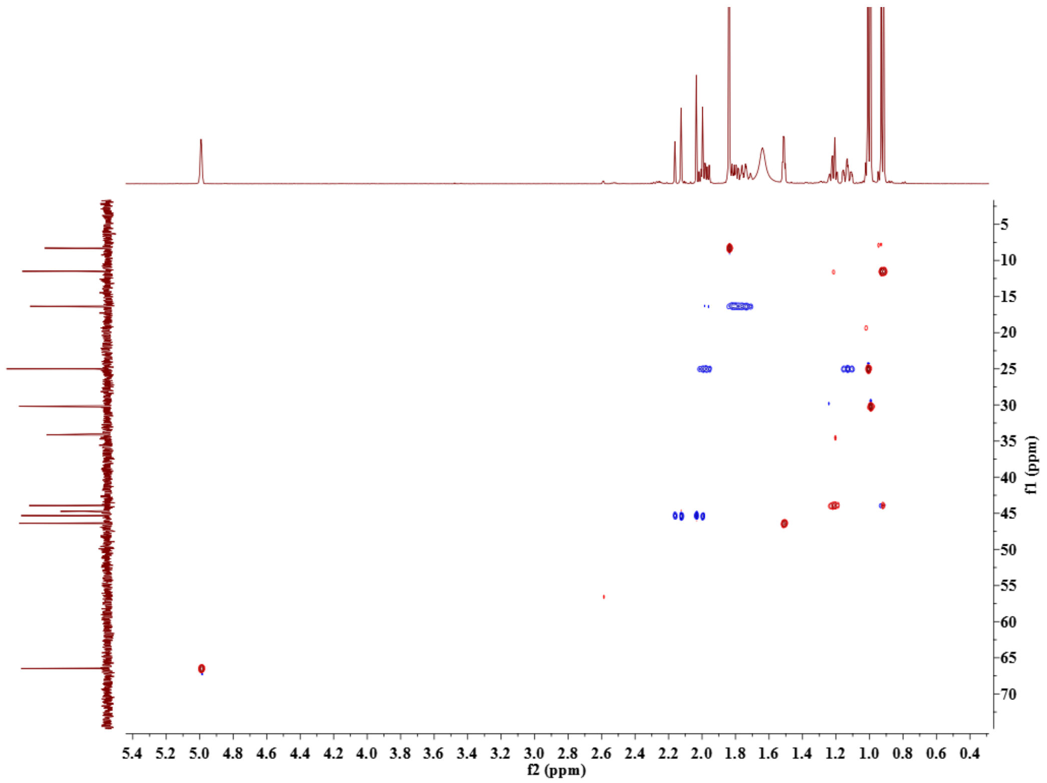

Figure S4. HSQC spectrum of compound **1**

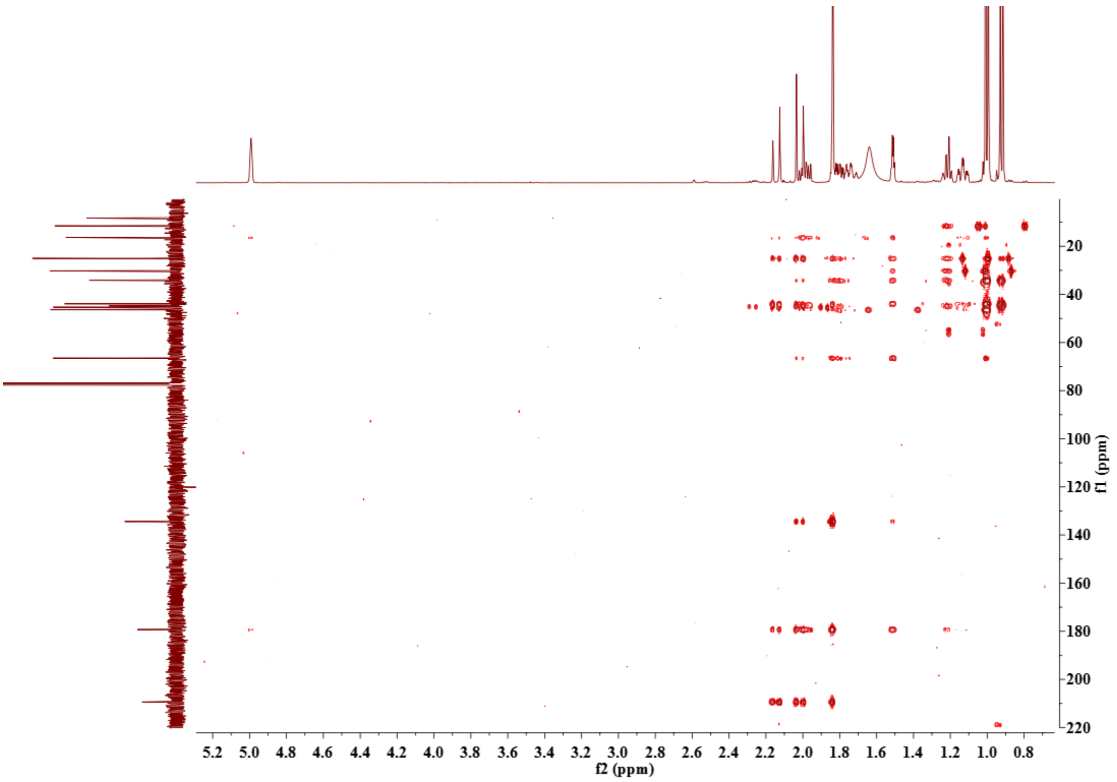

Figure S5. HMBC spectrum of compound **1**

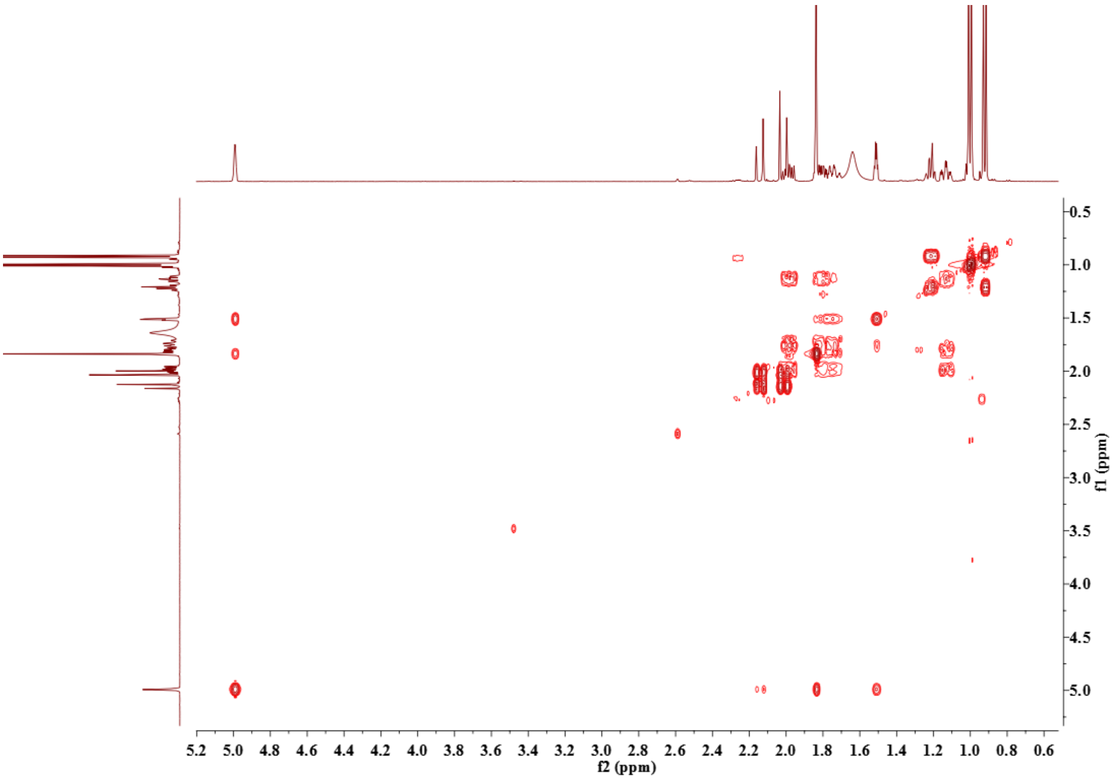

Figure S6. ^{1}H-^{1}H COSY spectrum of compound **1**

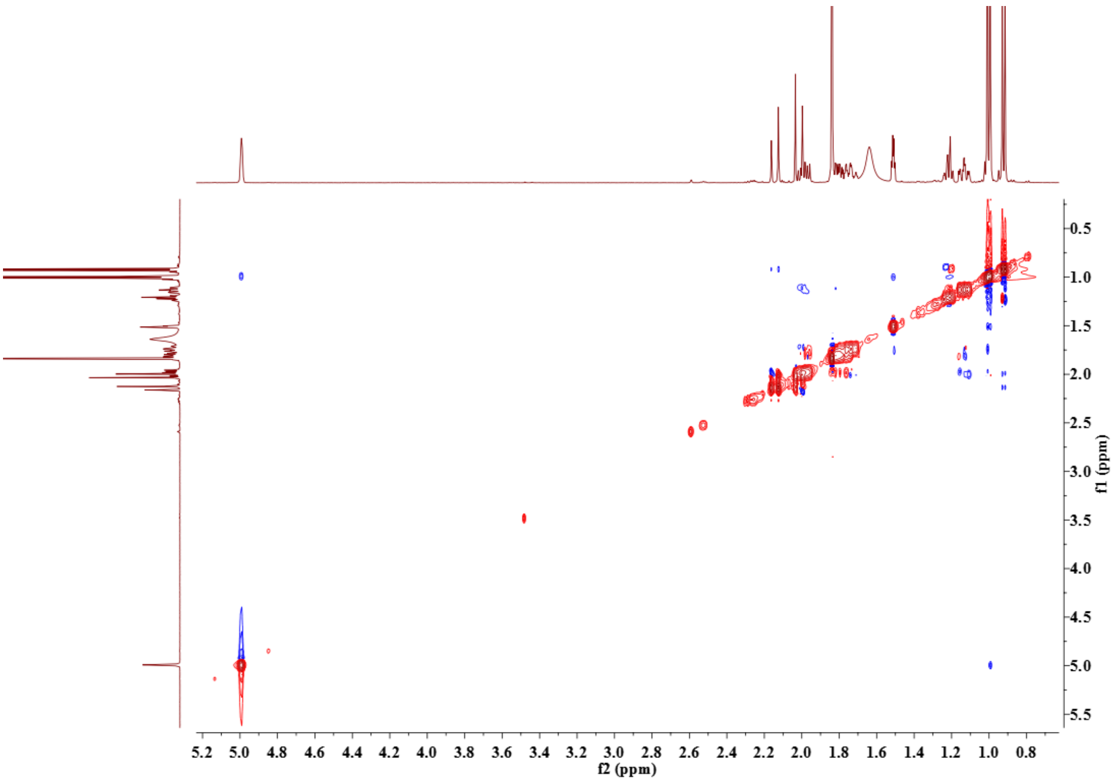

Figure S7. ROESY spectrum of compound **1**

Acquisition Parameter

Source Type	ESI	Ion Polarity	Positive	Set Nebulizer	0.4 Bar
Focus	Not active	Set Capillary	4500 V	Set Dry Heater	180 °C
Scan Begin	50 m/z	Set End Plate Offset	-500 V	Set Dry Gas	4.0 l/min
Scan End	1300 m/z	Set Charging Voltage	2000 V	Set Divert Valve	Source
		Set Corona	0 nA	Set APCI Heater	0 °C

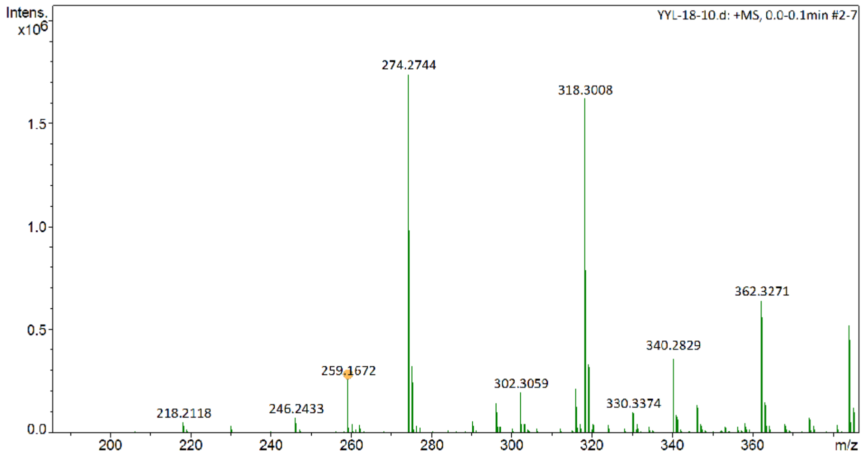

Sum Formula	Ion Formula	Meas. m/z	m/z	err [mDa]	err [ppm]
C15H24O2	C15H24NaO2	259.1672	259.1669	-0.4	-1.5

Figure S8. HRESIMS spectrum of **2**

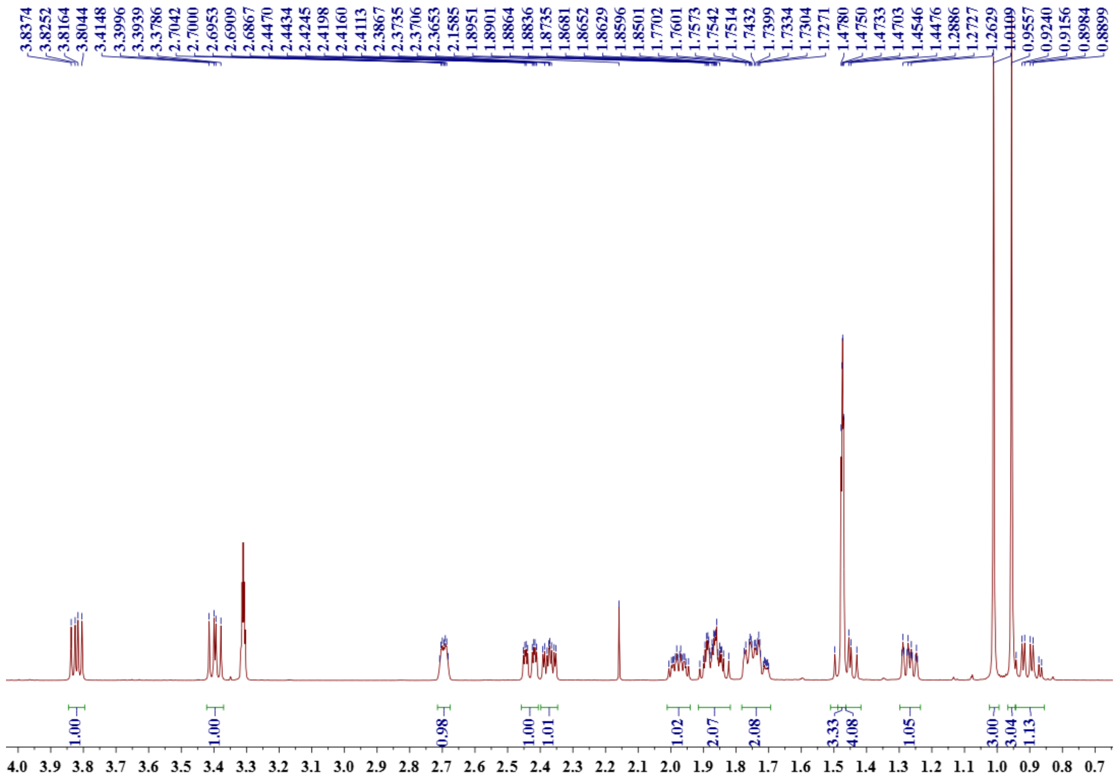

Figure S9. ^{1}H NMR spectrum of compound **2** in MeOH-d_4 (500 MHz)

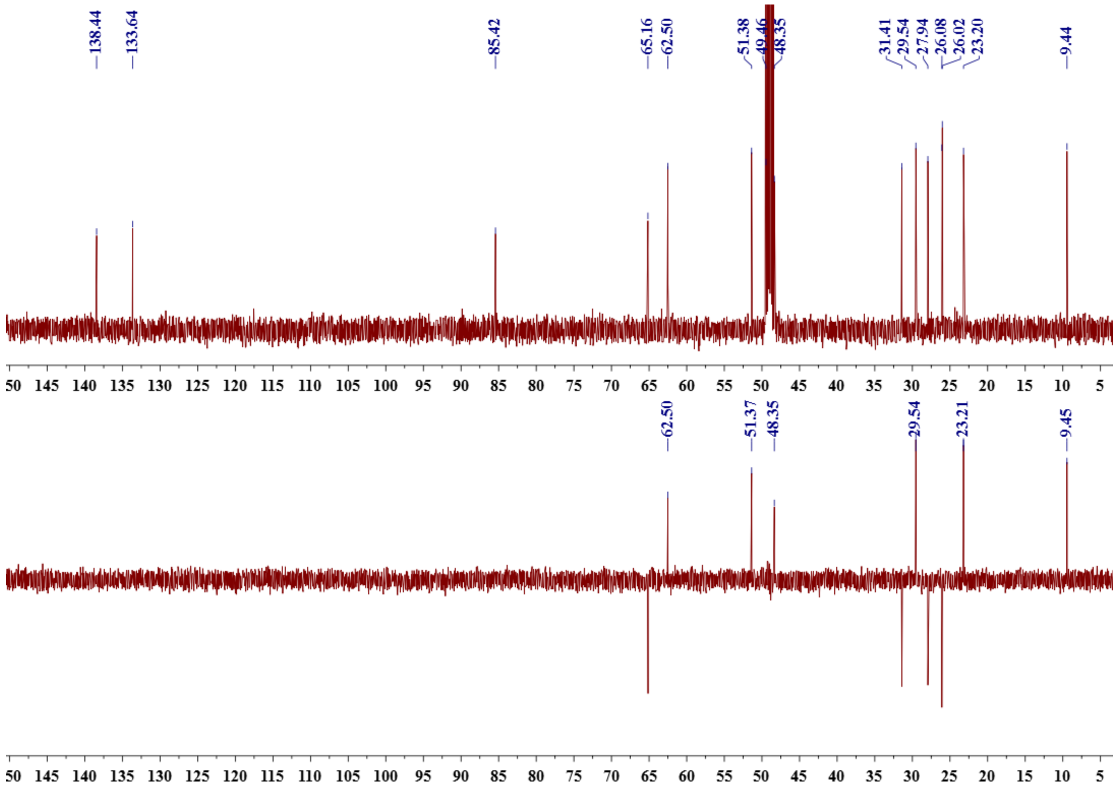

Figure S10. ^{13}C NMR spectrum of compound **2** in MeOH-d_4 (125 MHz)

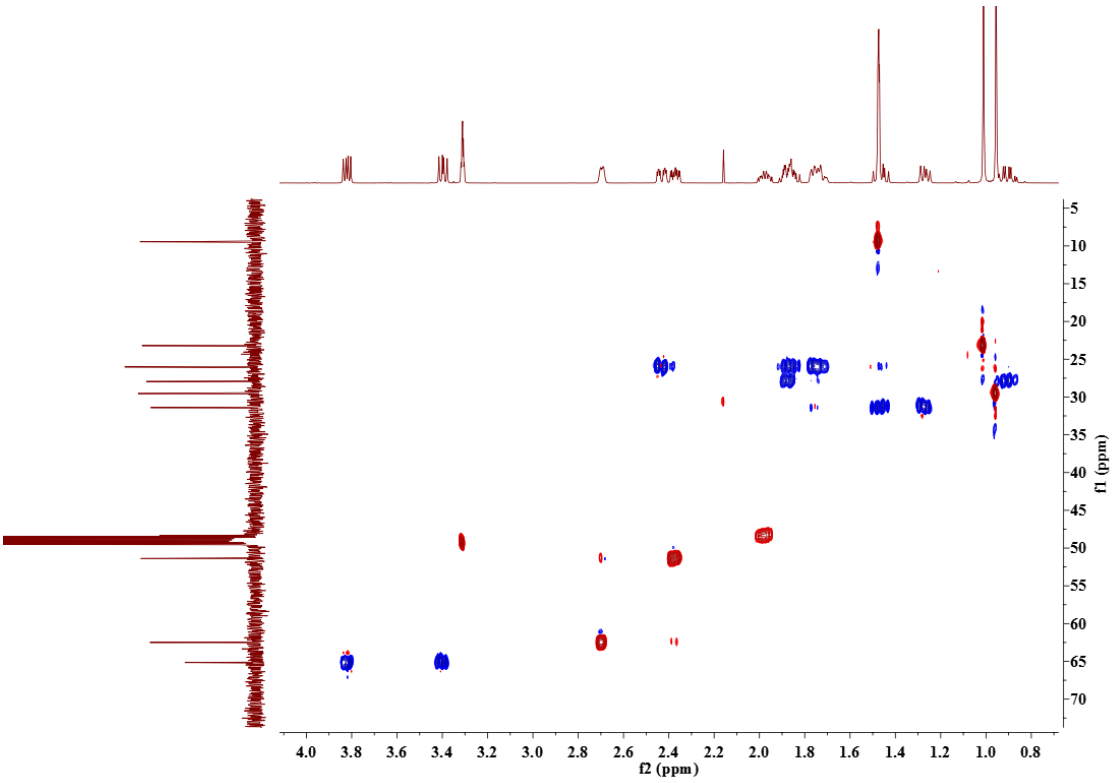

Figure S11. HSQC spectrum of compound **2**

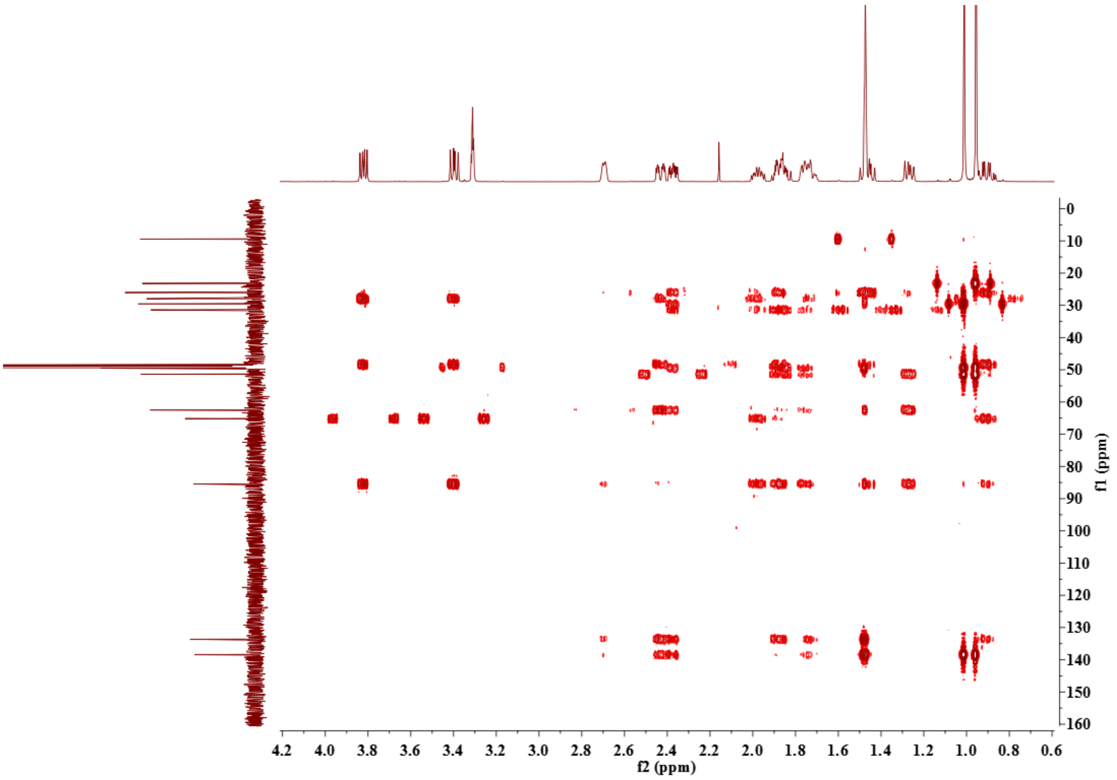

Figure S12. HMBC spectrum of compound **2**

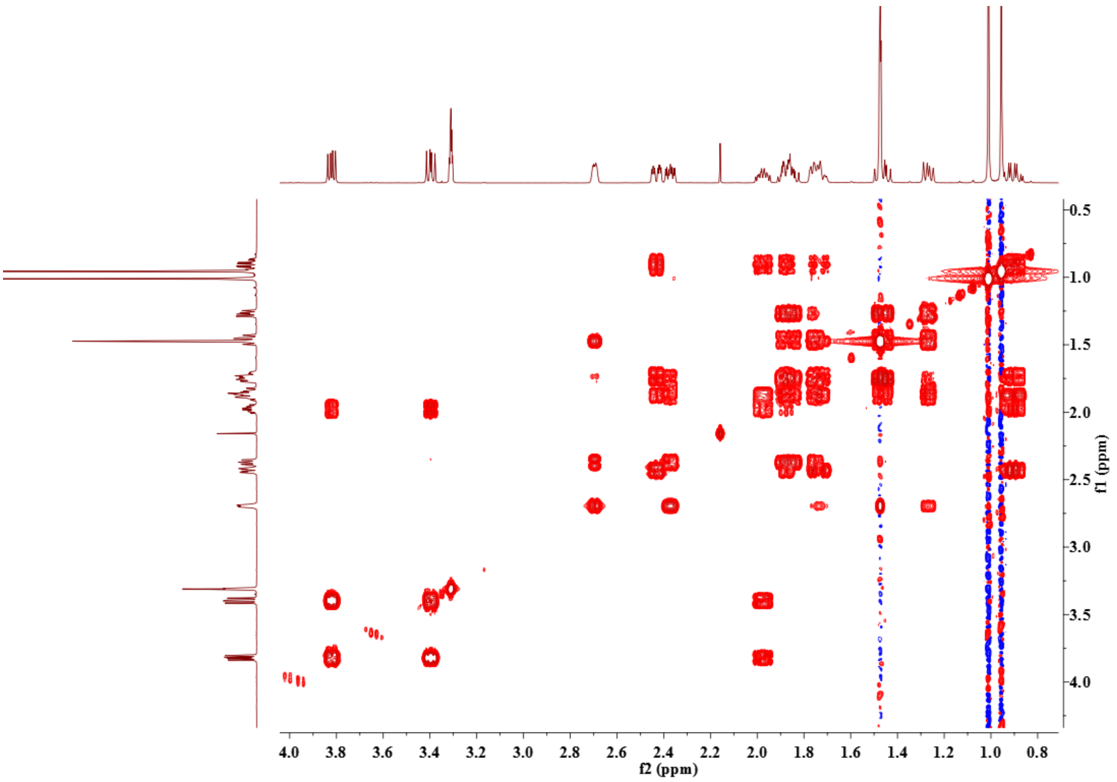

Figure S13. ^1H-^1H COSY spectrum of compound **2**

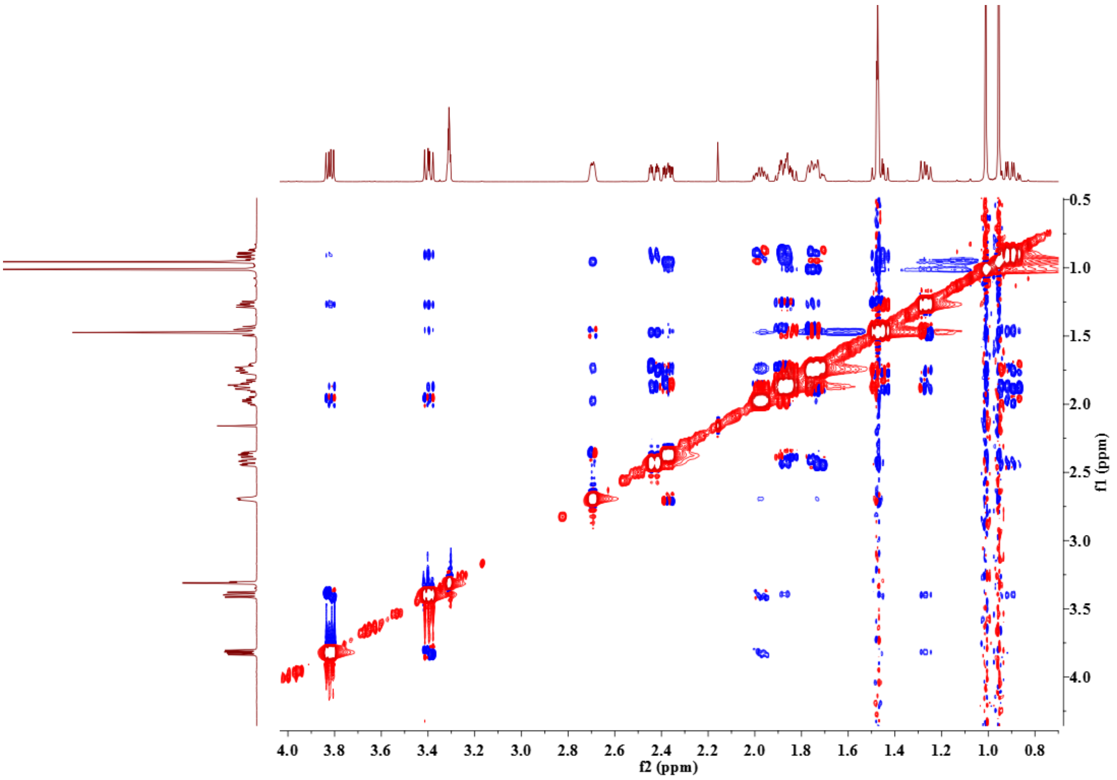

Figure S14. ROESY spectrum of compound **2**

User Spectra

Fragmentor Voltage	Collision Energy	Ionization Mode
100	0	ESI

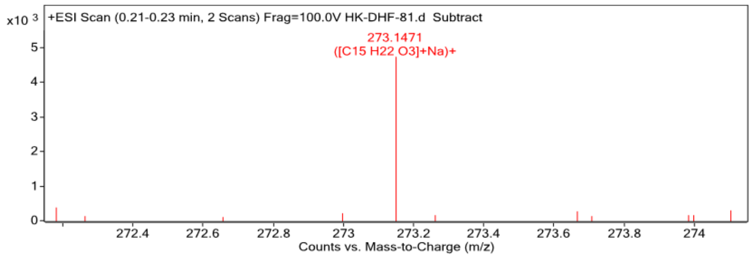

Peak List

m/z	z	Abund
274.2738	1	111865.22
291.156	1	39845.78
318.3	1	73646.97
353.2656	1	51620.16
381.2973	1	96388.89
397.271	2	60727.61
437.1937	1	111966.63
453.1677	1	106901.11

Formula Calculator Element Limits

Element	Min	Max
C	3	60
H	0	120
O	0	30

Formula Calculator Results

Formula	CalculatedMass	CalculatedMz	Mz	Diff. (mDa)	Diff. (ppm)	DBE
C15 H22 O3	250.1569	273.1461	273.1471	-1.00	-3.66	5.0000

Figure S15. HRESIMS spectrum of **3**

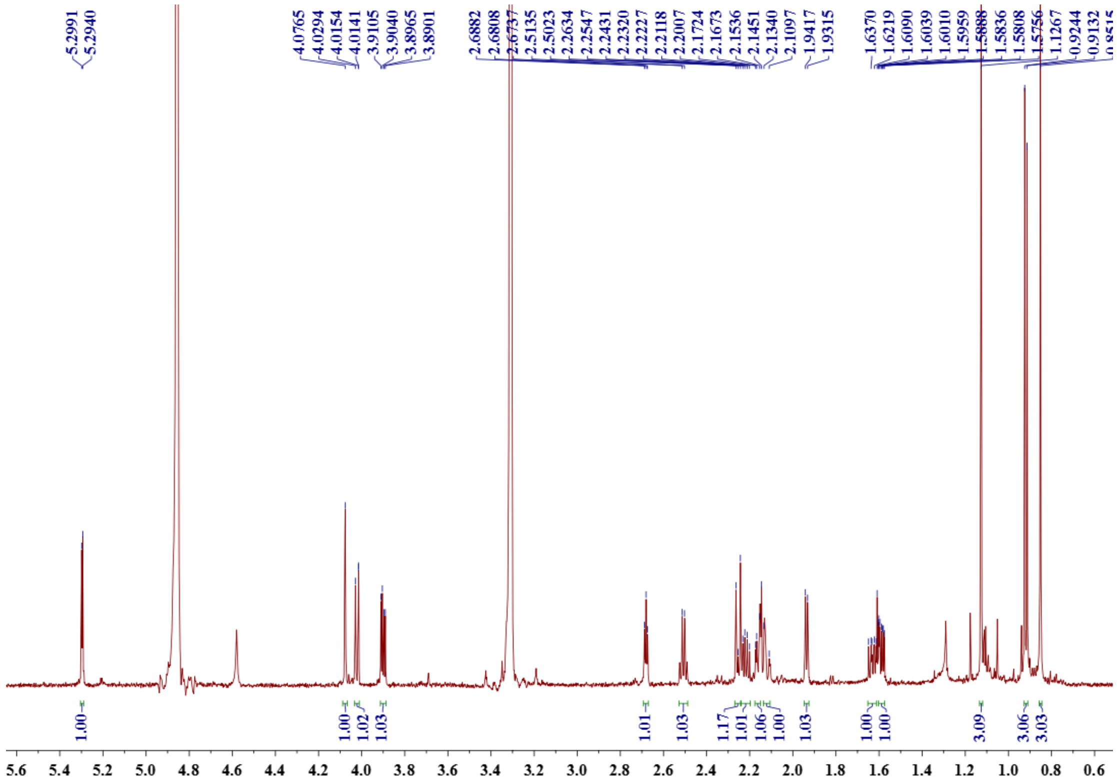

Figure S16. ^{1}H NMR spectrum of compound **3** in MeOH-d_4 (500 MHz)

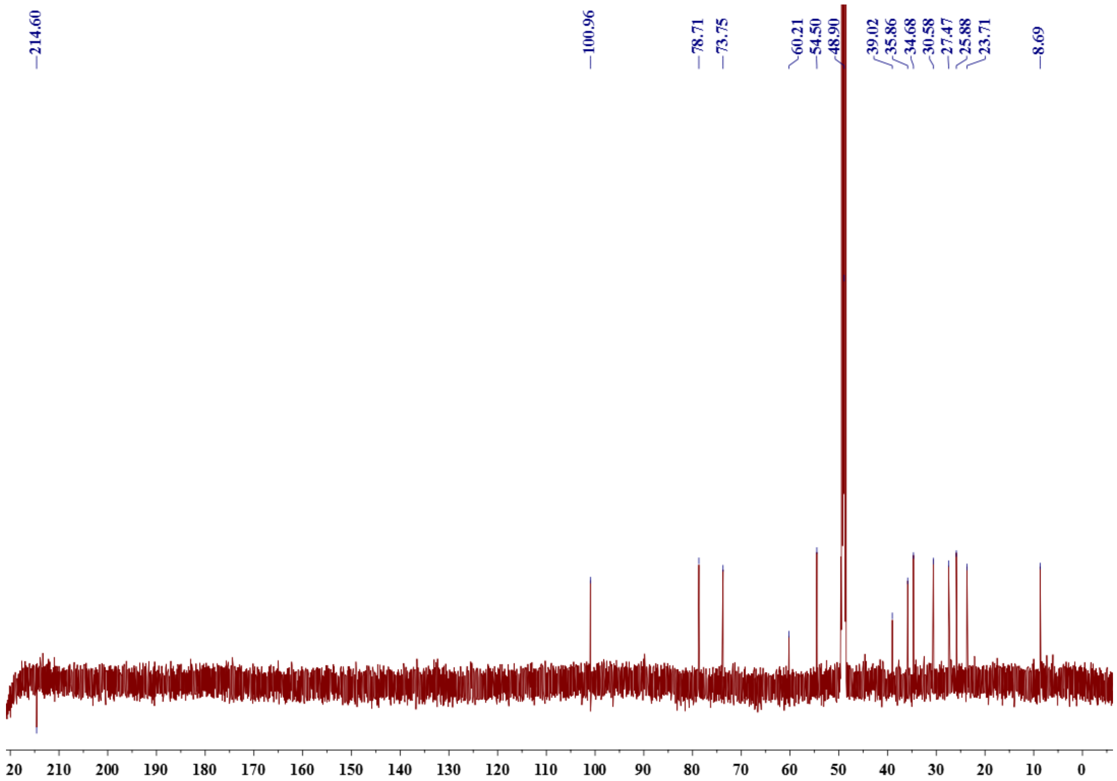

Figure S17. ^{13}C NMR spectrum of compound **3** in MeOH-d_4 (125 MHz)

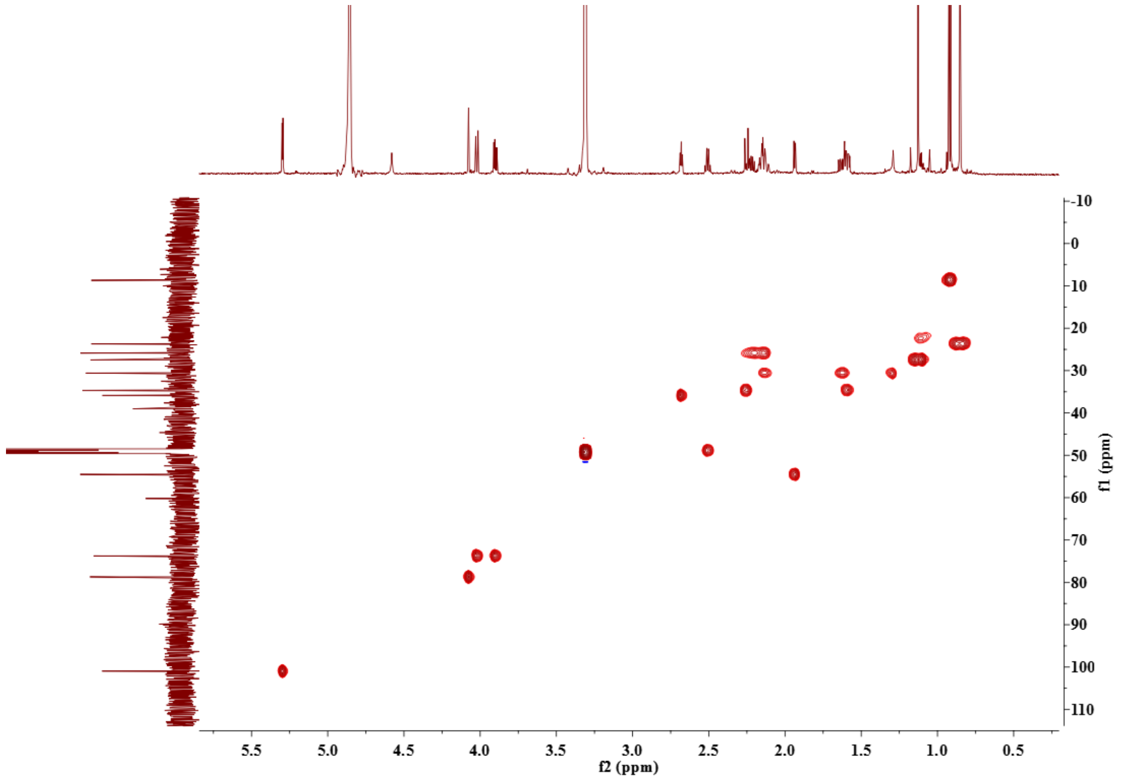

Figure S18. HSQC spectrum of compound **3**

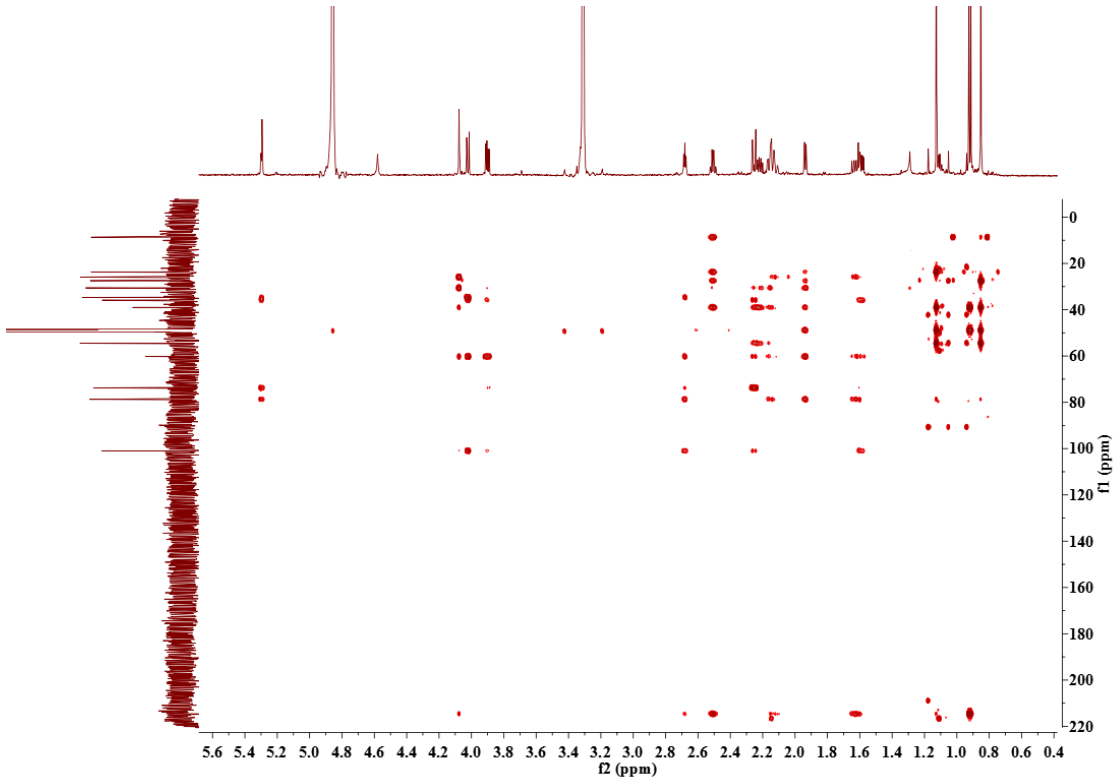

Figure S19. HMBC spectrum of compound **3**

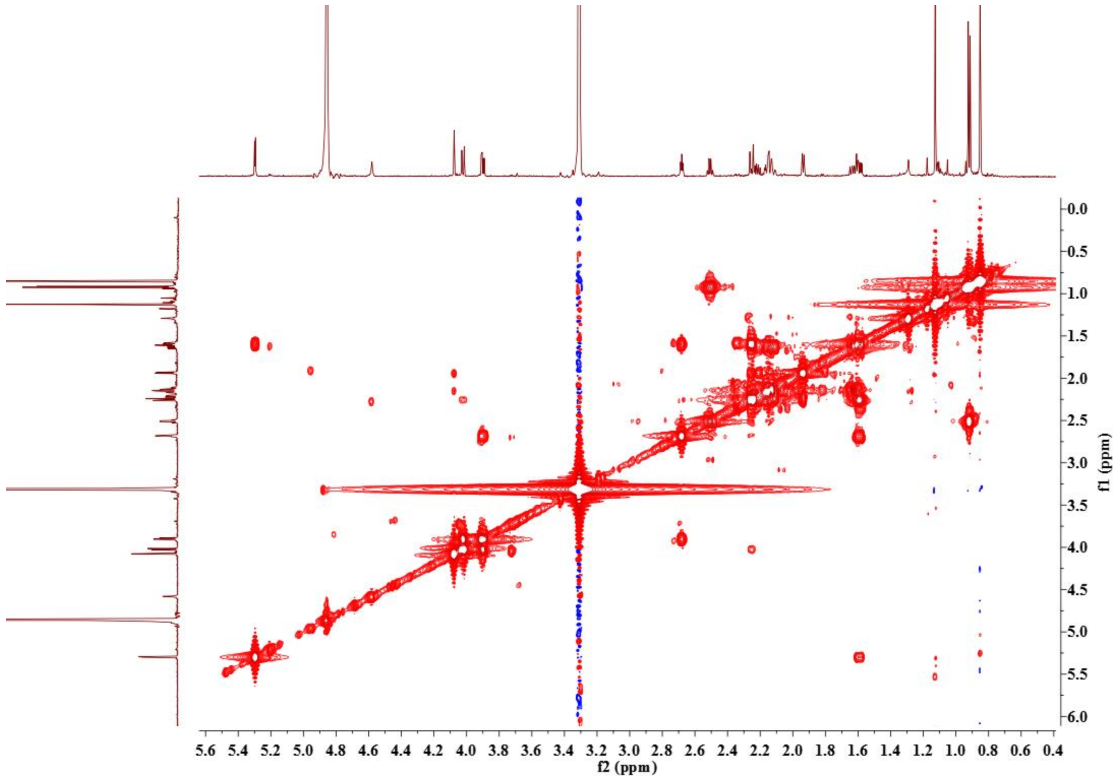

Figure S20. ^{1}H-^{1}H COSY spectrum of compound **3**

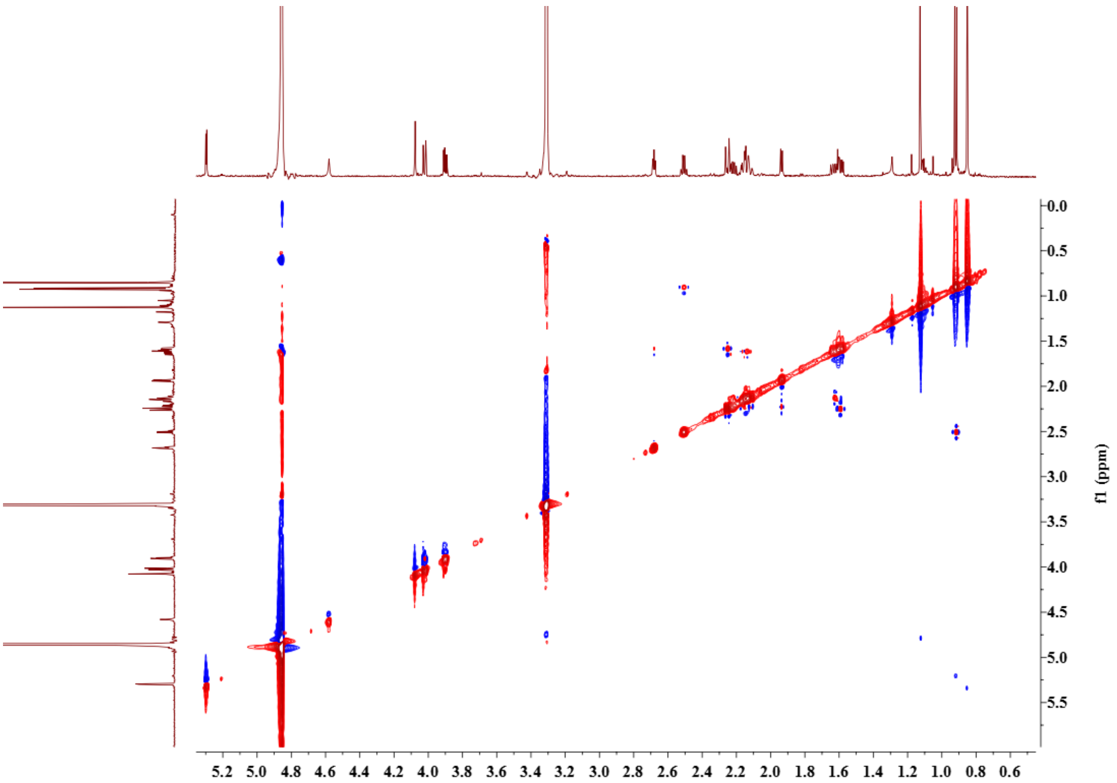

Figure S21. ROESY spectrum of compound **3**

User Spectra

Fragmentor Voltage	Collision Energy	Ionization Mode
135	0	ESI

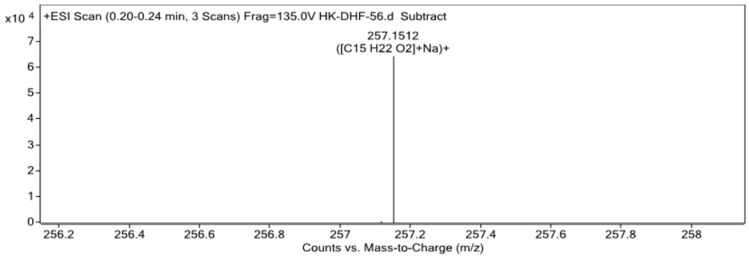

Peak List

m/z	z	Abund	Formula	Ion
235.1695	1	56303.95		
257.1512	1	64473.5	C15 H22 O2	(M+Na)+
258.1545	1	10245.92	C15 H22 O2	(M+Na)+
273.1254	1	9913		
273.1453		17628.07		
489.2976	2	14429.4		
491.3134	1	49801.79		
492.3165	1	14918.61		

Formula Calculator Element Limits

Element	Min	Max
C	3	60
H	0	120
O	0	30

Formula Calculator Results

Formula	CalculatedMass	CalculatedMz	Mz	Diff. (mDa)	Diff. (ppm)	DBE
C15 H22 O2	234.1620	257.1512	257.1512	0.00	0.00	5.0000

Figure S22. HRESIMS spectrum of **4**

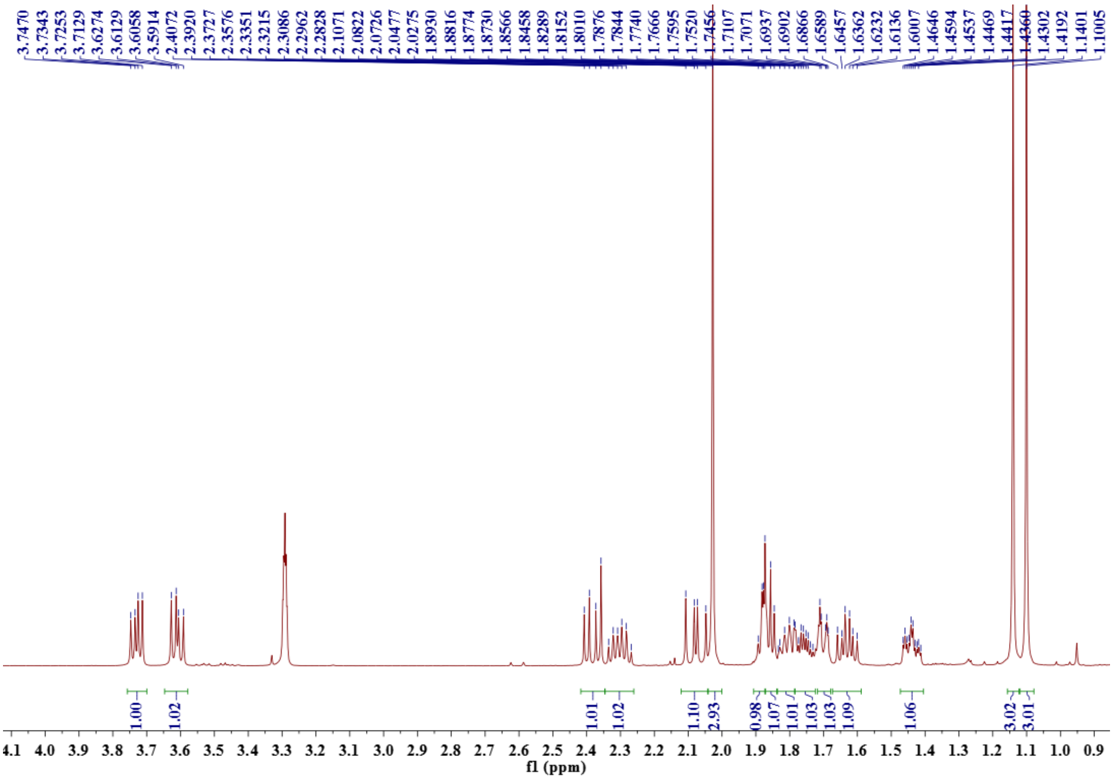

Figure S23. ^{1}H NMR spectrum of compound **4** in MeOH-d_4 (500 MHz)

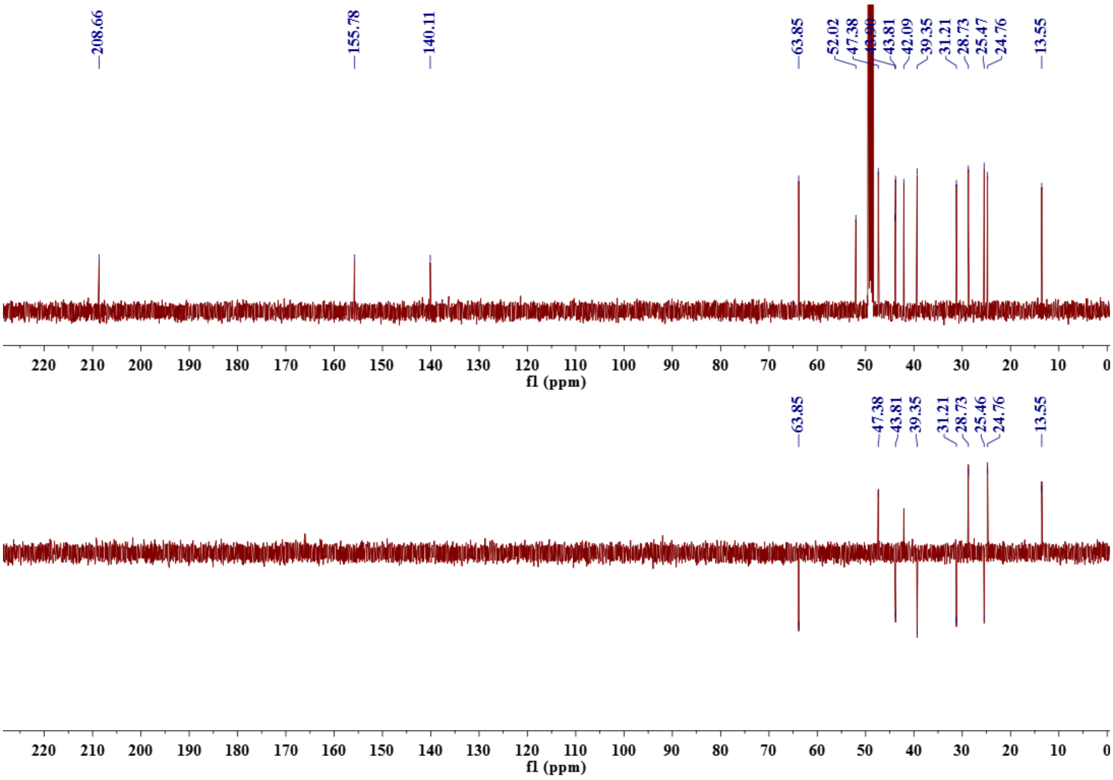

Figure S24. ^{13}C NMR spectrum of compound **4** in MeOH-d_4 (125 MHz)

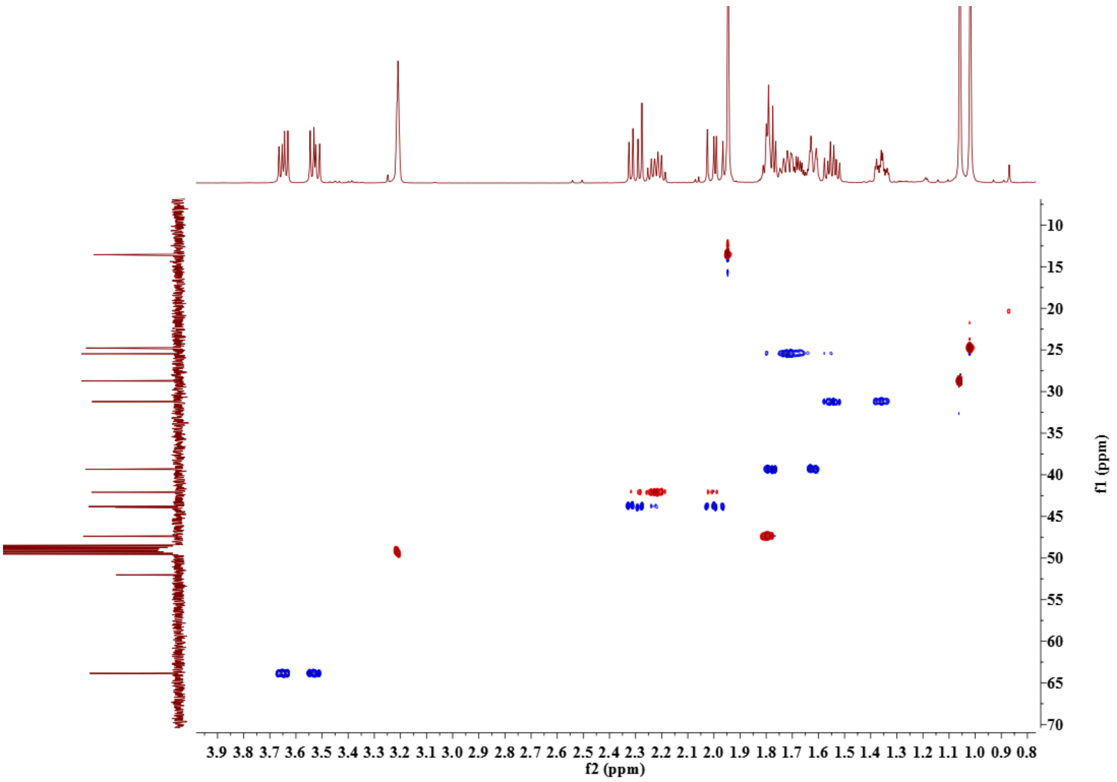

Figure S25. HSQC spectrum of compound **4**

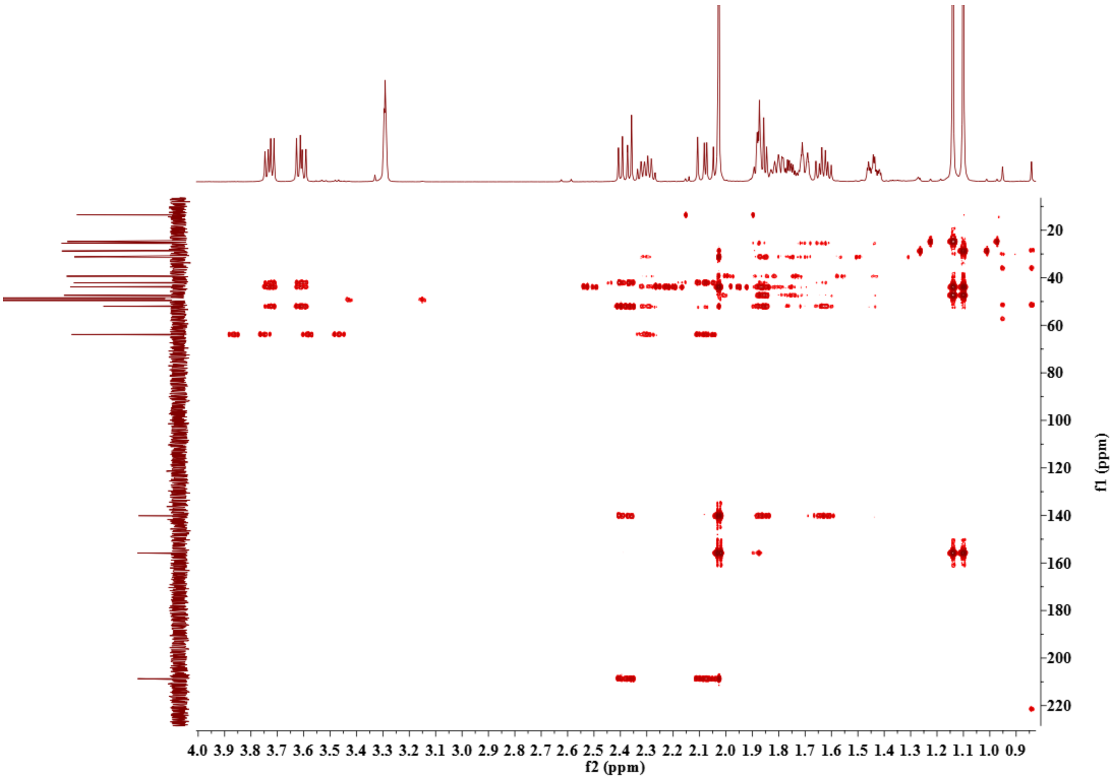

Figure S26. HMBC spectrum of compound **4**

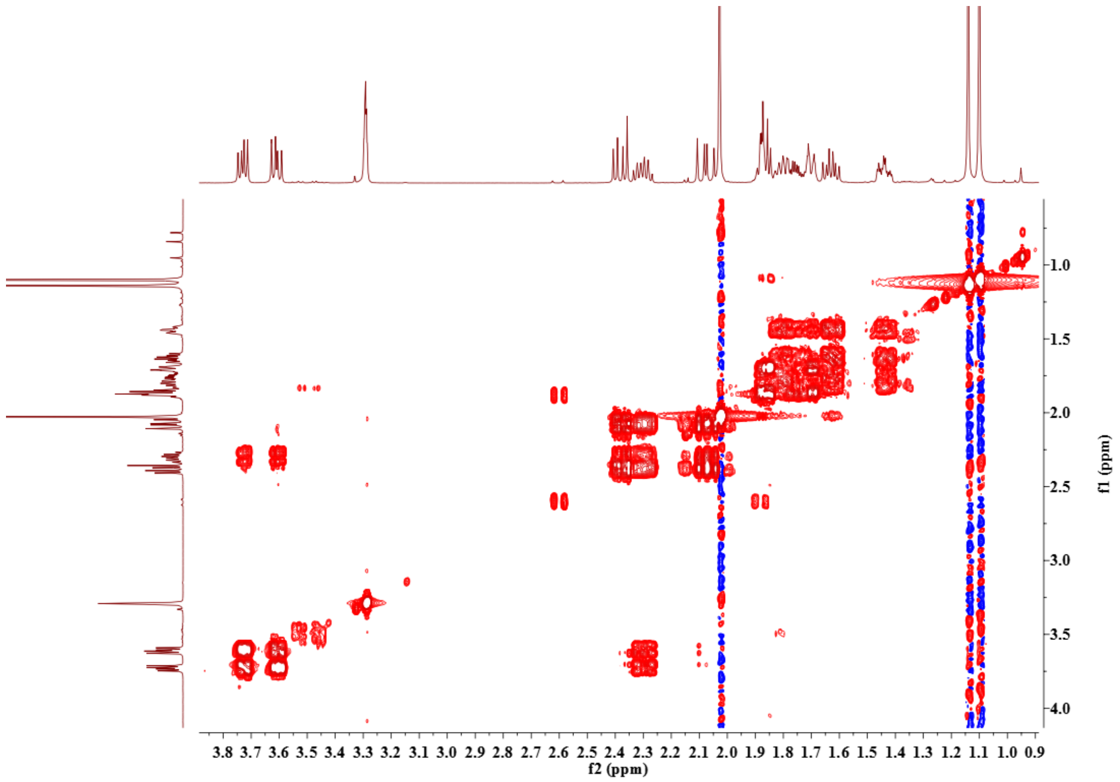

Figure S27. ^{1}H-^{1}H COSY spectrum of compound **4**

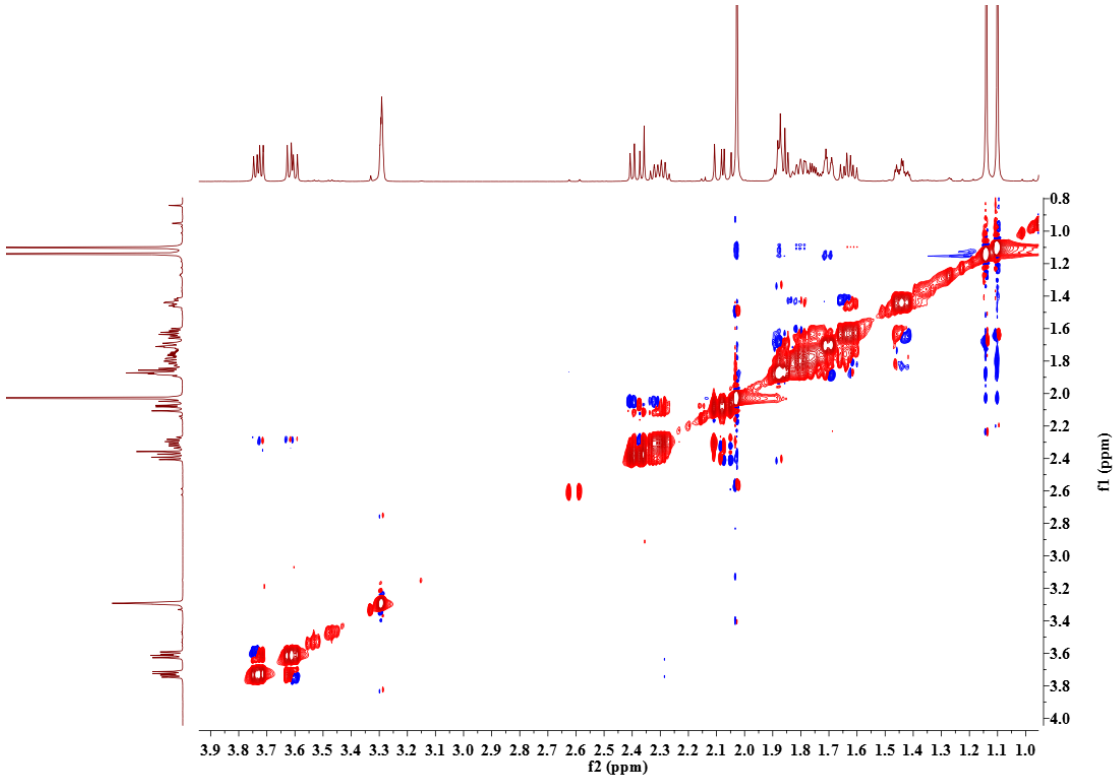

Figure S28. ROESY spectrum of compound **4**

User Spectra

Fragmentor Voltage	Collision Energy	Ionization Mode
135	0	ESI

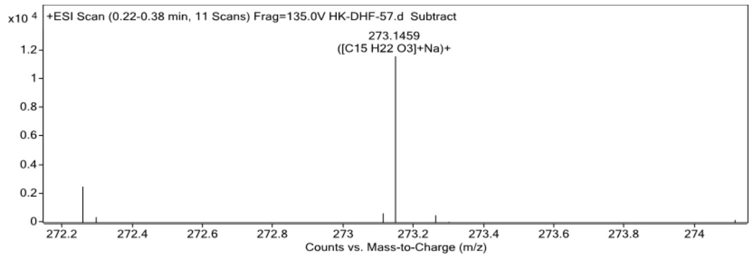

Peak List

m/z	z	Abund
102.1276	1	39549.34
274.2744	1	311815.34
275.2773	1	47675.52
302.3053	1	59656.9
318.3006	1	216061.95
346.3316	1	40005.96
362.3265	1	55441.69
437.1935	1	49964.6

Formula Calculator Element Limits

Element	Min	Max
C	3	60
H	0	120
O	0	30

Formula Calculator Results

Formula	CalculatedMass	CalculatedMz	Mz	Diff. (mDa)	Diff. (ppm)	DBE
C15 H22 O3	250.1569	273.1461	273.1459	0.20	0.73	5.0000

Figure S29. HRESIMS spectrum of **5**

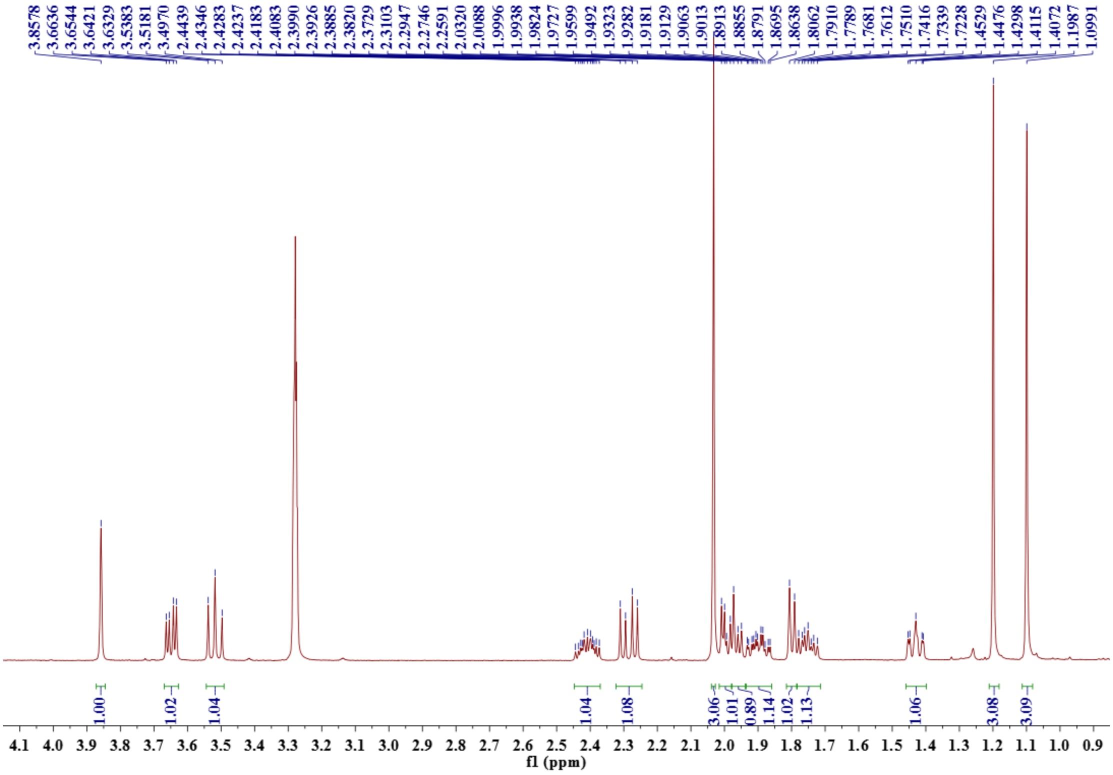

Figure S30. ^{1}H NMR spectrum of compound **5** in MeOH-d_4 (500 MHz)

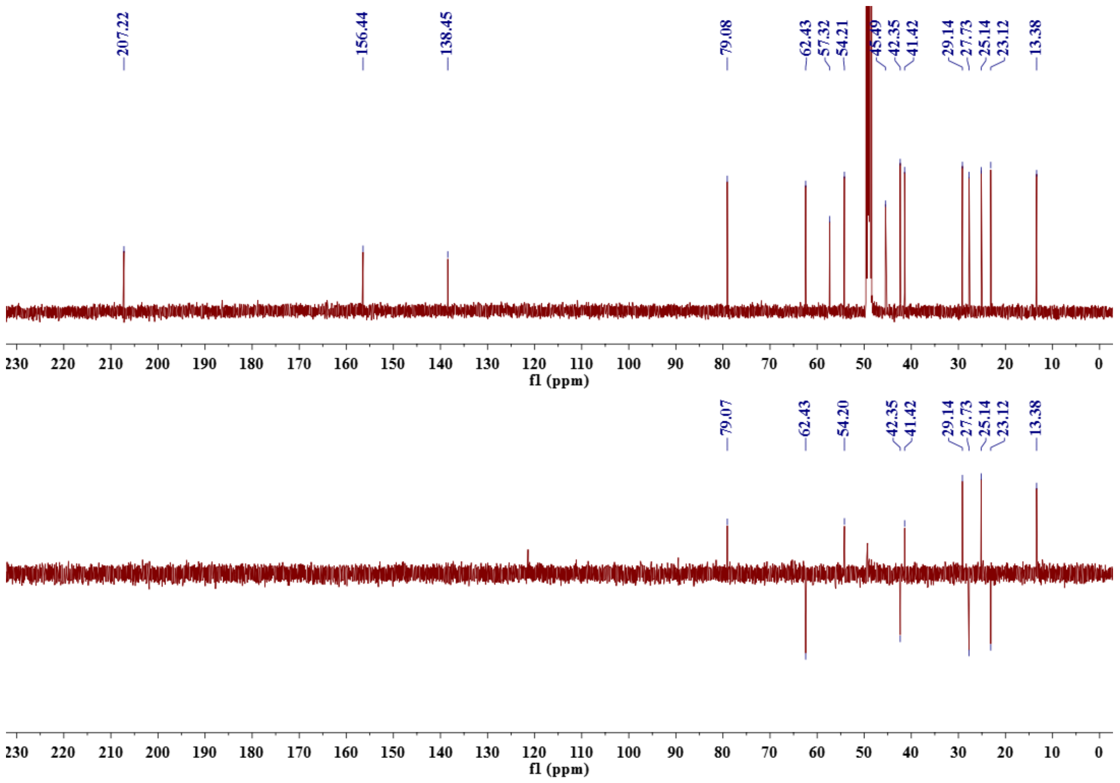

Figure S31. ^{13}C NMR spectrum of compound **5** in MeOH-d_4 (125 MHz)

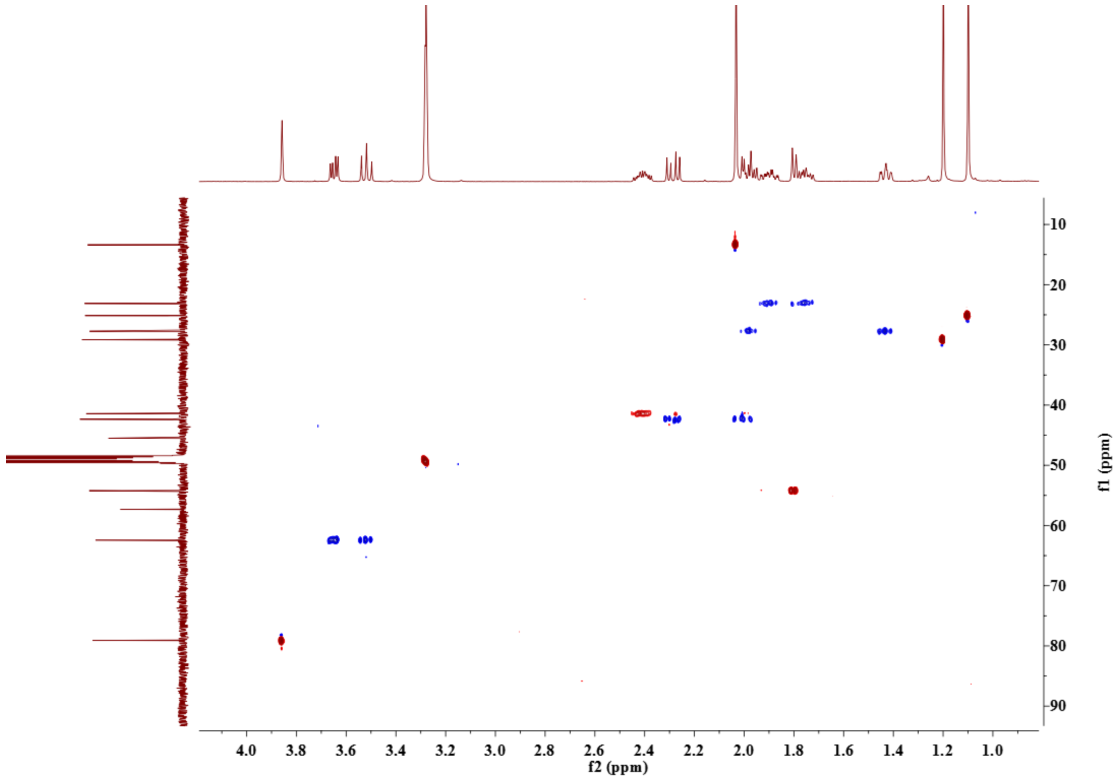

Figure S32. HSQC spectrum of compound **5**

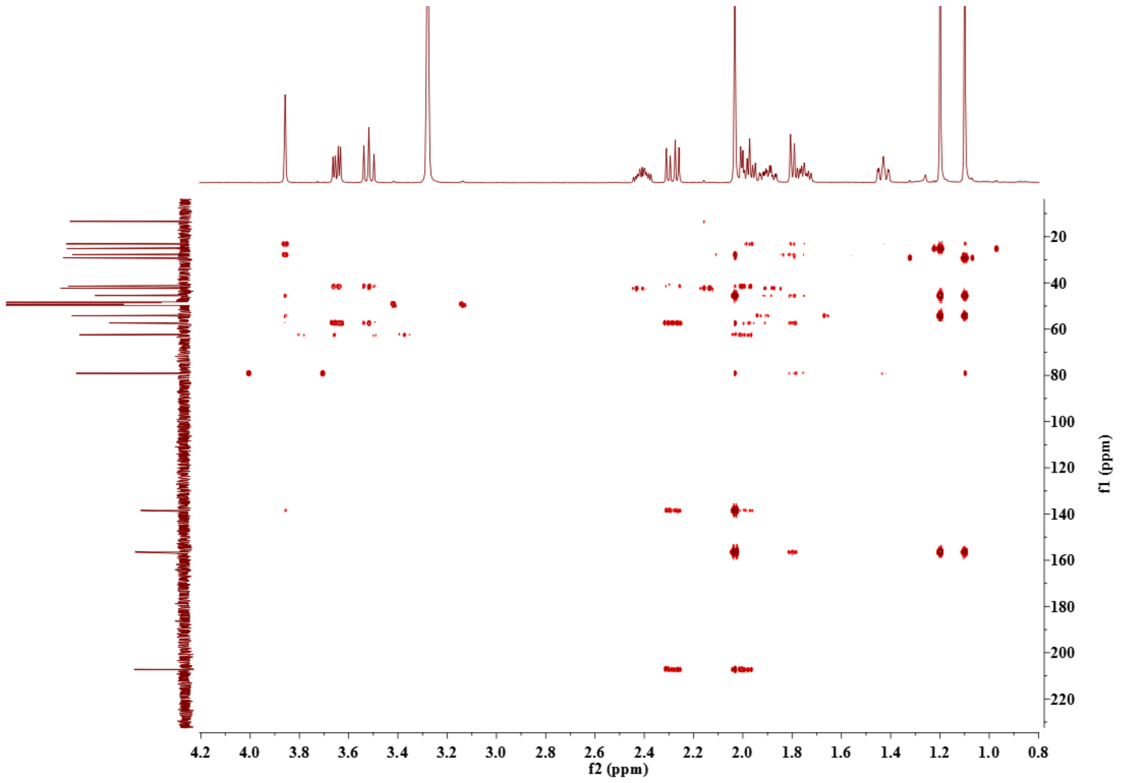

Figure S33. HMBC spectrum of compound **5**

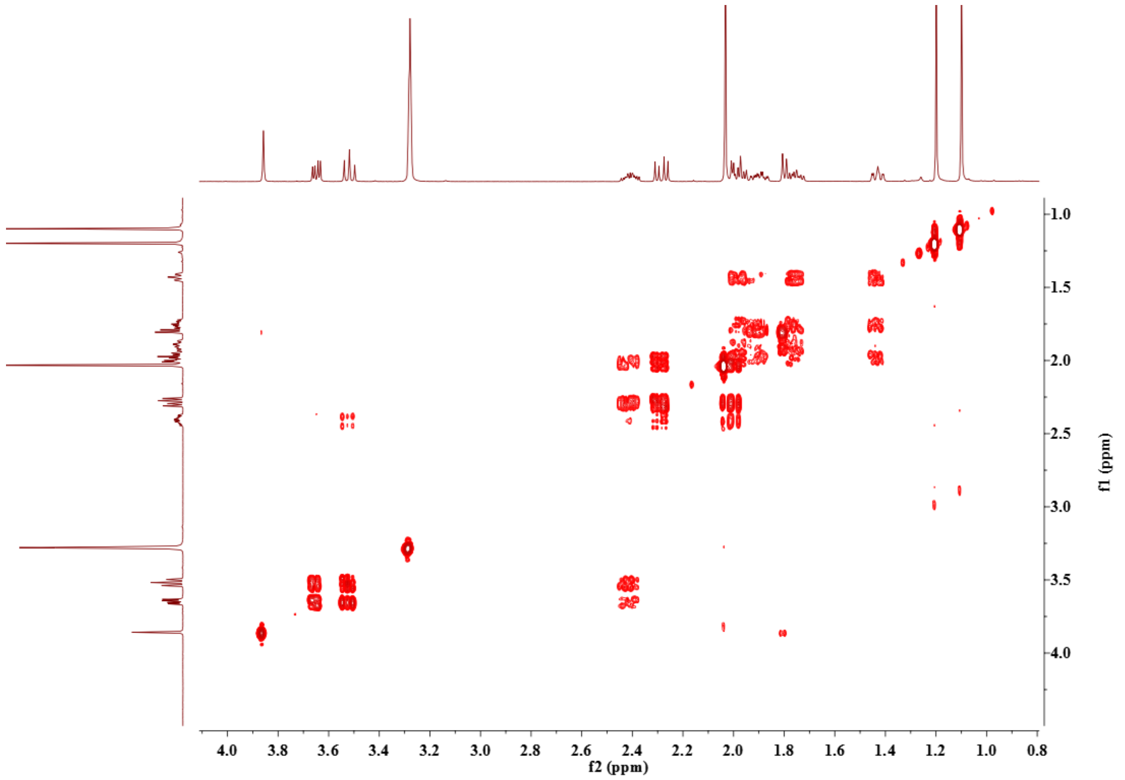

Figure S34. ^{1}H-^{1}H COSY spectrum of compound **5**

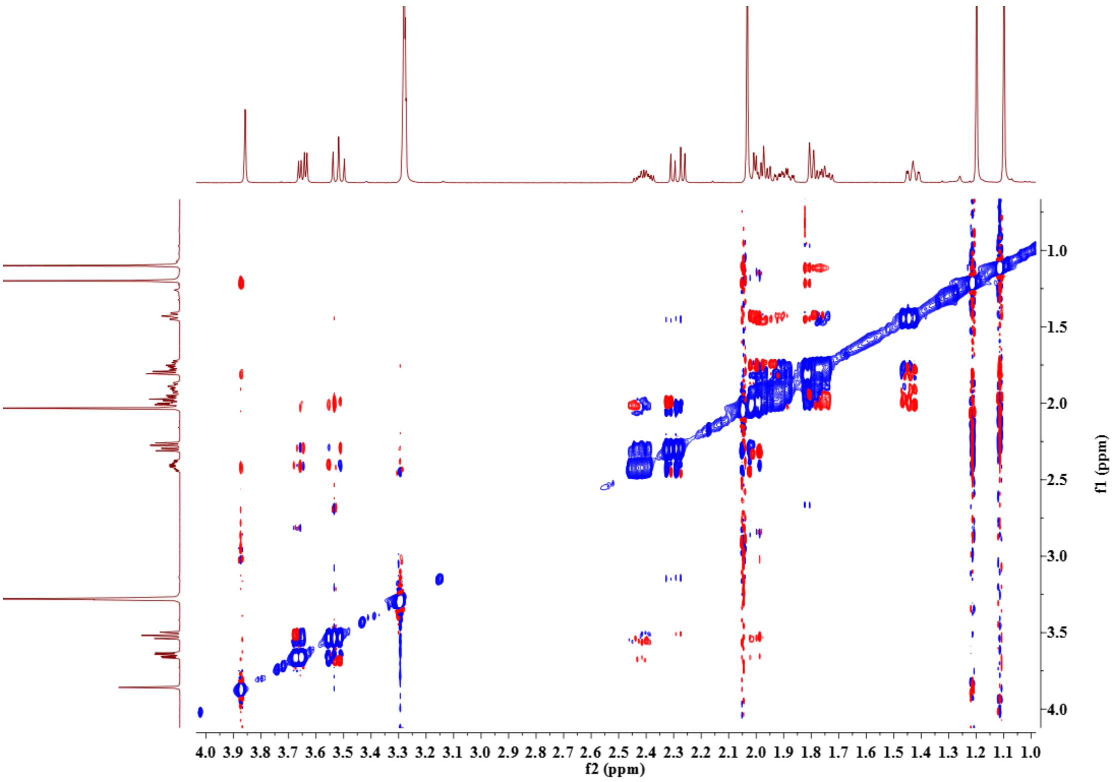

Figure S35. ROESY spectrum of compound **5**

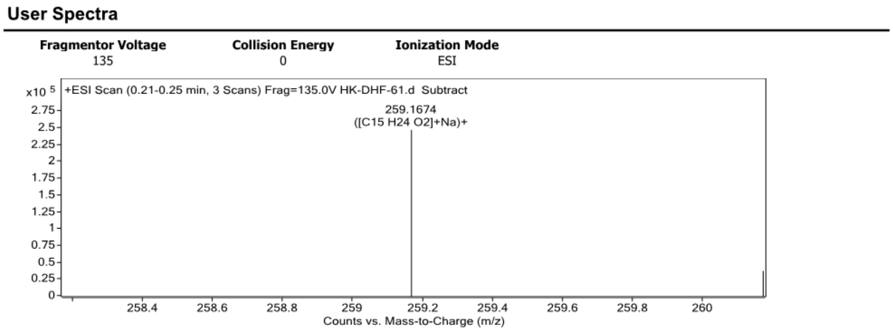

Peak List

m/z	z	Abund	Formula	Ion
201.1641	1	46710.49		
219.1749	1	239824.67		
259.1674	1	247080.81	C15 H24 O2	(M+Na)+
260.1706	1	38159.66	C15 H24 O2	(M+Na)+
473.3636	1	253629.38		
474.3666	1	72865.18		
495.345	1	133527.08		
496.3482	1	37674.81		

Formula Calculator Element Limits

Element	Min	Max
C	3	60
H	0	120
O	0	30

Formula Calculator Results

Formula	CalculatedMass	CalculatedMz	Mz	Diff. (mDa)	Diff. (ppm)	DBE
C15 H24 O2	236.1776	259.1669	259.1674	-0.50	-1.93	4.0000

Figure S36. HRESIMS spectrum of **6**

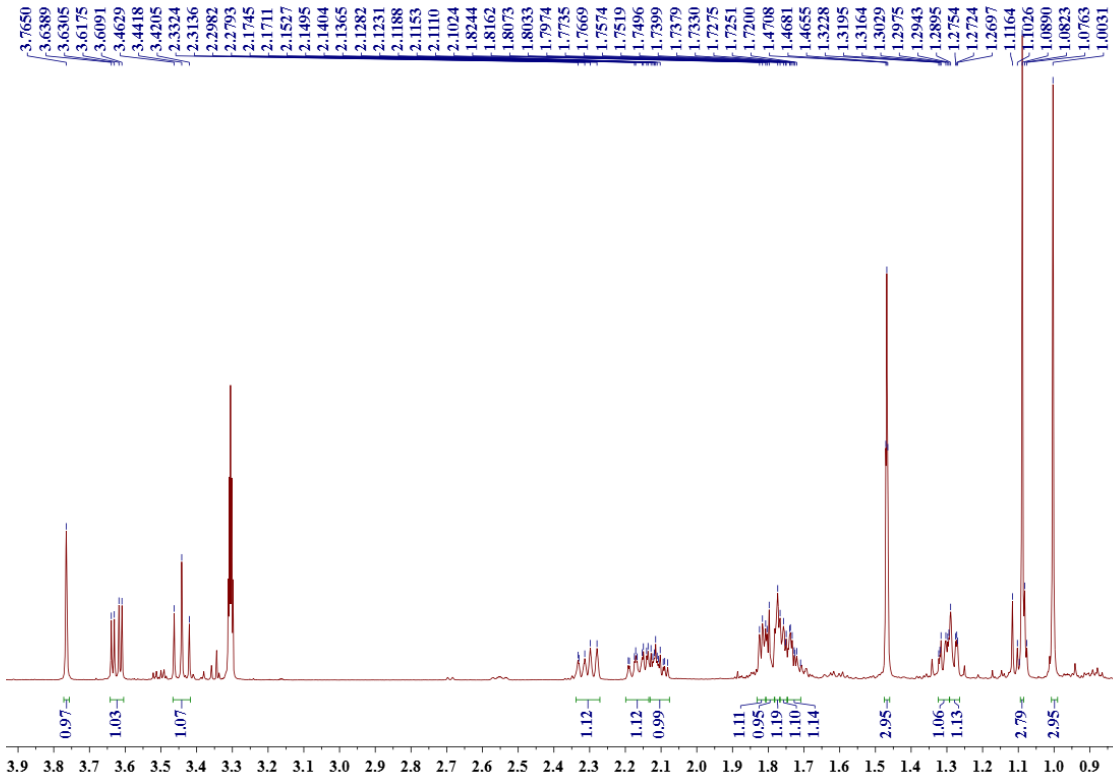

Figure S37. ^{1}H NMR spectrum of compound **6** in MeOH-d_4 (500 MHz)

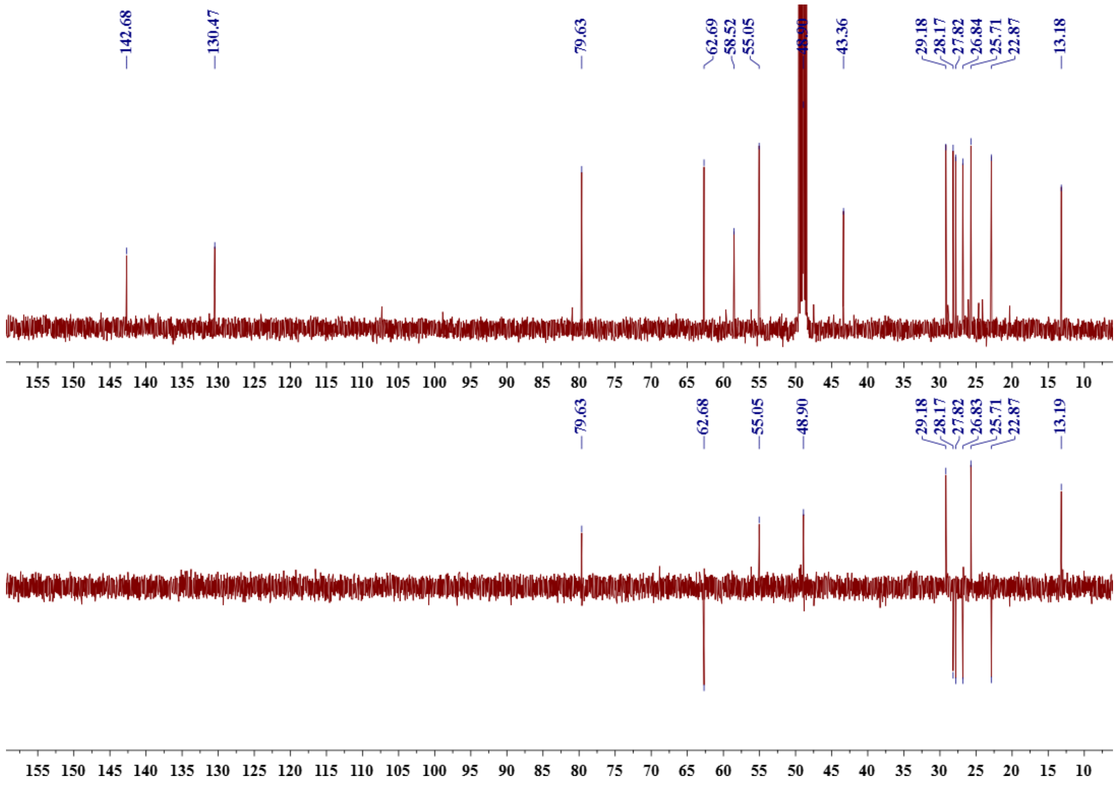

Figure S38. ^{13}C NMR spectrum of compound **6** in MeOH-d_4 (125 MHz)

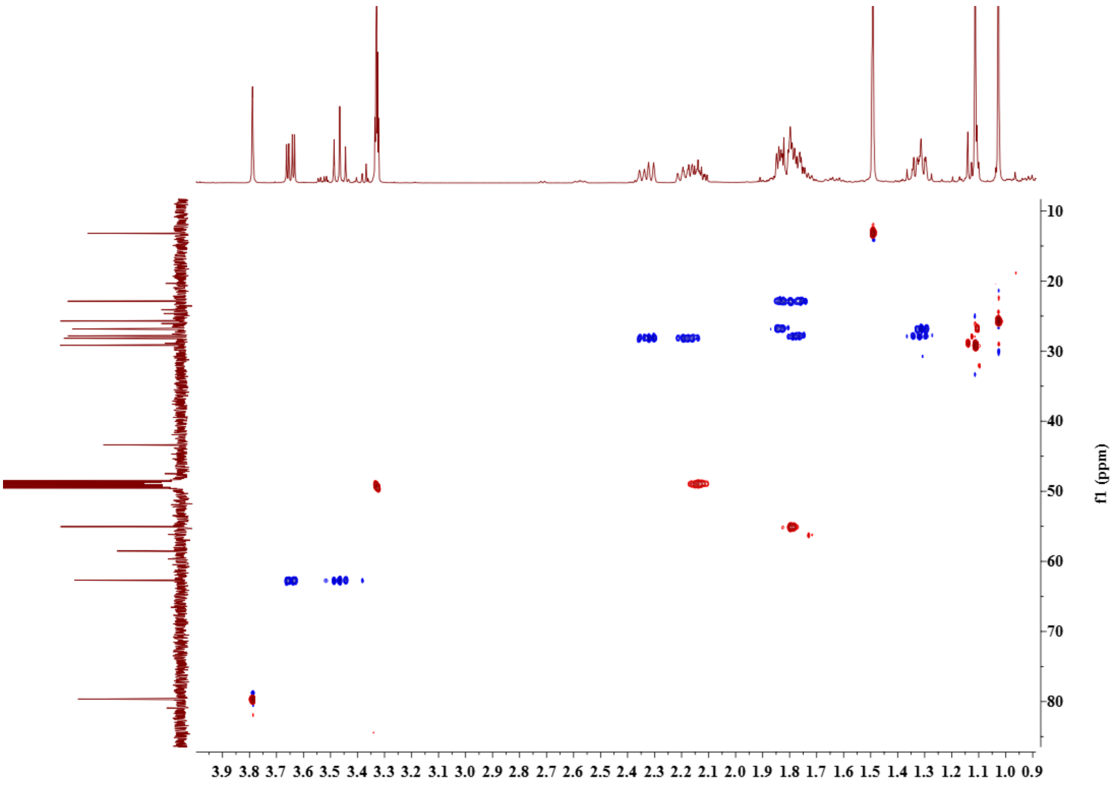

Figure S39. HSQC spectrum of compound **6**

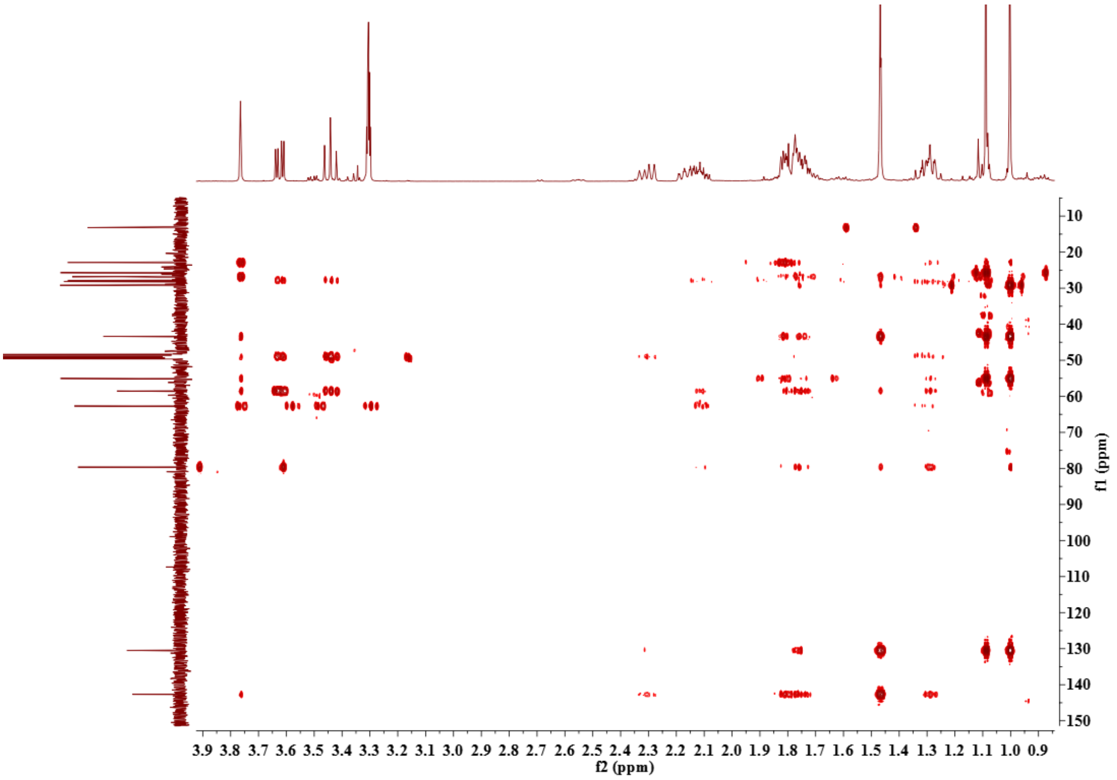

Figure S40. HMBC spectrum of compound **6**

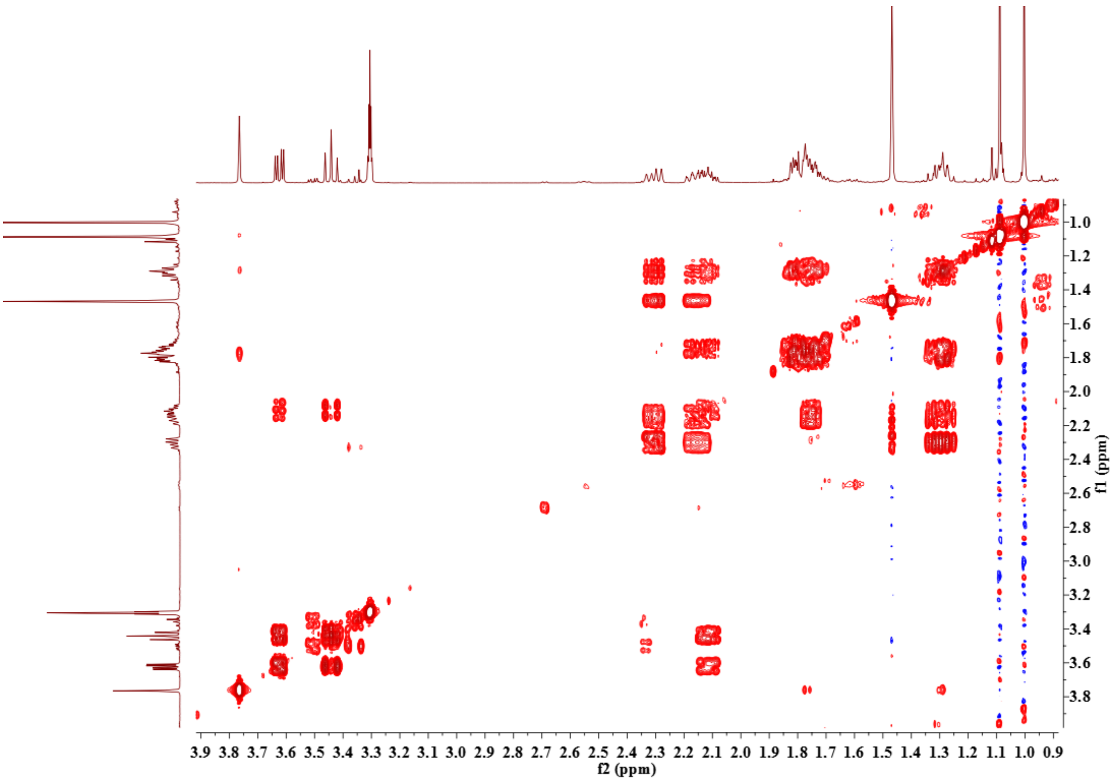

Figure S41. ^{1}H-^{1}H COSY spectrum of compound **6**

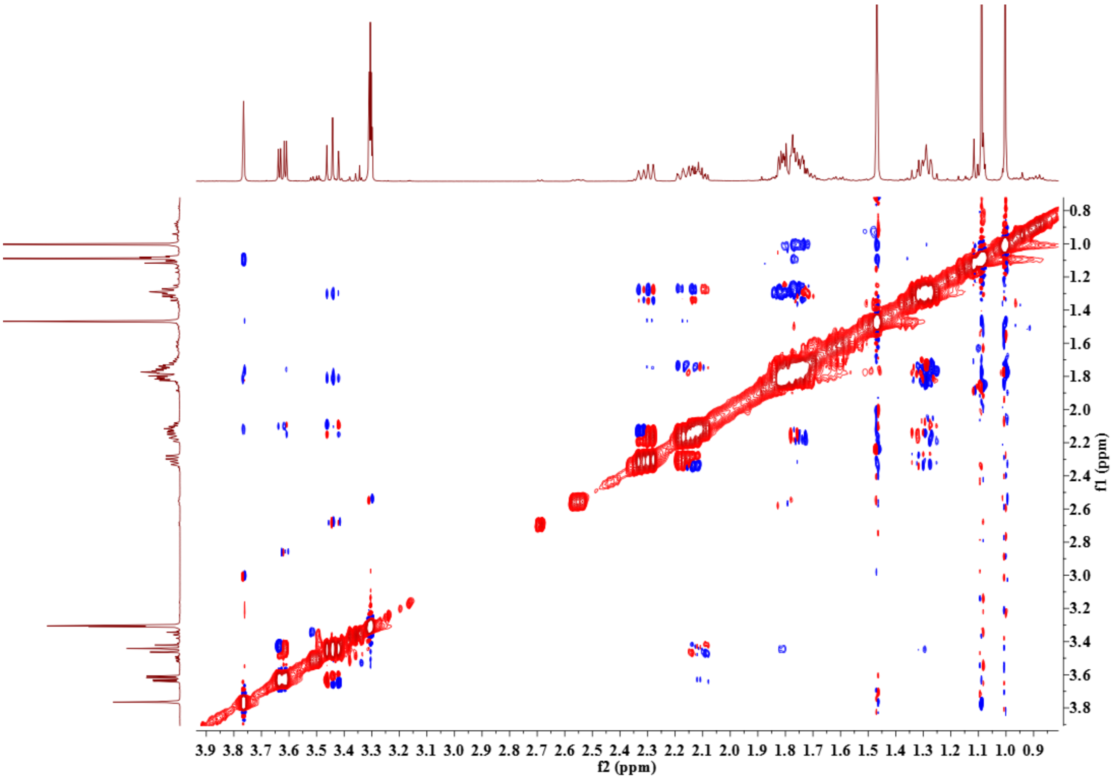

Figure S42. ROESY spectrum of compound **6**

User Spectra

Fragmentor Voltage	Collision Energy	Ionization Mode
135	0	ESI

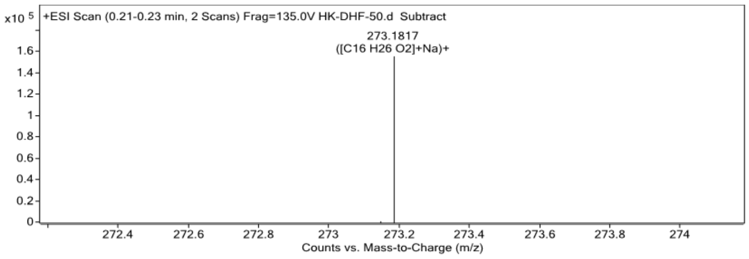

Peak List

m/z	z	Abund	Formula	Ion
201.1627	1	37207.45		
219.1734	1	111746.36		
220.1766	1	16721.34		
273.1817	1	155676.19	C16 H26 O2	(M+Na)+
274.1849	1	24313.38	C16 H26 O2	(M+Na)+
274.273	1	11027.4		
453.1658	1	7287.61		
523.3739	1	11038.77		

Formula Calculator Element Limits

Element	Min	Max
C	3	60
H	0	120
O	0	30

Formula Calculator Results

Formula	CalculatedMass	CalculatedMz	Mz	Diff. (mDa)	Diff. (ppm)	DBE
C16 H26 O2	250.1933	273.1825	273.1817	0.80	2.93	4.0000

Figure S43. HRESIMS spectrum of **7**

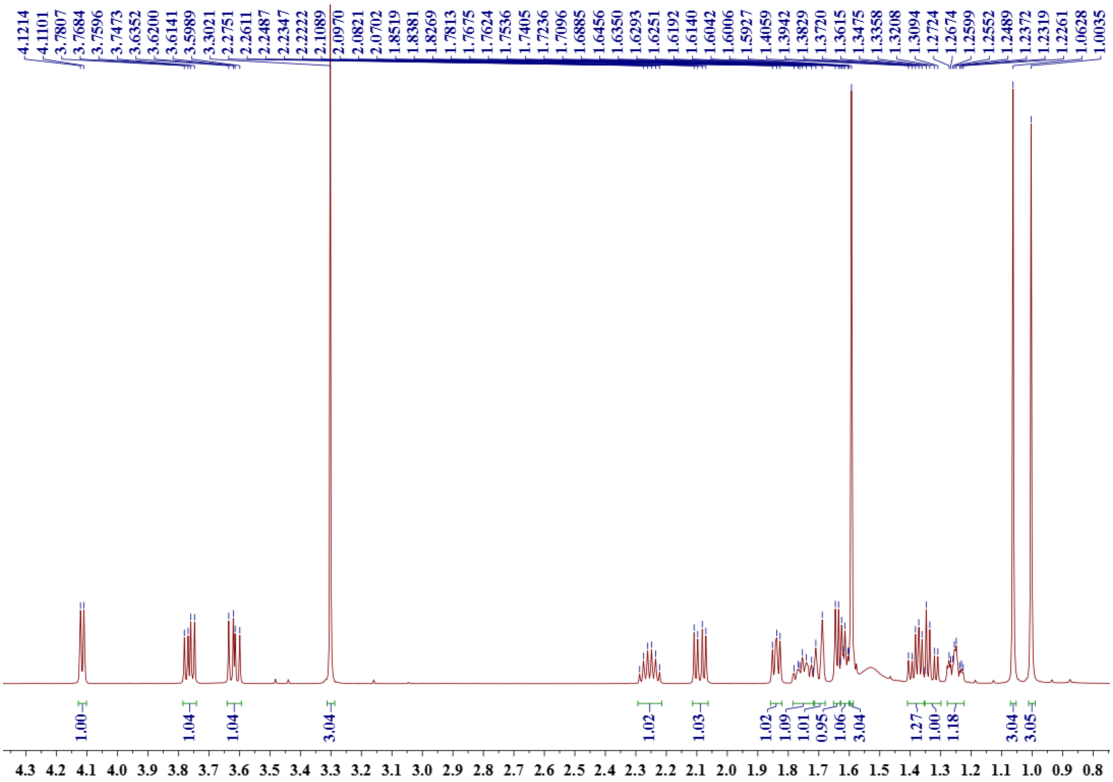

Figure S44. ^{1}H NMR spectrum of compound **7** in $CDCl_3$ (500 MHz)

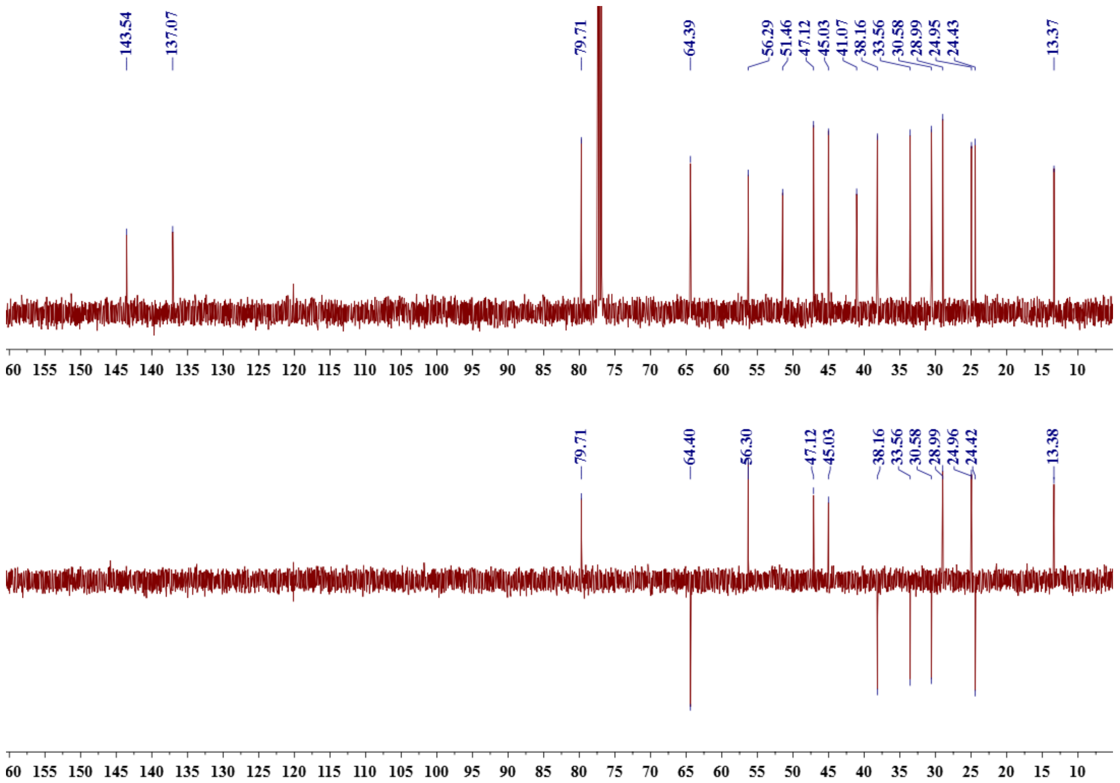

Figure S45. ^{13}C NMR spectrum of compound **7** in $CDCl_3$ (125 MHz)

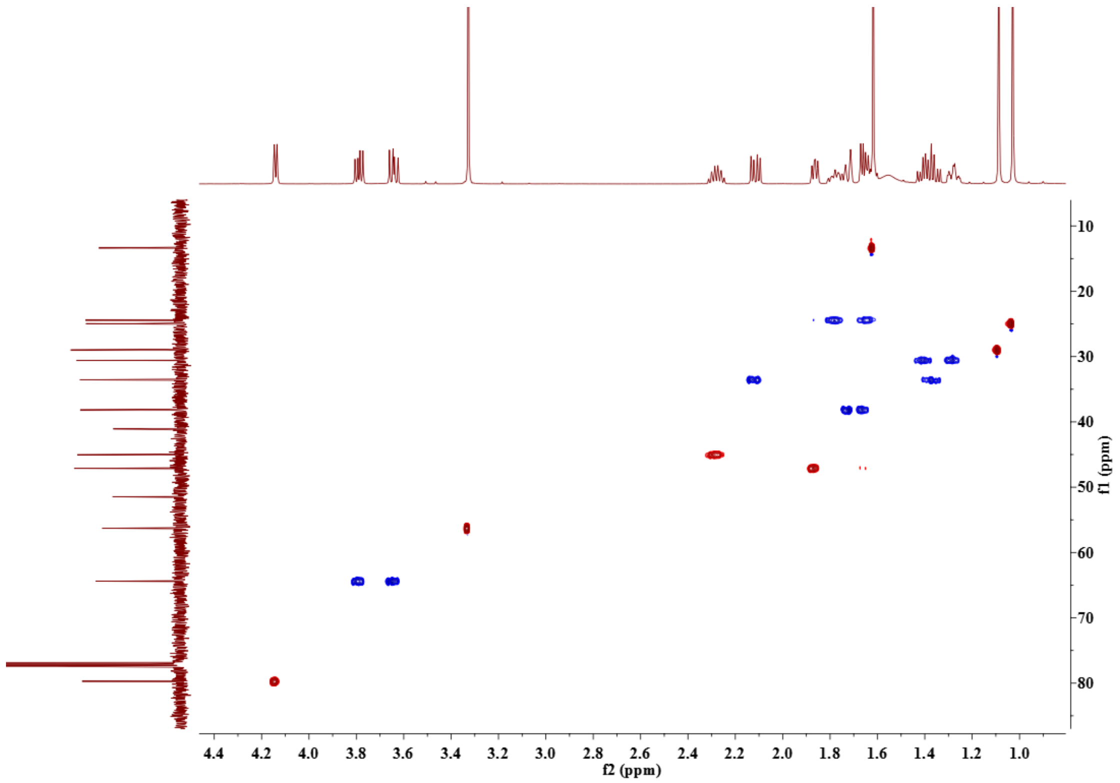

Figure S46. HSQC spectrum of compound **7**

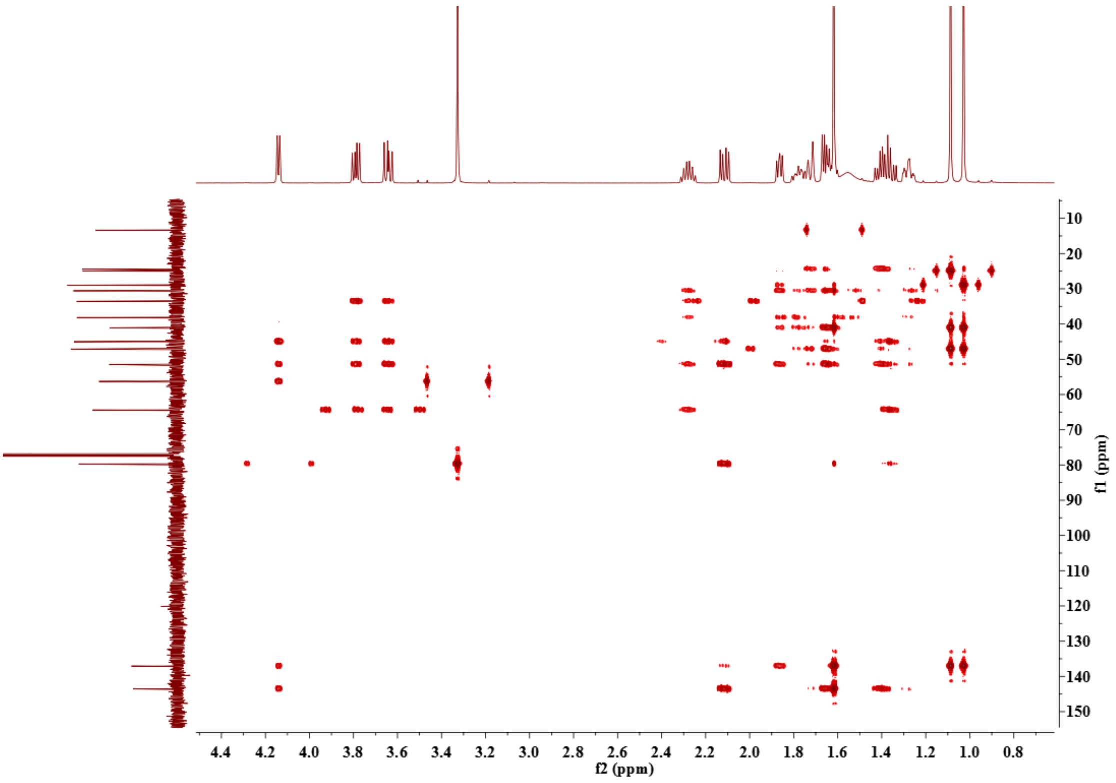

Figure S47. HMBC spectrum of compound **7**

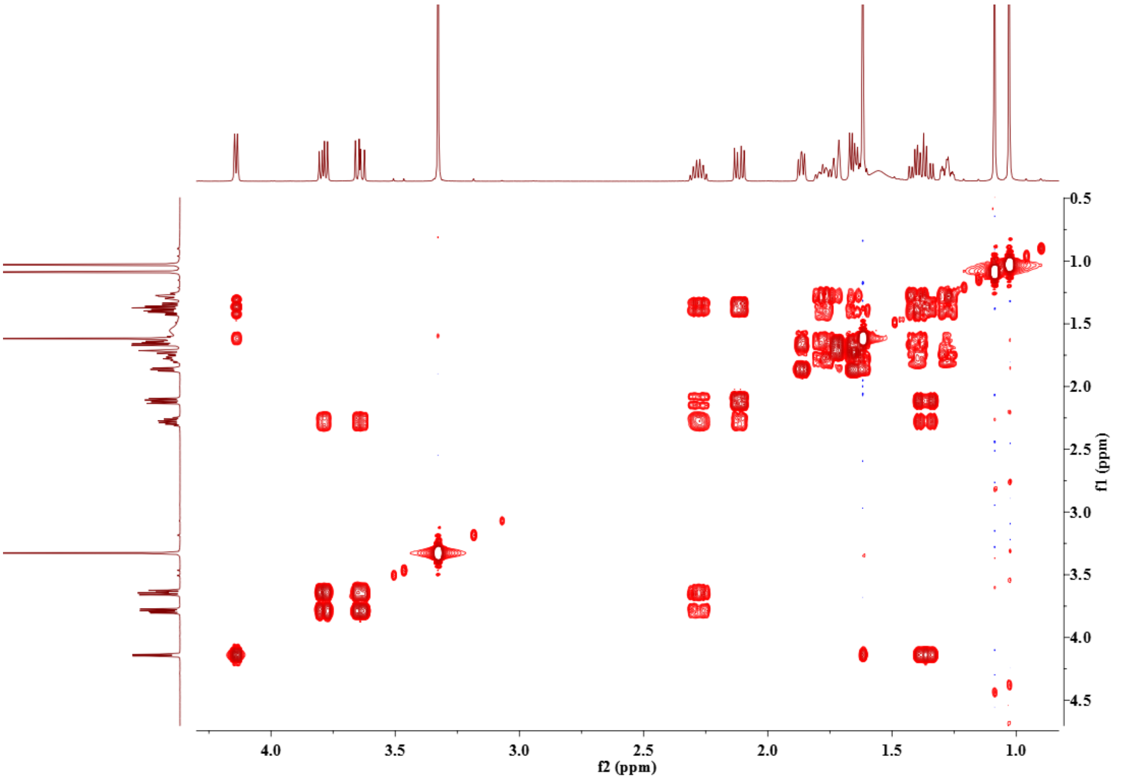

Figure S48. ^{1}H-^{1}H COSY spectrum of compound **7**

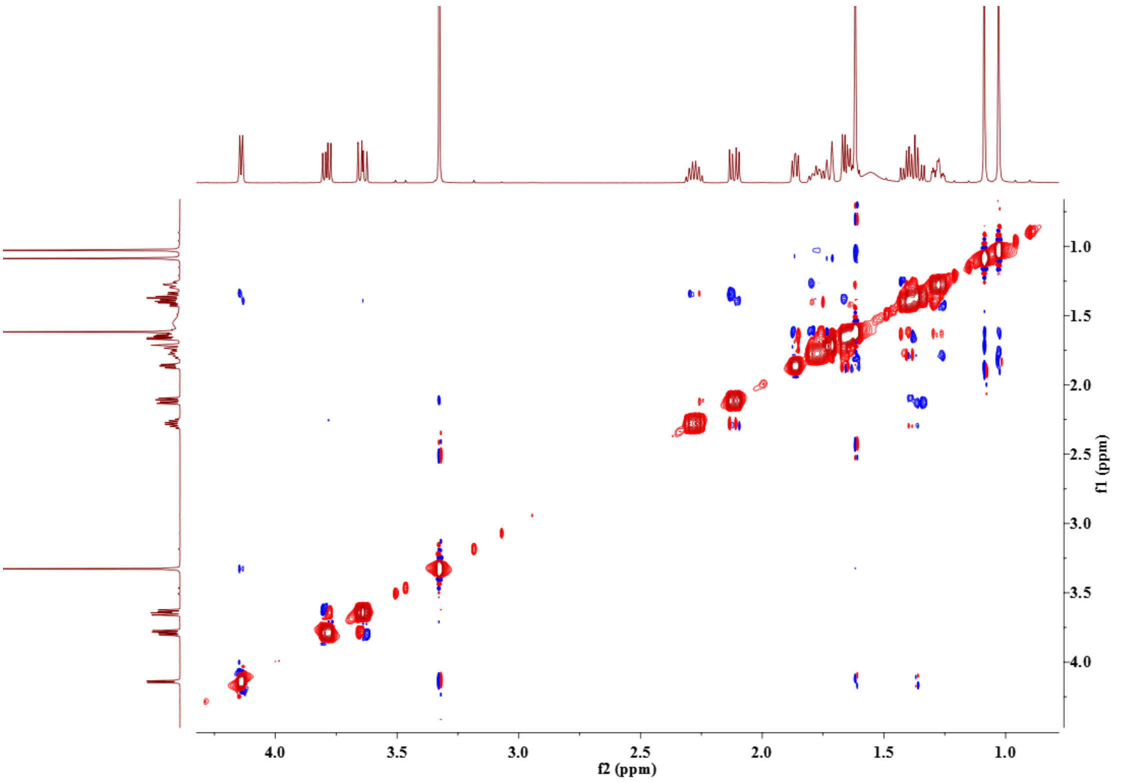

Figure S49. ROESY spectrum of compound **7**

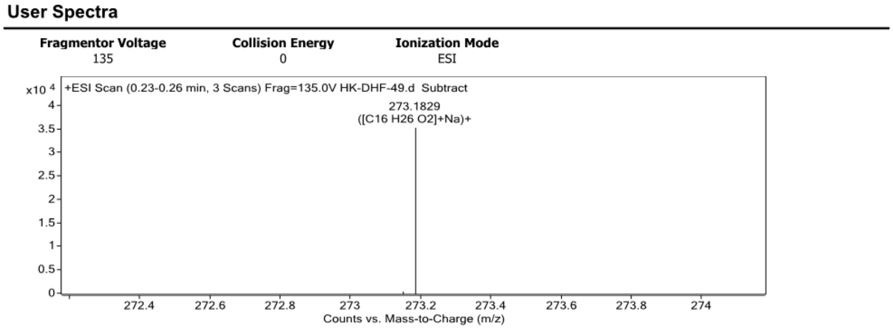

Peak List

m/z	z	Abund	Formula	Ion
145.1009	1	2739.77		
190.1295		3036.43		
201.1638	1	14200.6		
219.1745	1	40278.71		
220.1776	1	6287.46		
273.1829	1	35410.25	C16 H26 O2	(M+Na)+
274.1862	1	5950.43	C16 H26 O2	(M+Na)+
274.2743	1	5214.67		

Formula Calculator Element Limits

Element	Min	Max
C	3	60
H	0	120
O	0	30

Formula Calculator Results

Formula	CalculatedMass	CalculatedMz	Mz	Diff. (mDa)	Diff. (ppm)	DBE
C16 H26 O2	250.1933	273.1825	273.1829	-0.40	-1.46	4.0000

Figure S50. HRESIMS spectrum of **8**

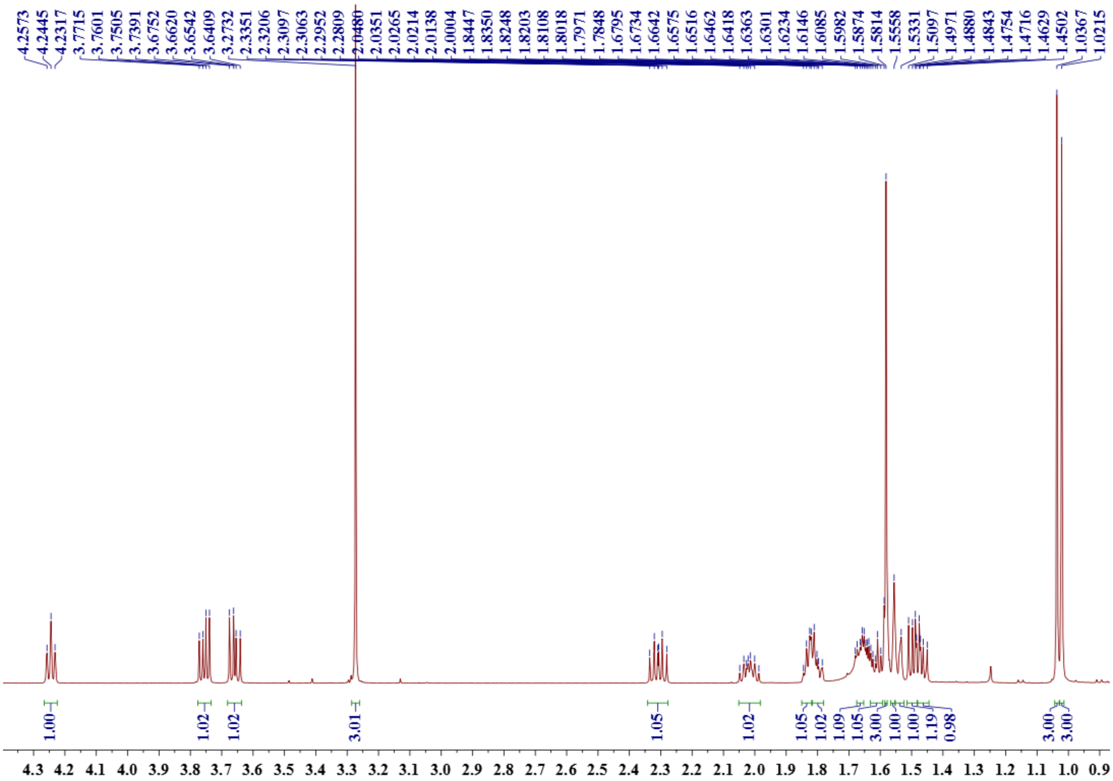

Figure S51. ^{1}H NMR spectrum of compound **8** in $CDCl_3$ (500 MHz)

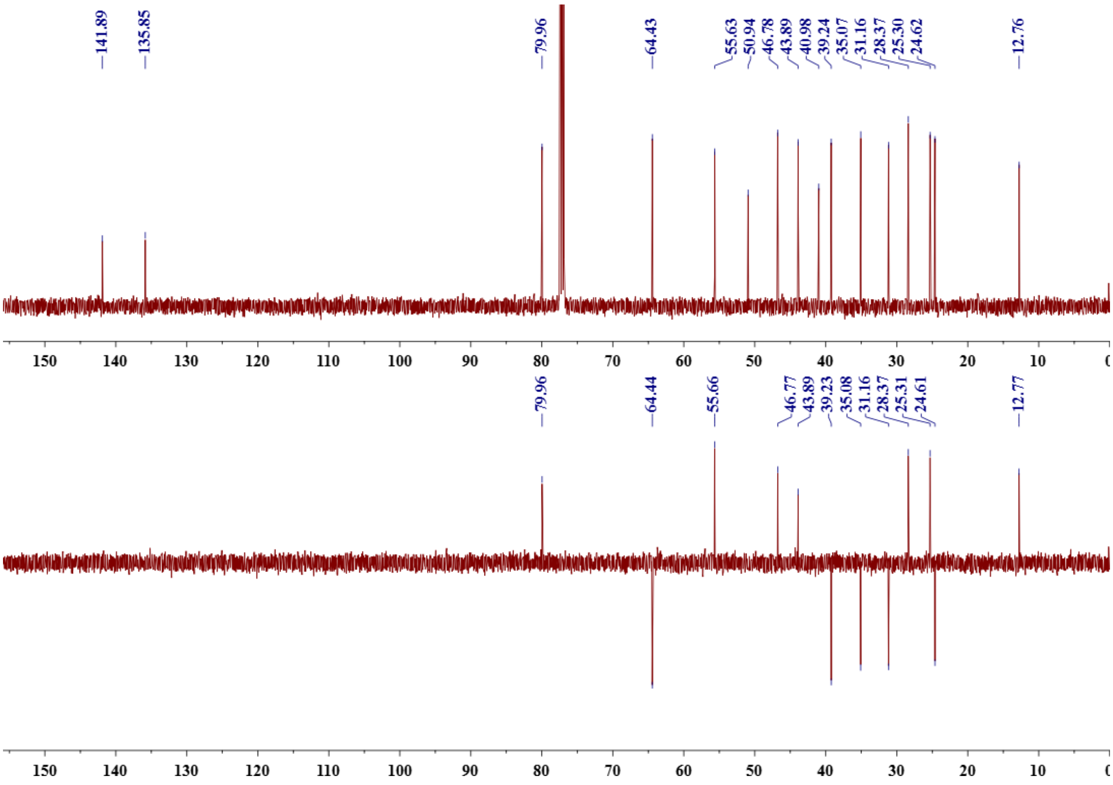

Figure S52. ^{13}C NMR spectrum of compound **8** in $CDCl_3$ (125 MHz)

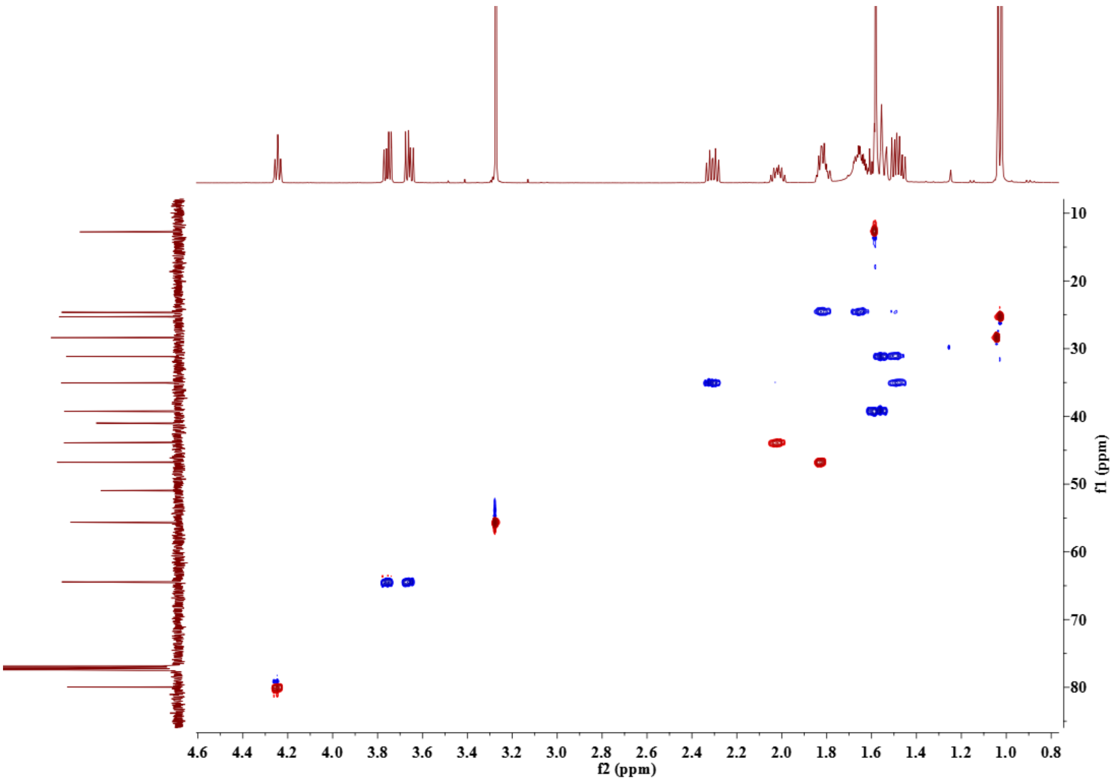

Figure S53. HSQC spectrum of compound **8**

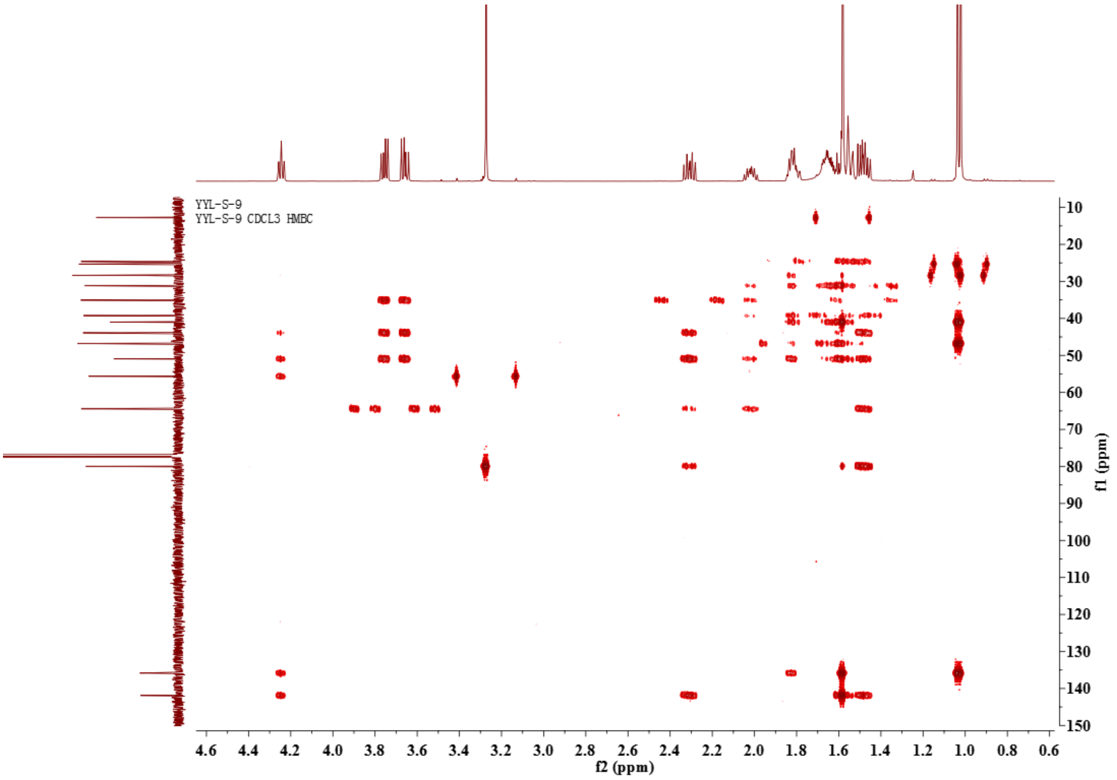

Figure S54. HMBC spectrum of compound **8**

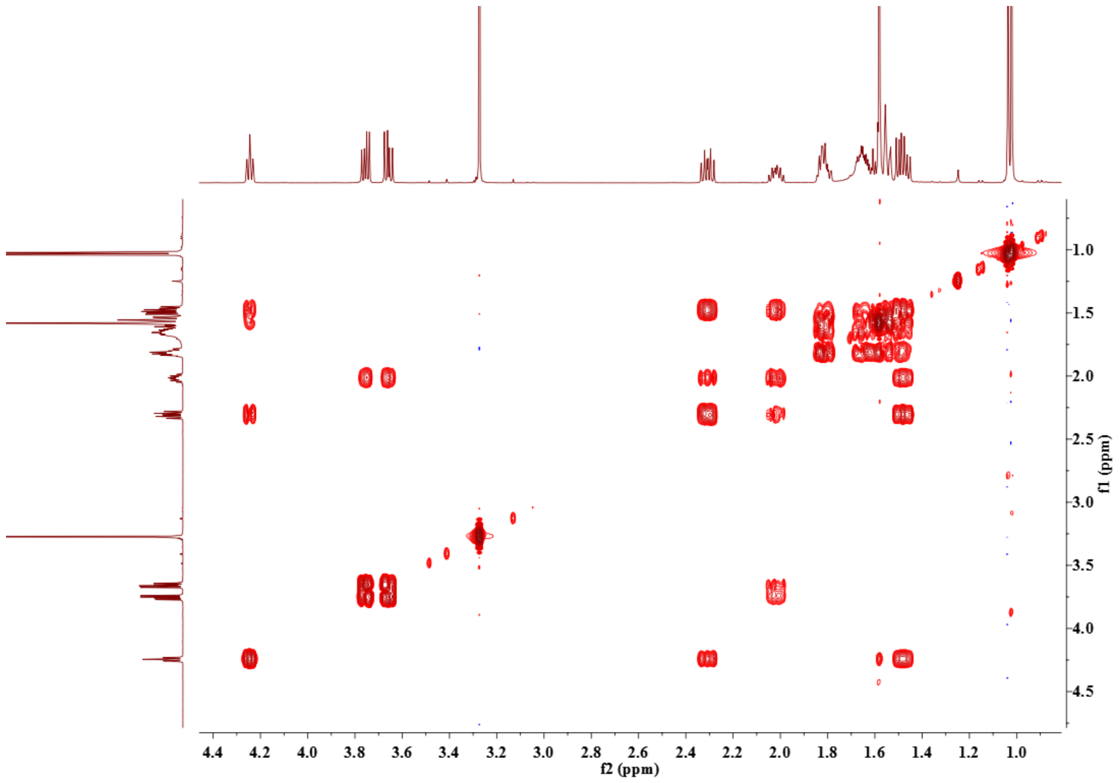

Figure S55. ^{1}H-^{1}H COSY spectrum of compound **8**

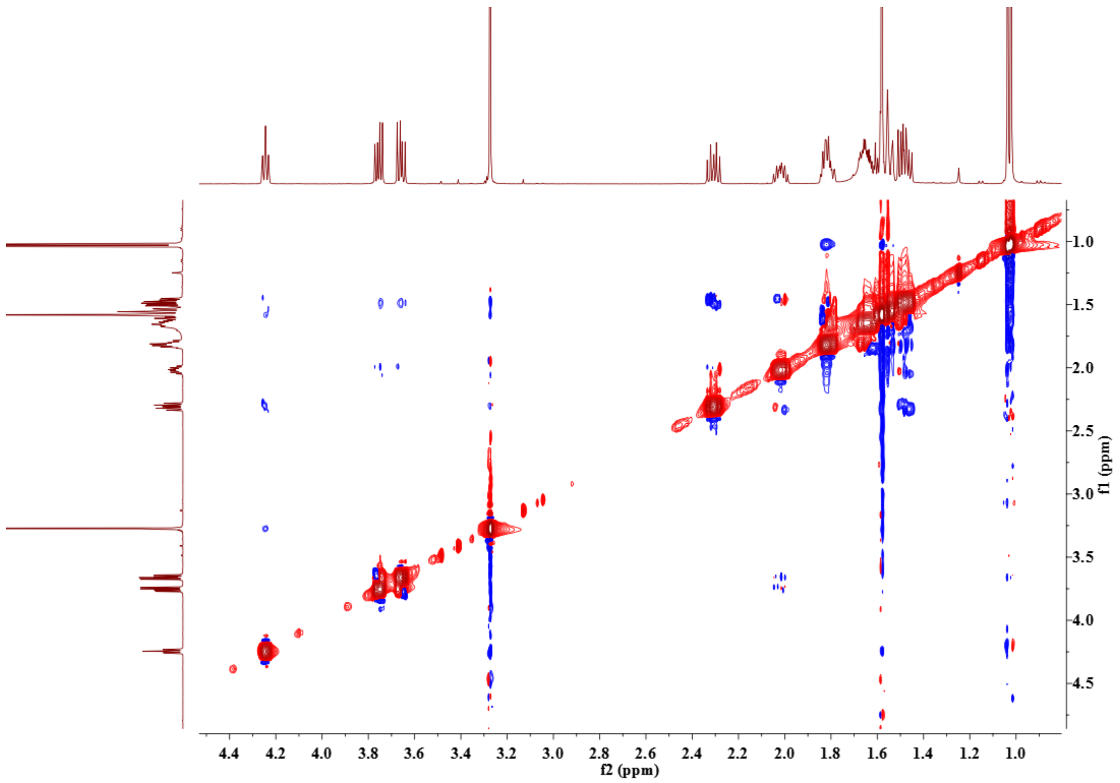

Figure S56. ROESY spectrum of compound **8**

User Spectra

Fragmentor Voltage	Collision Energy	Ionization Mode
135	0	ESI

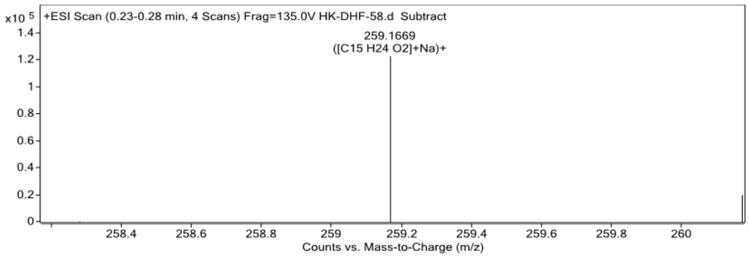

Peak List

m/z	z	Abund	Formula	Ion
219.1743	1	33303.75		
259.1669	1	122920.6	C15 H24 O2	(M+Na)+
260.1703	1	20384.51	C15 H24 O2	(M+Na)+
274.2741	1	15150.8		
290.2691	1	12515.46		
437.1936	1	13224.49		
453.1675	1	10605.36		
495.3442	1	13294.85		

Formula Calculator Element Limits

Element	Min	Max
C	3	60
H	0	120
O	0	30

Formula Calculator Results

Formula	CalculatedMass	CalculatedMz	Mz	Diff. (mDa)	Diff. (ppm)	DBE
C15 H24 O2	236.1776	259.1669	259.1669	0.00	0.00	4.0000

Figure S57. HRESIMS spectrum of **9**

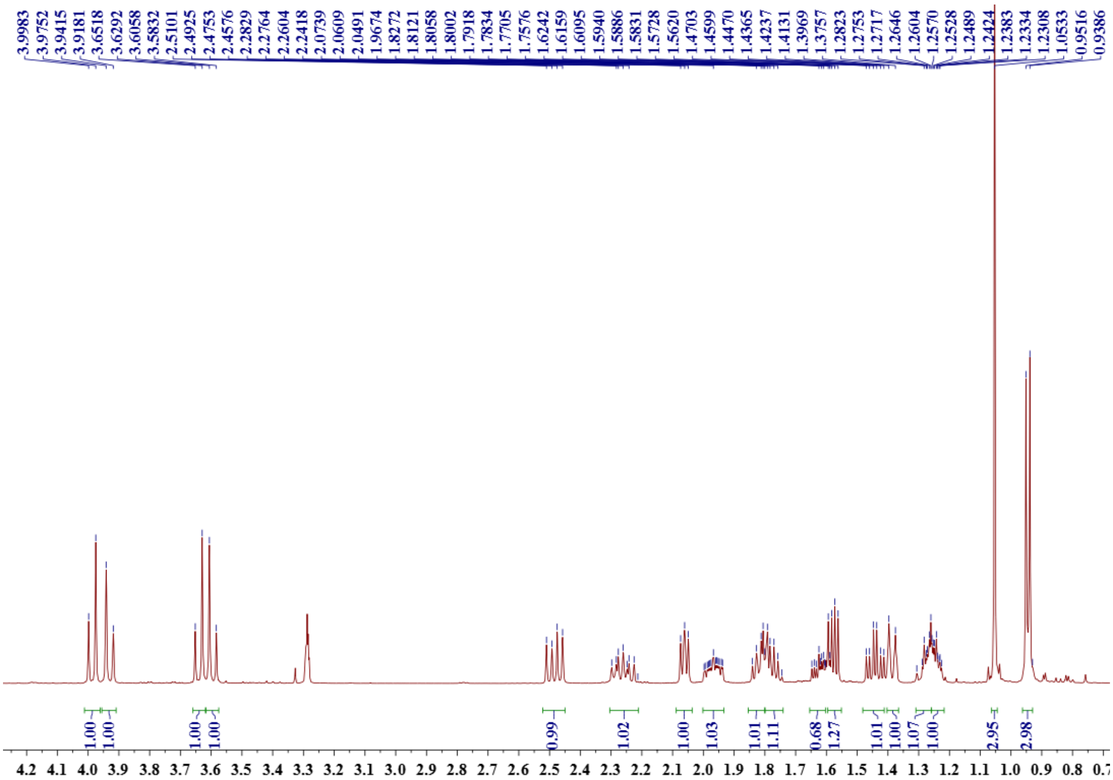

Figure S58. ^{1}H NMR spectrum of compound **9** in MeOH-d_4 (500 MHz)

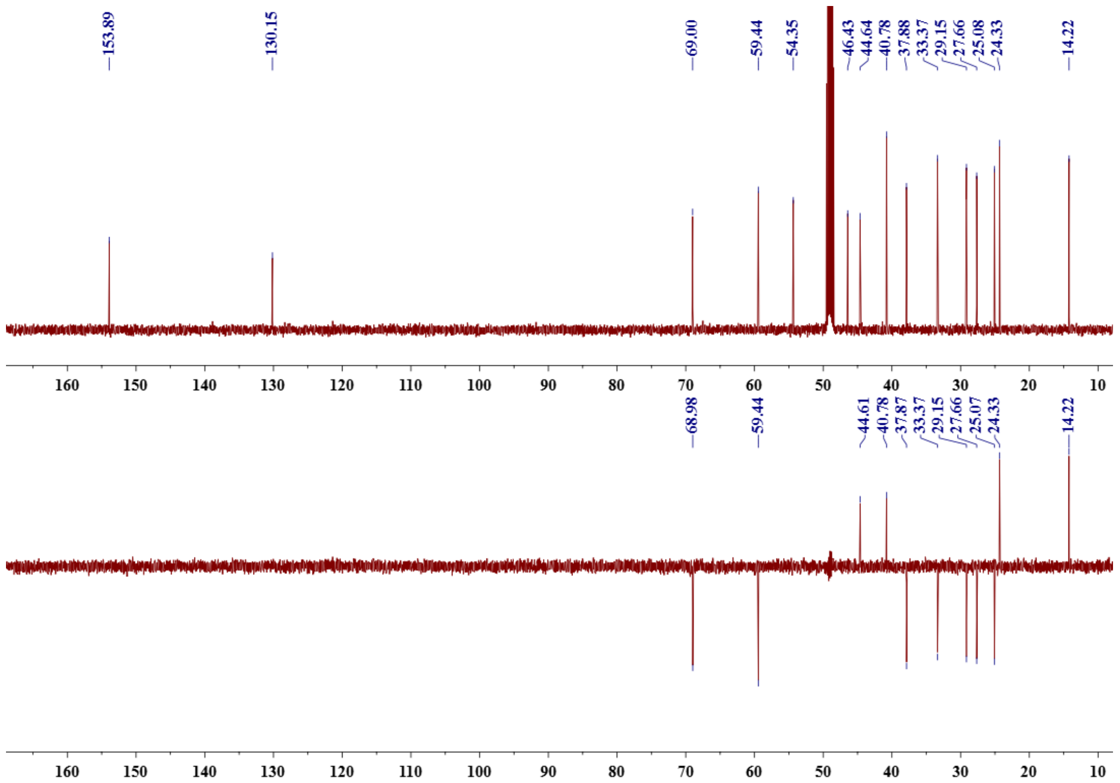

Figure S59. ^{13}C NMR spectrum of compound **9** in MeOH-d_4 (125 MHz)

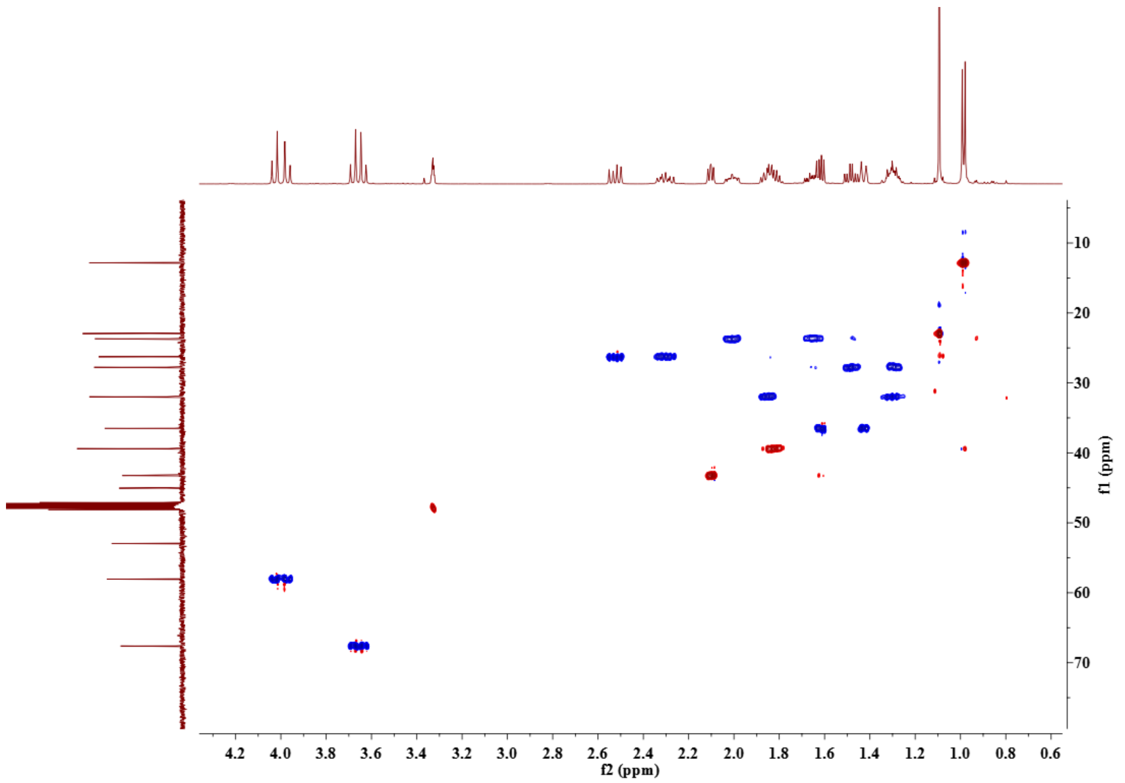

Figure S60. HSQC spectrum of compound **9**

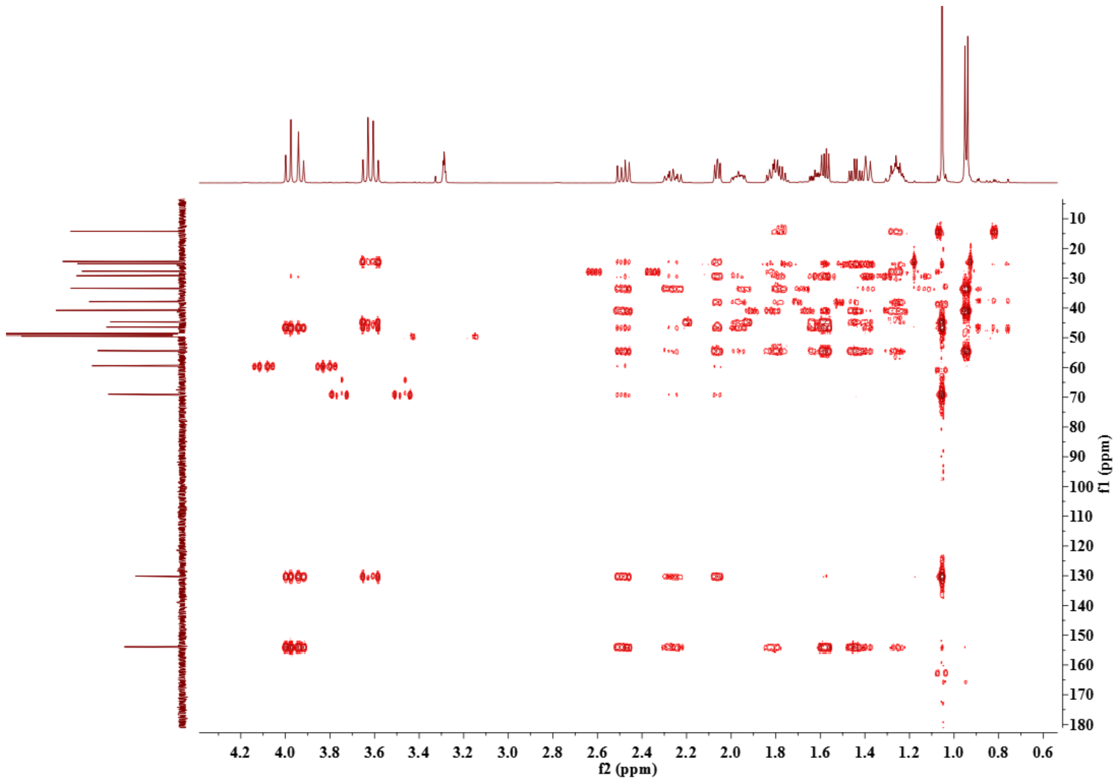

Figure S61. HMBC spectrum of compound **9**

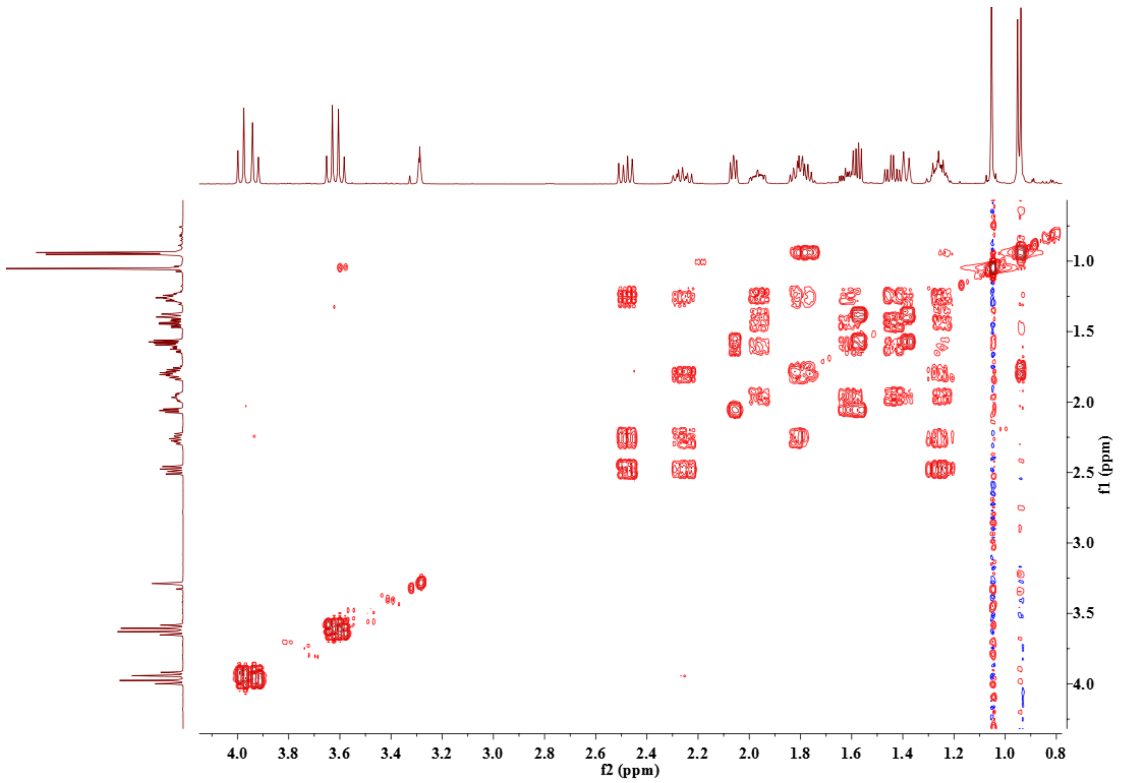

Figure S62. ^{1}H-^{1}H COSY spectrum of compound **9**

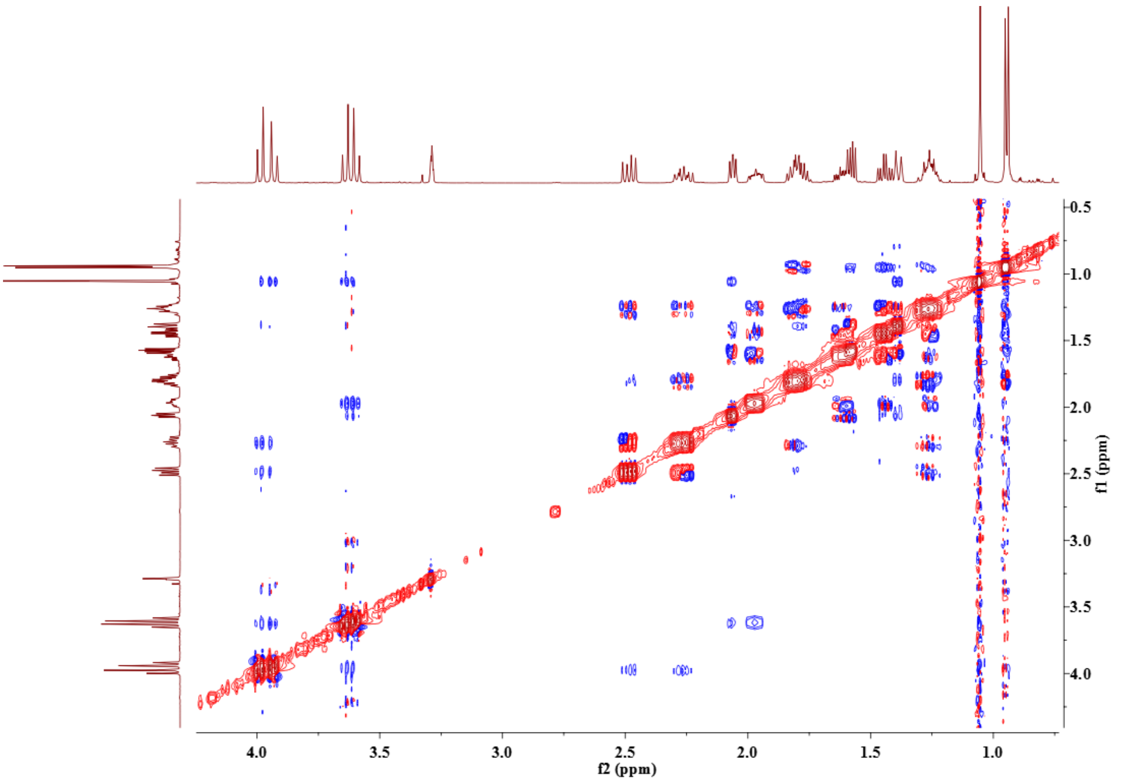

Figure S63. ROESY spectrum of compound **9**

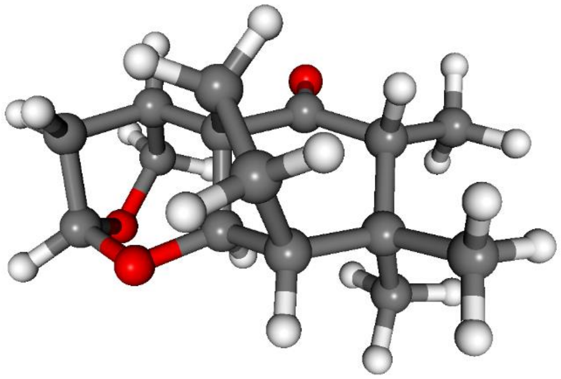

Figure S64. Single low-energy conformer of (1*S*,2*S*,4*S*,6*R*,8*R*,11*R*)-**3** computed at the CAM-B3LYP/TZVP PCM/MeOH level.

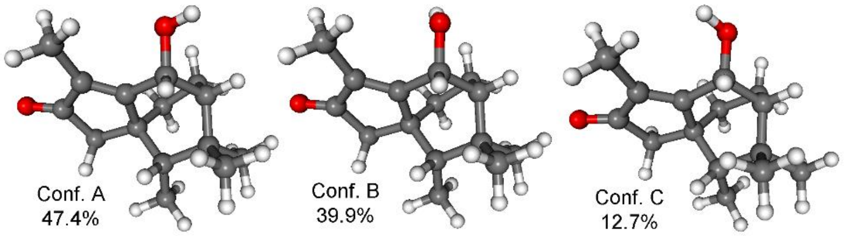

Figure S65. Low-energy conformers and populations of (5*R*,6*S*,8*R*,11*R*)-**1** computed at the SOGGA11-X/TZVP SMD/MeOH level.

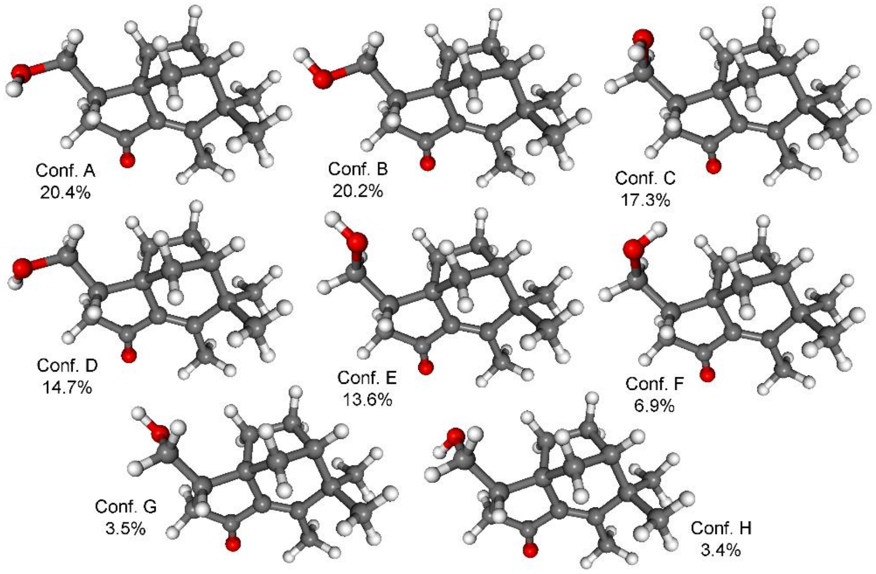

Figure S66. Low-energy conformers and populations of (1*R*,2*S*,8*S*)-**4** computed at the CAM-B3LYP/TZVP PCM/MeOH level.
